# Prognostic Factors in Limited-Stage Small Cell Lung Cancer: A Secondary Analysis of CALGB 30610–RTOG 0538

**DOI:** 10.1001/jamanetworkopen.2024.40673

**Published:** 2024-10-01

**Authors:** Michael K. Farris, Michael D. Mix, Xiaofei Wang, Brandy Jaszewski, Nathan Foster, Gregory A. Masters, Fran Laurie, Koren Smith, Niema B. Razavian, Ryan S. Alden, Ritsuko Komaki, Thomas E. Stinchcombe, Jeffrey D. Bradley, Everett E. Vokes, Jeffrey Bogart

**Affiliations:** Department of Radiation Oncology, Wake Forest Medical University, Winston-Salem, North Carolina; Department of Radiation Oncology, SUNY Upstate Medical University, Syracuse, New York; Alliance Statistics and Data Management Center, Duke University, Durham, North Carolina; Alliance Statistics and Data Management Center, Mayo Clinic, Rochester, Minnesota; Alliance Statistics and Data Management Center, Mayo Clinic, Rochester, Minnesota; Helen F. Graham Cancer Center, Newark, Delaware; Department of Radiation Oncology, University of Massachusetts Chan Medical School, Worcester, Massachusetts; Department of Radiation Oncology, University of Massachusetts Chan Medical School, Worcester, Massachusetts; Department of Radiation Oncology, Wake Forest Medical University, Winston-Salem, North Carolina; Department of Radiation Oncology, SUNY Upstate Medical University, Syracuse, New York; University of Texas M. D. Anderson Cancer Center, Houston; Division of Medical Oncology, Duke Cancer Institute, Duke University, Durham, North Carolina; Department of Radiation Oncology, Perelman School of Medicine, University of Pennsylvania, Philadelphia; Department of Medicine, University of Chicago, Chicago, Illinois; Department of Radiation Oncology, SUNY Upstate Medical University, Syracuse, New York

## Abstract

**IMPORTANCE:**

The impact of patient-specific, disease-related, and social factors on outcomes in limited-stage small cell lung cancer (LS-SCLC) is not well defined. A post hoc secondary analysis of such factors from the Cancer and Leukemia Group B (CALGB) 30610–Radiation Therapy Oncology Group (RTOG) 0538 trial may impact future trial design.

**OBJECTIVE:**

To assess the comprehensive demographic, disease-related, treatment-related, and social factors for potential associations with survival outcomes and understand whether specific subpopulations may benefit from radiotherapy (RT) dose escalation in LS-SCLC.

**DESIGN, SETTING, AND PARTICIPANTS:**

This post hoc secondary analysis of a randomized clinical trial included 638 adults with LS-SCLC treated at 186 unique treatment sites with at least 1 accrual for all patients from March 15, 2008, to December 1, 2019; 313 patients were randomized to receive RT twice daily to a dosage of 45 Gy for 3 weeks and 325 to receive RT once daily to a dosage of 70 Gy for 7 weeks. Data were locked February 28, 2022, and analyzed from November 28, 2022, to June 4, 2024.

**INTERVENTIONS:**

Twice-daily RT or once-daily RT.

**MAIN OUTCOMES AND MEASURES:**

Multivariable Cox proportional hazards models evaluated the association of treatment groups and other risk factors with progression-free survival (PFS) and overall survival (OS). Patient-specific factors included age, sex, and Eastern Cooperative Oncology Group performance status. Disease-related factors included tumor, nodal, and overall cancer stages. Treatment-related factors included type of chemotherapy, timing of concurrent RT, RT technique, and prophylactic cranial irradiation. Social factors included marital status and treatment center accrual volume.

**RESULTS:**

Among 507 patients (260 [51.3%] female and 247 [48.7%] male; mean [SD] age, 62.6 [7.9] years) included in the multivariate survival analysis, with a median follow-up of 4.7 (IQR, 3.7–7.1) years, female sex was associated with improved OS (hazard ratio [HR], 0.73 [95% CI, 0.58–0.91]; *P* = .006), while being 70 years or older was associated with decreased OS (HR, 1.50 [95% CI, 1.141.98]; *P* = .004). Neither age nor sex was associated with PFS. When compared with those with N1 disease, OS and PFS were worse in patients with N2 (HRs, 1.64 [95% CI, 1.19–2.26]; *P* = .002 and 1.36 [95% CI, 1.02–1.81]; *P* = .04, respectively) and N3 (HRs, 2.03 [95% CI, 1.40–2.93]; *P* < .001 and 1.63 [95% CI, 1.17–2.26]; *P* = .004) disease. Compared with stage II cancer, OS was worse for stage IIIA (HR, 1.65 [95% CI, 1.17–2.31]; *P* = .004) and stage IIIB (HR, 1.94 [95% CI, 1.34–2.83]; *P* < .001). Compared with high-volume accrual centers, treatment at low- or middle-volume accrual centers was associated with worse PFS (HRs, 1.94 [95% CI, 1.33–2.82; *P* < .001] and 1.44 [95% CI, 1.15–1.82; *P* = .002], respectively) and worse OS (HRs, 1.55 [95% CI, 1.03–2.32; *P* = .03] and 1.33 [95% CI, 1.04–1.70; *P* = .02], respectively).

**CONCLUSIONS AND RELEVANCE:**

This secondary analysis of the CALGB 30610–RTOG 0538 randomized clinical trial of patients with LS-SCLC found associations between female sex or being younger than 70 years and improved overall survival and between advanced nodal stage or treatment at low- or middle-volume accrual centers and worse outcomes. These findings suggest that stratification by nodal stage, clinical stage, and age should be considered in future randomized trials.

**TRIAL REGISTRATION:**

ClinicalTrials.gov Identifier: NCT00632853

## Introduction

Small cell lung cancer (SCLC) accounts for approximately 13% of lung cancers, and limited-stage SCLS (LS-SCLC) is typically managed with chemotherapy and concurrent radiotherapy (RT).^1^ The INT0096 trial established twice-daily radiotherapy to 45 Gy as the superior regimen compared with once-daily RT to 45 Gy.^2^ However, twice-daily RT fractionation schedules are infrequently used in clinical practice due to a variety of logistical issues, patient and physician preferences, and/or institutional capacity.^3^ As a result, dose escalation with once-daily RT has been investigated in several randomized clinical trials.^4–6^

In the Cancer and Leukemia Group B (CALGB) 30610–Radiation Therapy Oncology Group (RTOG) 0538 trial, patients with LS-SCLC were randomized to receive RT twice daily to a dosage of 45 Gy for 3 weeks or RT once daily to a dosage of 70 Gy for 7 weeks.^4^ While the final analysis did not demonstrate superiority of once-daily RT over twice-daily RT in terms of overall survival (OS) or progression-free survival (PFS), the comprehensive data on demographic, disease-related, treatment-related, and social factors collected present opportunities to further describe potential associations with survival outcomes and understand whether specific subpopulations may benefit from dose escalation.

## Methods

CALGB 30610–RTOG 0538 was a prospective randomized multisite clinical trial in which 638 patients with LS-SCLC at 186 unique treatment sites received chemotherapy and were randomized to concurrent twice-daily or once-daily RT. Each participant signed an informed consent document approved by the participating institutional review boards in accordance with federal and institutional guidelines. The Alliance Data and Safety Monitoring Board reviewed safety data semiannually. This secondary analysis followed the Strengthening the Reporting of Observational Studies in Epidemiology (STROBE) reporting guideline.

**Figure 1.**
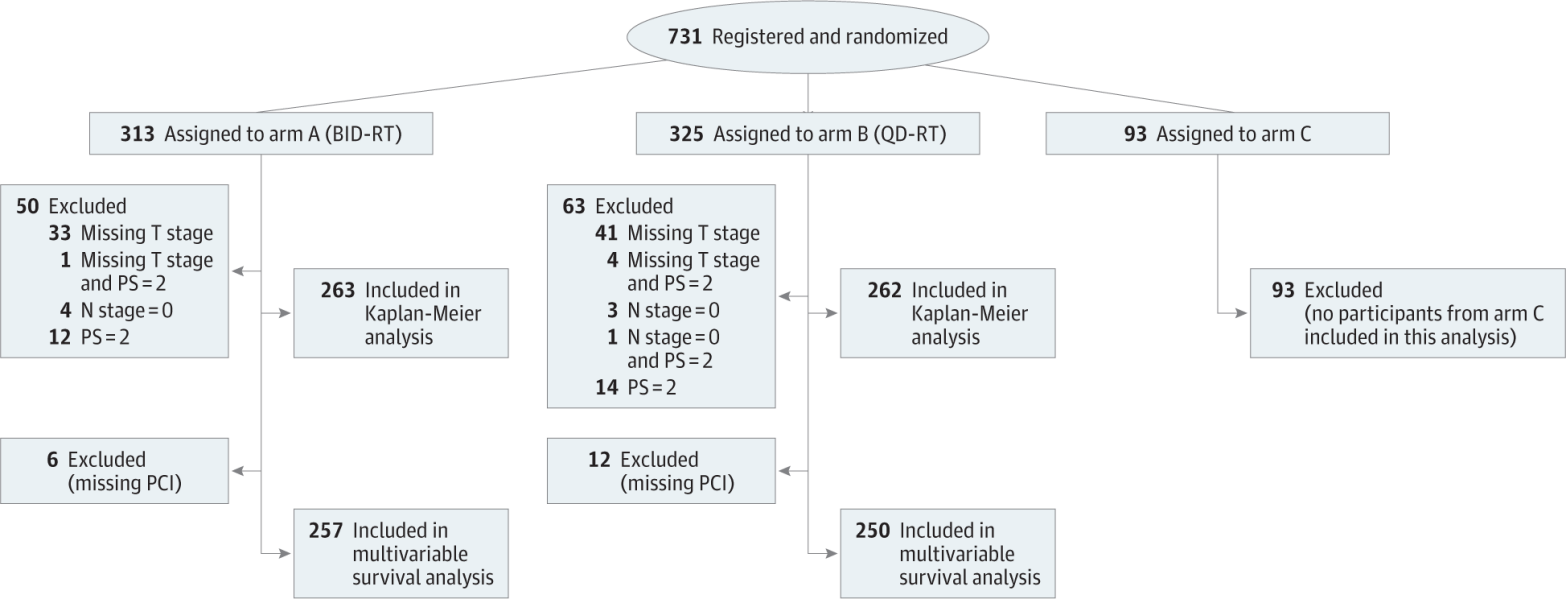
Participant Flow Diagram Patients were registered and randomized at the same time. An Eastern Cooperative Oncology Group performance score (PS) of 2 indicates that the patient is ambulatory and capable of all self-care but unable to carry out any work activities and is up and about more than 50% of waking hours. BID-RT indicates twice-daily radio therapy (RT); PCI, prophylactic cranial irradiation; and QD-RT, once-daily RT.

Patients were stratified by sex, weight loss (6 months prior to study entry ≤5% vs >5% of body weight), Eastern Cooperative Oncology Group performance status (0 vs 1 vs 2; a score of 0 indicates fully active, able to carry on all predisease performance without restriction; a score of 1 indicates restricted in physically strenuous activity but ambulatory and able to carry out work of a light or sedentary nature; a score of 2 indicates that the patient is ambulatory and capable of all self-care but unable to carry out any work activities and is up and about more than 50% of waking hours), RT planning technique (intensity-modulated RT [IMRT] vs 3-dimensional RT [3D-RT]), chemotherapy backbone (cisplatin vs carboplatin), and RT starting time (cycle 1 vs 2). Additional disease characteristics including tumor and nodal stages and *AJCC Cancer Staging Manual*, 7th edition, stage were not required at enrollment but were determined by the authors. Treatment centers were divided based on total cumulative accrual volume: low (1 patient), middle (2–9 patients), and high (≥10 patients). PFS was defined as time from randomization to disease progression or death from any cause; OS, time from randomization to death from any cause. A participant flow diagram is provided in Figure 1.

### Statistical Analysis

**Figure 2.**
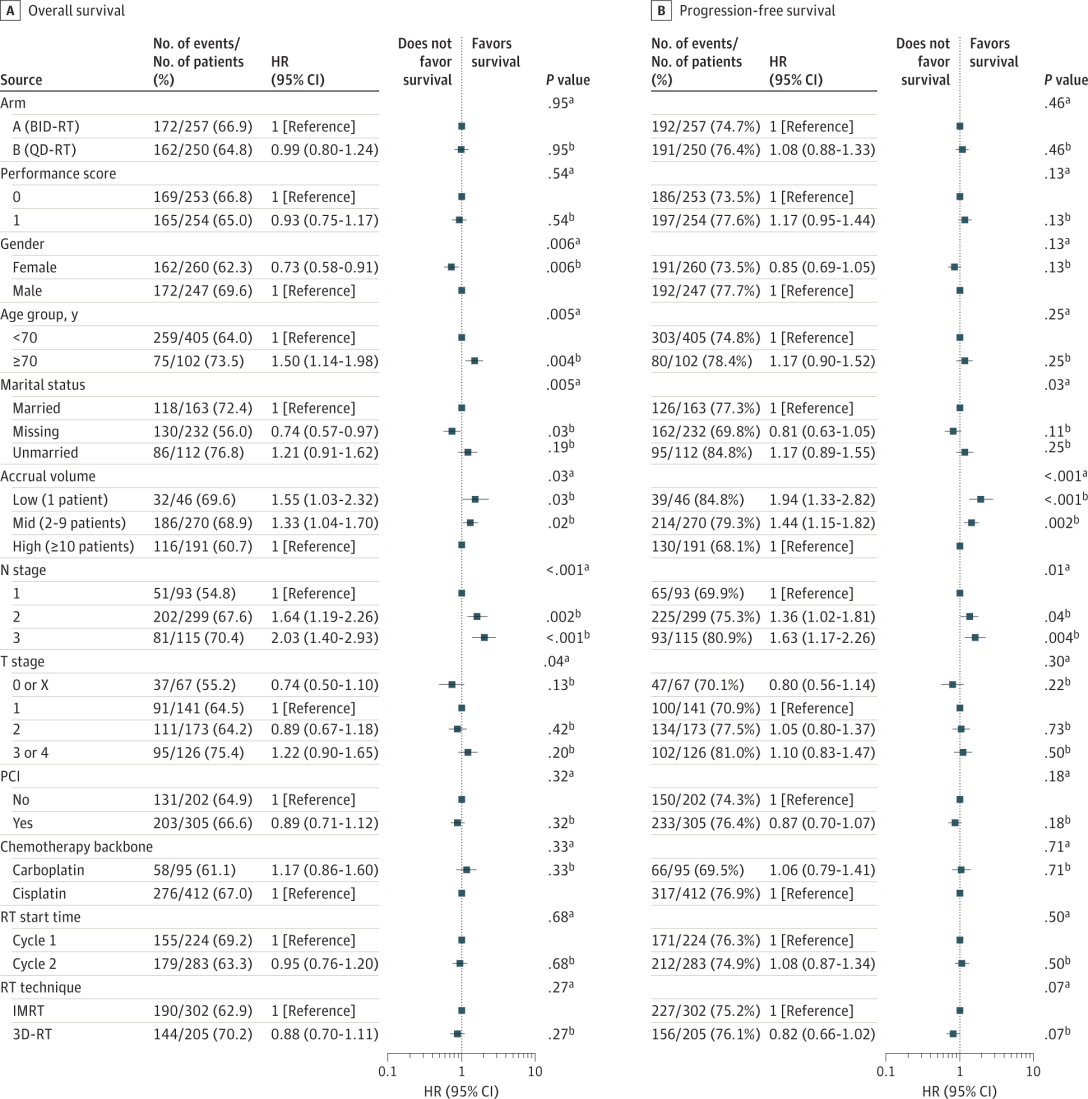
Multivariable Overall (OS) and Progression-Free Survival (PFS) Based on Multivariate Analysis of Patient Factors Arm A received twice-daily radiotherapy (RT) to a total dose of 45 Gy; arm B, once-daily RT to a dose of 70 Gy (QD-RT). An Eastern Cooperative Oncology Group performance score of 0 indicates fully active, able to carry on all predisease performance without restriction; a score of 1 indicates restricted in physically strenuous activity but ambulatory and able to carry out work of a light or sedentary nature. HR indicates hazard ratio; IMRT, intensity-modulated RT; PCI, prophylactic cranial irradiation; and 3D-RT, 3-dimensional RT. ^a^ Calculated using type 3 likelihood ratio test. ^b^ Calculated using covariate Wald test.

Data were analyzed from November 28, 2022, to June 4, 2024. Associations between patient, disease-related, treatment, and social factors and PFS and OS were examined. Initial analyses used univariate Cox proportional hazards models to assess the association between individual factors and survival. Factors displaying an association in univariate analysis were incorporated into a multivariate Cox proportional hazards model, allowing for adjustment of confounding variables and inclusion of factors independently associated with survival outcomes. *P* values from Wald tests for univariate models and likelihood ratio tests for multivariate models were reported. Hazard ratios (HRs) and the corresponding 95% CIs were estimated from the multivariate Cox proportional hazards model. Kaplan-Meier survival analysis was used to generate survival curves and to estimate median survival times. Subgroup analyses were conducted to explore heterogeneity in treatment effects across patient subpopulations defined by patient, disease-related, treatment, and social factors. Difference in survival was analyzed using log-rank tests, and HRs and their 95% CIs were estimated from univariate Cox proportional hazards models. Patients with missing values in the baseline risk factors were excluded. Descriptive statistics were reported when patient demographic data were summarized by treatment arm. Associations of continuous variables with treatment arm were assessed via Wilcoxon rank sum tests and associations of categorical variables via χ^2^ tests. Subset analyses did not require all variables as in the multivariate assessment; thus, patient numbers differ from those given in Figure 2.

Statistical significance was defined as a 2-sided *P* < .05. All statistical analyses were performed by the Alliance Statistics and Data Management Center using SAS, version 9.4 (SAS Institute Inc). Data were locked February 28, 2022. The trial protocol and statistical analysis plan for CALGB 30610–RTOG 0538 are provided in Supplement 1.

## Results

Between March 15, 2008, and December 1, 2019, 507 patients with LS-SCLC (260 [51.3%] female and 247 [48.7%] male; mean [SD] age, 62.6 [7.9] years) received RT (257 twice-daily RT and 250 once-daily RT), had data available for tumor and nodal staging, and were included in this secondary multivariable analysis. Median follow-up was 4.7 (IQR, 3.7–7.1) years. Patient demographic characteristics and tumor characteristics of those included in the multivariable analysis are provided in Figure 2.

### Patient-Specific Factors

**Figure 3.**
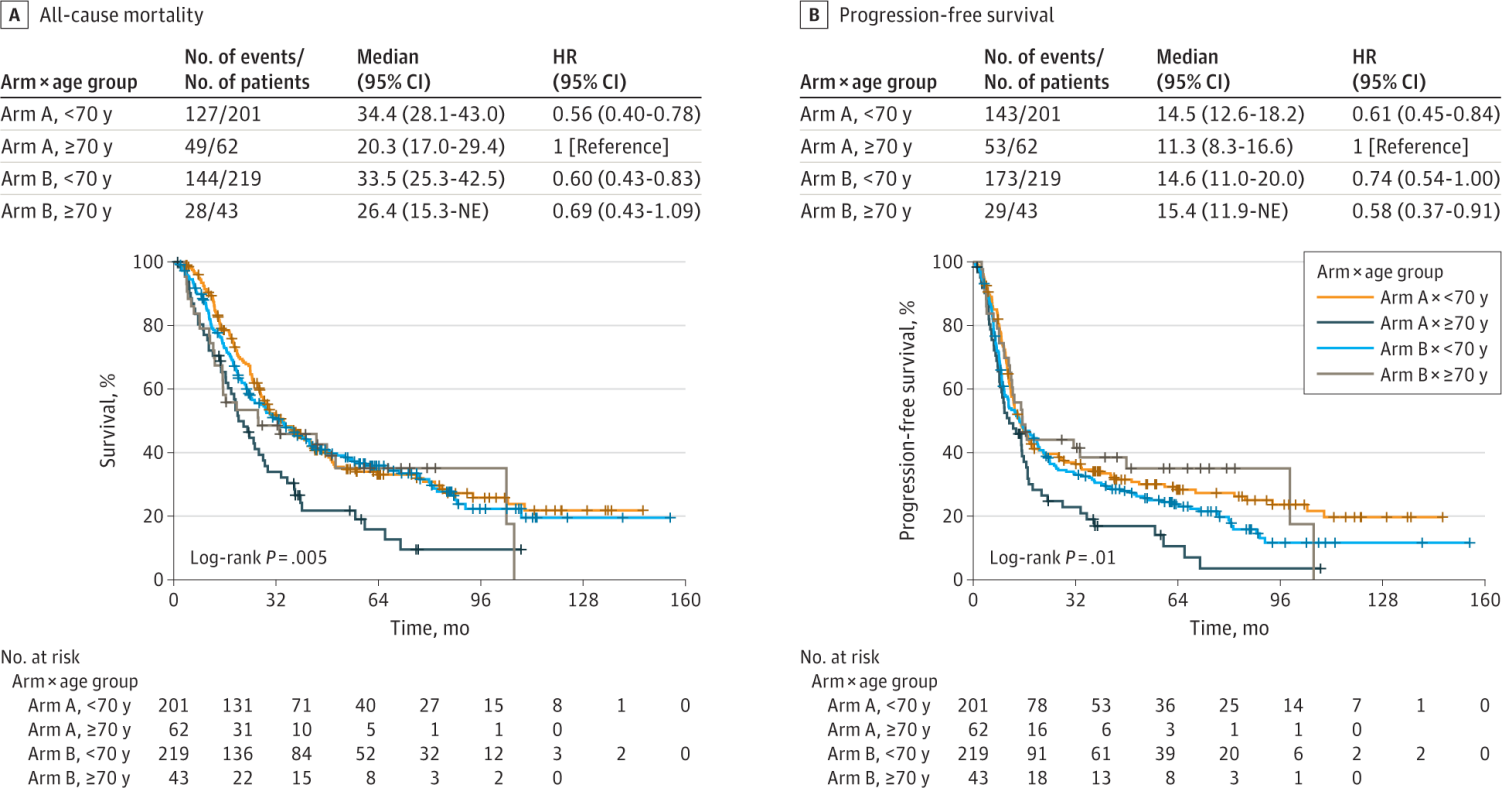
Overall Survival and Progression-Free Survival Among Patients Categorized by Treatment Arm and Age Arm A received twice-daily radiotherapy to a total dose of 45 Gy; arm B, once-daily radiotherapy to a dose of 70 Gy. Plus signs represent censored patients. HR indicates hazard ratio; NE, nonestimable.

Multivariate analysis demonstrated that OS after chemoradiation therapy was associated with sex and age. Women had improved OS (HR, 0.73 [95% CI, 0.58–0.91]; *P* = .006), while advanced age (≥70 years vs <70 years) was associated with worse OS (HR, 1.50 [95% CI, 1.14–1.98]; *P* = .004). Neither factor was associated with PFS. Unplanned subset analysis demonstrated that patients younger than 70 years receiving twice-daily RT had significantly better OS (Figure 3) (HR, 0.56 [95% CI, 0.40–0.78]; *P* < .001) and PFS (HR, 0.61 [95% CI, 0.45–0.84]; *P* = .002) compared with older patients receiving twice-daily RT. Older patients receiving once-daily RT demonstrated improved PFS compared with older patients receiving twice-daily RT (HR, 0.58 [95% CI, 0.37–0.91]; *P* = .02). Weight loss prior to randomization and performance status were not associated with survival outcomes.

### Disease-Related Factors

While tumor category was not associated with OS or PFS, nodal category and overall stage emerged as factors associated with both OS and PFS. Compared with stage II disease (n = 77), OS was worse for the 307 patients with stage IIIA disease (HR, 1.65 [95% CI, 1.17–2.31]; *P* = .004) and the 141 with stage IIIB disease (HR, 1.94 [95% CI, 1.34–2.83]; *P* < .001). Compared with patients with N1 disease, OS was significantly poorer in patients with N2 (HR, 1.64 [95% CI, 1.19–2.26]; *P* = .002) and N3 (HR, 2.03 [95% CI, 1.40–2.93]; *P* < .001) disease. Similarly, compared with N1 disease, PFS was reduced in patients with N2 (HR, 1.36 [95% CI, 1.02–1.81]; *P* = .04) and N3 (HR, 1.63 [95% CI, 1.17–2.26]; *P* = .004) disease. There was no significant difference in PFS or OS when comparing N2 and N3 disease.

### Social Factors

Marital status was not associated with PFS or OS. Treatment at a low-volume center was associated with worse OS (HR, 1.55 [95% CI, 1.03–2.32]; *P* = .03) and PFS (HR, 1.94 [95% CI, 1.33–2.82]; *P* < .001) compared with treatment at a high-volume center. Similarly, treatment at a middle-volume center was associated with worse OS (HR, 1.33 [95% CI, 1.04–1.70]; *P* = .02) and PFS (1.44 [95% CI, 1.15–1.82]; *P* = .002) when compared with a high-volume center.

### Treatment-Related Factors

None of the treatment-related factors investigated were significantly associated with PFS or OS differences. These factors included type of chemotherapy, timing of RT initiation, IMRT vs 3D-RT, twice-daily RT vs once-daily RT, or use of prophylactic cranial irradiation (PCI).

## Discussion

Our findings in this secondary analysis of the CALBG 30610–RTOG 0538 trial highlight the importance of reevaluating factors traditionally associated with outcomes in LS-SCLC. In the future, more refined stratifications should be selected in LS-SCLC trials.

### Patient-Specific Factors

Both female sex and being younger than 70 years were associated with improved OS, but neither was associated with PFS differences. Our finding that older patients may fare worse with twice-daily RT runs in contrast to data from the CONVERT (Concurrent Once-Daily Versus Twice-Daily Radiotherapy) trial.^7^ While just over 100 patients 70 years or older were included in our analysis, it represents, to our knowledge, the largest cohort so far in a prospective randomized clinical trial. We investigated numerous potential explanations for worse outcomes in older patients undergoing twice-daily RT and significant differences in any grade 3 or greater toxicity, RT interruptions, or IMRT vs 3D-RT were not detected. Potentially, undetected deconditioning during twice-daily RT may have delayed or prevented the completion of systemic therapy in a population with frailty. Clearly, further data are needed to better assess the potential association between age and RT regimen.

### Disease-Related Factors

The association of nodal staging with both PFS and OS reinforces the value of nuanced staging beyond historical designations of *limited* and *extensive*. This is particularly relevant given a recent CONVERT post hoc analysis, which similarly underlined the significance of overall stages II vs III disease.^8^

### Social Factors

Our findings echo existing literature and emphasize potential benefits of treatment at high-volume centers.^9,10^ Reasons are likely multifactorial and difficult to discern from available data. Potential explanations include funding and capacity for supportive care, enhanced capacity to detect and address recurrences, or inherent differences in experience or access to multidisciplinary peer discussions.^11–13^

### Treatment-Related Factors

Treatment-specific factors including 3D-RT vs IMRT, RT timing, chemotherapy backbone, and PCI, were not associated with survival. Further analysis of RT treatment details is planned.

### Limitations

This study has limitations, including the retrospective assignment of staging from the *AJCC Cancer Staging Manual*, 7th edition, and, while efforts were made to collect follow-up information on PCI, data submission was not mandated.

## Conclusions

This secondary analysis of CALGB 30610–RTOG 0538 randomized clinical trial comparing twice-daily RT with once-daily RT found that female sex and being younger than 70 years were associated with improved OS and advanced nodal stage and treatment at low- or middle-volume accrual centers were associated with worse outcomes in patients with LS-SCLC. Our study highlights the need for consideration of evidence-based patient and clinical factors during the design of randomized clinical trials in LS-SCLC, including overall clinical stage and nodal category, sex, and patient age.

## Supplementary Material

Supplement 1

Supplement 2

## Data Availability

See Supplement 2.

## Key Points

### Question

What patient-specific, disease-related, and social factors are associated with overall survival and progression-free survival in patients with limited-stage small cell lung cancer?

### Findings

In this post hoc secondary analysis of the CALGB 30610–RTOG 0538 randomized clinical trial, which included 638 patients randomized to twice- or once-daily radiotherapy, female sex and being younger than 70 years were associated with improved overall survival. Advanced nodal stage and treatment at low- or middle-volume accrual centers were associated with worse outcomes.

### Meaning

Future clinical trials in limited-stage small cell lung cancer should consider stratification by gender, age, nodal stage, and treatment center volume to better understand and optimize treatment outcomes.

## References

[1] Govindan R, Page N, Morgensztern D, Changing epidemiology of small-cell lung cancer in the United States over the last 30 years: analysis of the surveillance, epidemiologic, and end results database. J Clin Oncol. 2006;24 (28):4539–4544. doi:10.1200/JCO.2005.04.4859 17008692

[2] Turrisi AT III, Kim K, Blum R, Twice-daily compared with once-daily thoracic radiotherapy in limited small-cell lung cancer treated concurrently with cisplatin and etoposide. N Engl J Med. 1999;340(4):265–271. doi:10.1056/NEJM199901283400403 9920950

[3] Schreiber D, Wong AT, Schwartz D, Rineer J. Utilization of hyperfractionated radiation in small-cell lung cancer and its impact on survival. J Thorac Oncol. 2015;10(12):1770–1775. doi:10.1097/JTO.0000000000000672 26334750

[4] Bogart J, Wang X, Masters G, High-dose once-daily thoracic radiotherapy in limited-stage small-cell lung cancer: CALGB 30610 (Alliance)/RTOG 0538. J Clin Oncol. 2023;41(13):2394–2402. doi:10.1200/JCO.22.01359 36623230 PMC10150922

[5] Faivre-Finn C, Snee M, Ashcroft L, ; CONVERT Study Team. Concurrent once-daily versus twice-daily chemoradiotherapy in patients with limited-stage small-cell lung cancer (CONVERT): an open-label, phase 3, randomised, superiority trial. Lancet Oncol. 2017;18(8):1116–1125. doi:10.1016/S1470-2045(17)30318–2 28642008 PMC5555437

[6] Grønberg BH, Killingberg KT, Fløtten Ø, High-dose versus standard-dose twice-daily thoracic radiotherapy for patients with limited stage small-cell lung cancer: an open-label, randomised, phase 2 trial. Lancet Oncol. 2021; 22(3):321–331. doi:10.1016/S1470-2045(20)30742–7 33662285

[7] Christodoulou M, Blackhall F, Mistry H, Compliance and outcome of elderly patients treated in the Concurrent Once-Daily Versus Twice-Daily Radiotherapy (CONVERT) trial. J Thorac Oncol. 2019;14(1):63–71. doi:10.1016/j.jtho.2018.09.027 30391573 PMC6328625

[8] Salem A, Mistry H, Hatton M, Association of chemoradiotherapy with outcomes among patients with stage I to II vs stage III small cell lung cancer: secondary analysis of a randomized clinical trial. JAMA Oncol. 2019;5(3):e185335. doi:10.1001/jamaoncol.2018.5335 30520977 PMC6439849

[9] Bradley JD, Hu C, Komaki RR, Long-term results of NRG Oncology RTOG 0617: standard- versus high-dose chemoradiotherapy with or without cetuximab for unresectable stage III non–small-cell lung cancer. J Clin Oncol. 2020;38(7):706–714. doi:10.1200/JCO.19.01162 31841363 PMC7048161

[10] Eaton BR, Pugh SL, Bradley JD, Institutional enrollment and survival among NSCLC patients receiving chemoradiation: NRG Oncology Radiation Therapy Oncology Group (RTOG) 0617. J Natl Cancer Inst. 2016;108(9):108. doi:10.1093/jnci/djw034 PMC6059090 27206636

[11] El Saghir NS, Keating NL, Carlson RW, Khoury KE, Fallowfield L. Tumor boards: optimizing the structure and improving efficiency of multidisciplinary management of patients with cancer worldwide. Am Soc Clin Oncol Educ Book. 2014;34(1):e461–e466. doi:10.14694/EdBook_AM.2014.34.e461 24857140

[12] Farris MK, Razavian NB, Hughes RT, Bridging the communication gaps: a prospective single-arm pilot study testing the feasibility of interdisciplinary radiotherapy planning in locally advanced lung cancer. Acad Radiol. 2023;30(11):2566–2573. doi:10.1016/j.acra.2023.01.019 36759296 PMC10404636

[13] Kallianos KG, Muhoozi BN, Gottschalk A, Dedicated diagnostic radiology/radiation oncology rounds:added value beyond traditional tumor boards. Curr Probl Diagn Radiol. 2020;49(4):248–253. doi:10.1067/j.cpradiol.2019.05.004 31153661

