# Supplementary material for: Prognostic Factors in Limited-Stage Small Cell Lung Cancer: A Secondary Analysis of CALGB 30610–RTOG 0538
Source: JAMA Netw Open. Author manuscript; Available in PMC 2025 Oct 1. (PMC11581554; doi:10.1001/jamanetworkopen.2024.40673)
Supplement: Supplement 1 [file NIHMS2034183-supplement-Supplement_1.pdf]

Protocol Update #14

ALLIANCE FOR CLINICAL TRIALS IN ONCOLOGY

PROTOCOL UPDATE TO CALGB 30610

PHASE III COMPARISON OF THORACIC RADIOTHERAPY REGIMENS IN PATIENTS WITH LIMITED SMALL CELL LUNG CANCER ALSO RECEIVING CISPLATIN OR CARBOPLATIN AND ETOPOSIDE

X	**Update:**		**Status Change:**
X	Eligibility changes		Activation
	Therapy / Dose Modifications / Study Calendar changes		Closure
	Informed Consent changes		Suspension / temporary closure
	Scientific / Statistical Considerations changes		Reactivation
X	Data Submission / Forms changes		
X	Editorial / Administrative changes		
	Other		

Expedited review is allowed. IRB approval (or disapproval) is required within 90 days. Please follow your IRB of record guidelines.

UPDATES TO THE PROTOCOL:

Cover Page
The Respiratory Committee Chair has been updated from Dr. Vokes to Dr. Stinchcombe.
A new Staff Statistician has been added.
The Data Manager has been updated from Andrew Johnson to Katie Hervey.

CTSU Address and Contact Information Page
The information under "Submit study data to" has been updated.

Section 4.3 Prior Treatment
The 4th sentence of the first bullet point: "Registration to CALGB 30610 must take place within 7-21 days after the start of the non-protocol therapy" has been moved to Section 5.4 of the registration section. This is no longer in the eligibility section.

Section 5.0 Patient Registration
The entire section has been modified with the updated CTSU language.

Section 5.5 Stratification
The radiotherapy start time language has been clarified.

Section 6.1 Data Submission
The first four paragraphs in this section have replaced the previous data submission language.
In the paragraph following the table information about using CTCAE version 5 for **serious** AE reporting has been added.

Section 16.0 Adverse Event Reporting
The references to the CTCAE reporting have been updated from version 4.0 to version 5.0 for serious AE reporting. CTCAE version 4.0 will continue to be used for routine AE reporting.

Section 16.2 Additional Instructions or Exclusions from CTEP-AERS Expedited Reporting Requirements
The first bullet point regarding death due to progressive disease has been updated.
The seventh bullet point has new guidelines about reporting pregnancy loss.
The eighth bullet point replaces the previous AML/MDS and new primary malignancy bullets.

UPDATES TO THE MODEL CONSENT:

There are no changes to the model consent.

A replacement protocol and model consent have been issued.

ATTACH TO THE FRONT OF EVERY COPY OF THIS PROTOCOL

Activation Date: March 15, 2008
Includes Update #14

ALLIANCE FOR CLINICAL TRIALS IN ONCOLOGY

CALGB 30610/RTOG 0538

PHASE III COMPARISON OF THORACIC RADIOTHERAPY REGIMENS IN PATIENTS WITH LIMITED SMALL CELL LUNG CANCER ALSO RECEIVING CISPLATIN OR CARBOPLATIN AND ETOPOSIDE

Clinicaltrials.gov Identifier: NCT00632853

Study Chair
Jeffrey Bogart, M.D.
State University of New York Upstate Medical University
750 E. Adams Street
Syracuse, NY 13210
Tel: 315-464-5276
bogartj@upstate.edu

Study Co-chair
Gregory Masters, M.D.
Tel: 302-366-1200
gmasters@cbg.org

RTOG Radiation Oncology Co-chair
Ritsuko Komaki, M.D.
Tel: 713-563-2300
rkomaki@mdanderson.org

RTOG Medical Oncology Co-chair
John Heymach, M.D., Ph.D.
Tel: 713-792-6363
jheymach@mdanderson.org

RTOG Correlative Science Co-chair
Yuhchyau Chen, M.D., Ph.D.
Tel: 585-275-5623
yuhchyauchen@urmc.rochester.edu

CALGB QOL Co-chair
Stewart Fleishman, M.D..
Tel: 212-844-6295
sfleishm@bethisraelny.org

RTOG QOL Co-chair
Benjamin Movsas, M.D.
Tel: 313-916-5188
Bmovsas1@hfhs.org

Respiratory Committee Chair
Thomas Stinchcombe, M.D.
Tel: 919-966-4432
thomas.stinchcombe@duke.edu

Respiratory Correlative Science Committee Chair
Robert Kratzke, M.D.
Tel: 612-626-3794
kratz003@umn.edu

Faculty Statistician
Xiaofei Wang, Ph.D.
Tel: 919-681-5406
xiaofei.wang@duke.edu

Staff Statistician
Junheng Gao, MS
Tel: 919-668-6646
junheng.gao@duke.edu

Data Manager
Katie Hervey
Tel: 507-266-6117
hervey.katie@mayo.edu

Protocol Coordinator
Colleen R.B. Watt
Tel: 773-702-4670
cboyle@uchicago.edu

Participating NCTN Groups: NRG, ECOG-ACRIN, SWOG

Alliance Central Protocol Operations Program Office
125 S. Wacker Dr, Suite 1600
Chicago, IL 60606
Tel: 773-702-9171
Fax: 312-345-0117
www.allianceforclinicaltrialsinoncology.org

Adverse Event Reporting
https://eapps-ctep.nci.nih.gov/ctepaers/

Alliance Biorepository and Ohio State
The Ohio State University
Innovation Centre
2001 Polaris Parkway
Columbus, OH 43240
Tel: 614-293-7073 Fax: 614-293-7967
path.calgb@osumc.edu

Alliance Statistics and Data Center
200 First Street SW, Harrick 8th Floor
Rochester, MN 55905

CALGB 30610 Pharmacy Contact
Carol Hubbard, RPh
Tel: 315-342-6215
hubbardc@upstate.edu

CALGB 30610 Nursing Contact
Gail McCue Donnery, M.S.N., A.R.N.O., A.O.C.N.
Tel: 315-464-8227 Fax: 315-464-8206

CONTACT INFORMATION		
**For regulatory requirements:**	**For patient enrollments:**	**Submit study data to:**
Regulatory documentation must be submitted to the CTSU via the Regulatory Submission Portal.<br><br>Regulatory Submission Portal: (Sign in at www.ctsu.org, and select the Regulatory Submission sub-tab under the Regulatory tab.)<br><br>Institutions with patients waiting that are unable to use the Portal should alert the CTSU Regulatory Office immediately at 1-866-651-2878 to receive further instruction and support.<br><br>Contact the CTSU Regulatory Help Desk at 1-866-651-2878 for regulatory assistance.	Please refer to the patient enrollment section of the protocol for instructions on using the Oncology Patient Enrollment Network (OPEN) which can be accessed at https://www.ctsu.org/OPEN_SYSTEM/ or https://OPEN.ctsu.org.<br><br>Contact the CTSU Help Desk with any OPEN-related questions at ctsucontact@westat.com.	Data collection for this tudy will be done exclusively through Medidata Rave. Please see the data submission section of the protocol for further instructions.
The most current version of the study protocol and all supporting documents must be downloaded from the protocol-specific Web page of the CTSU Member Web site located at https://www.ctsu.org. Access to the CTSU members' website is managed through the Cancer Therapy and Evaluation Program - Identity and Access Management (CTEP-IAM) registration system and requires user log on with CTEP-IAM username and password. Permission to view and download this protocol and its supporting documents is restricted and is based on person and site roster assignment housed in the CTSU RSS.		
**For clinical questions (i.e. patient eligibility or treatment-related)** Contact the Study PI of the Lead Protocol Organization.		
**For non-clinical questions (i.e. unrelated to patient eligibility, treatment, or clinical data submission)** contact the CTSU Help Desk by phone or e-mail:<br><br>CTSU General Information Line – 1-888-823-5923, or ctsucontact@westat.com. All calls and correspondence will be triaged to the appropriate CTSU representative.		
The CTSU Website is located at https://www.ctsu.org.		

PHASE III COMPARISON OF THORACIC RADIOTHERAPY REGIMENS IN PATIENTS WITH LIMITED SMALL CELL LUNG CANCER ALSO RECEIVING CISPLATIN OR CARBOPLATIN AND ETOPOSIDE

Patient Eligibility

Histologically or cytologically documented small cell lung cancer of limited stage.
Measurable disease.
No prior chemotherapy or radiotherapy for SCLC, apart from 1 cycle of chemotherapy (see Section 4.3).
No prior mediastinal or thoracic radiotherapy
Patients with complete surgical resection of disease are not eligible.
Age ≥18 years.
ECOG Performance status 0-2.
Non-pregnant and non-nursing.

Required Initial Laboratory Values

Granulocytes	≥1,500/µl
Platelets	≥100,000/µl
Total Bilirubin	≤1.5 x ULN
AST (SGOT)	≤2.0 x ULN
Serum Creatinine	≤ 1.5 x ULN
OR	
Calculated CrCl	≥ 70 ml/min

Schema (1 cycle = 21 days)

Patients will receive 4 cycles of chemotherapy

Part II: Based on the results of Part I, the experimental arm (Arm C) was discontinued and patients are randomized (as of 03/11/2013) as follows:

Register/ Randomize →

Arm A:
Radiotherapy (every day, Monday-Friday, for a total of 3 weeks)
XRT: 45 Gy BID (1.5 Gy/fx) starting on day 1 of Cycle 1 or 2, every day, for 3 weeks
Chemotherapy (every 21 days for 4 cycles, for a total of 12 weeks)
Cisplatin 80 mg/m^2 IV on day 1 **OR Carboplatin** AUC 5 IV day 1, every 21 days
Etoposide 100 mg/m^2 IV on days 1, 2, and 3, every 21 days

Arm B:
Radiotherapy (every day, Monday-Friday, for a total of 7 weeks)
XRT: 70 Gy QD (2.0 Gy/fx), starting on day 1 of Cycle 1 or 2, every day, for 7 weeks
Chemotherapy (every 21 days for 4 cycles, for a total of 12 weeks)
Cisplatin 80 mg/m^2 IV on day 1 **OR Carboplatin** AUC 5 IV day 1, every 21 days
Etoposide 100 mg/m^2 IV on days 1, 2, and 3, every 21 days

Prophylactic cranial irradiation (PCI) should be offered to all patients with a complete or near CR.

EFFECTIVE MARCH 10, 2013, PART I IS CLOSED TO FURTHER ACCRUAL

Part I:

Register/ Randomize →

Arm A: Same as Arm A in Part II

Arm B: Same as Arm B in Part II

Arm C:
Radiotherapy (every day, Monday-Friday, for a total of 5 weeks)
XRT: 61.2 Gy Concomitant boost: QD (1.8 Gy/fx), starting on day 1 of Cycle 1 or 2, every day, for 16 days of treatment; then BID (1.8 Gy/fx) for 9 days of treatment
Chemotherapy (every 21 days for 4 cycles, for a total of 12 weeks)
Cisplatin 80 mg/m^2 IV on day 1, every 21 days
Etoposide 100 mg/m^2 IV on days 1, 2, and 3, every 21 days

TABLE OF CONTENTS

SECTION		PAGE
1.0	Introduction	8
1.1	Thoracic Radiotherapy	8
1.2	Timing of Radiotherapy	10
1.3	Chemotherapy	11
1.4	High Dose Thoracic Radiotherapy Concurrent with Cycle 1 Chemotherapy	11
1.5	Summary	12
1.6	Inclusion of Women and Minorities	13
2.0	Objectives	13
2.1	Primary Objective	13
2.2	Secondary Objectives	13
3.0	On-Study Guidelines	14
4.0	Eligibility Criteria	14
4.1	Documentation of Disease	14
4.2	Measurable Disease	15
4.3	Prior Treatment	15
4.4	Age Requirement	15
4.5	ECOG Performance Status	15
4.6	Non-pregnant and non-nursing	15
4.7	Required Initial Laboratory Values	15
5.0	Patient Registration	15
5.1	CTEP Registration Procedures	15
5.2	CTSU Site Registration Procedures	16
5.3	Patient Registration Requirements	18
5.4	Patient Registration/Randomization Procedures	18
5.5	Stratification	19
5.6	Registration to Companion Studies	19
6.0	Data And Sample Submission	20
6.1	Data Submission	20
6.2	Data Submission for QOL	22
6.3	Specimen Submission for Correlative Studies	22
7.0	Required Data	25
8.0	Treatment Plan	27
8.1	Chemotherapy	27
8.2	Radiation Therapy	28
9.0	Dose Modifications And Management of Toxicity	35
9.1	Hematologic Toxicity	35
9.2	Gastrointestinal Toxicity	36
9.3	Hepatic Toxicity	36
9.4	Nephrotoxicity	36
9.5	Hypomagnesemia	36
9.6	Neurologic Toxicity	36
9.7	Ototoxicity	36
9.8	Allergic Reactions	37

9.9 Grade 3/4 Non-Hematologic Toxicity ... 37
9.10 Radiotherapy Dose Modifications for In-field Non-Hematologic Toxicities ... 37
9.11 Dose Modifications for Obese Patients ... 38
10.0 Companion Studies ... 38
10.1 Correlative Science Substudies (CALGB 150712) ... 38
10.2 Health Related Quality of Life (CALGB 70702) ... 43
11.0 Drug Formulation, Availability, And Preparation ... 48
11.1 Cisplatin ... 48
11.2 Carboplatin ... 49
11.3 Etoposide ... 50
12.0 Ancillary Therapy ... 50
12.1 Alliance Policy Concerning the Use of Growth Factors ... 50
13.0 Criteria For Response, Progression, And Relapse ... 51
13.1 Target Lesions ... 51
13.2 Non-target Lesions ... 52
13.3 Cytology and Histology ... 52
13.4 Evaluation of Best Overall Response ... 52
13.5 Guidelines for Evaluation of Measurable Disease ... 53
13.6 Confirmation Measurement/Duration of Response ... 54
14.0 Removal Of Patients From Protocol Therapy ... 54
14.1 Duration of Treatment ... 54
14.2 Extraordinary Medical Circumstances: ... 54
15.0 Statistical Considerations ... 55
15.1 Endpoints ... 55
15.2 Stratification Factors ... 56
15.3 Sample Size with Power Justification ... 56
15.4 Analysis Plan Including Plans for Formal Interim Analysis ... 57
15.5 Logistics and Accrual ... 58
15.6 Correlative Studies (CALGB 150712) ... 59
15.7 Health Related Quality of Life (CALGB 70702) ... 61
15.8 CDUS Reporting ... 66
16.0 Adverse Event Reporting (Aer) ... 67
16.1 CALGB 30610 Adverse Event Reporting Requirements ... 67
16.2 Additional Instructions or Exclusions from CTEP-AERS Expedited Reporting Requirements: ... 68
17.0 References ... 69

1.0 INTRODUCTION

The integration of thoracic radiotherapy (TRT) with systemic chemotherapy for the treatment of limited-stage small cell lung cancer (LSCLC) has been widely studied. Two meta-analyses, published in the early 1990s, confirmed that adding TRT to chemotherapy significantly improved long-term survival for patients with LSCLC [1, 2]. A recent overview of prospective research in LSCLC included 26 randomized clinical trials initiated by cooperative groups in North America between 1972 and 1992 and only 5 studies showed statistically significant survival prolongation in the experimental arm compared with the control arm [3]. All five positive trials studied some aspect of TRT. Although several important questions remain unanswered regarding the optimal integration of TRT and chemotherapy in LSCLC, an Intergroup phase III study addressing limited stage small cell lung cancer study has not been conducted in more than 13 years.

1.1 Thoracic Radiotherapy

Traditionally, modest total doses of radiation, ranging from 45–50 Gy, were employed because of the observed responsiveness of small-cell lung cancer to radiotherapy [4]. Although high clinical response rates are expected with combined modality therapy, durable local tumor control is poor when modest-dose, conventionally fractionated TRT is employed. For example, intrathoracic tumor relapse was observed in the majority of patients treated with 45 Gy once-daily (QD) radiotherapy concurrent with cisplatin and etoposide chemotherapy in a modern prospective phase III trial [5].

Intensifying the radiotherapy course by accelerating the time to complete treatment (while maintaining the same nominal total radiation dose) appears to be an effective strategy in LSCLC. Intergroup trial 0096 (INT 0096) randomized patients to either conventional (180 cGy QD x 25 fractions [45 Gy in 5 weeks]) or hyperfractionated, accelerated (150 cGy twice daily (BID) x 30 fractions [45 Gy in 3 weeks]) TRT [5]. TRT was initiated with the first cycle of etoposide/cisplatin (PE) chemotherapy. Mature results of this trial demonstrate statistically significant improvement in overall survival for the accelerated TRT. Five-year survival was 26% with accelerated RT compared with 16% for patients receiving conventional TRT. Patterns of recurrence reflected improved local and local plus distant recurrence rates with accelerated TRT, suggesting that local treatment has a significant impact on ultimate outcome in LSCLC. The cumulative rate of local tumor relapse was 75% with once-daily TRT compared with 42% with twice-daily TRT. The major increased toxicity of the accelerated regimen was a doubling of the grade 3/4 acute esophagitis rate (e.g., 16% vs 32%).

INT 0096 was a well-conducted phase III study demonstrating that altering the administration of radiotherapy could ultimately influence survival. Despite this result, the 45 Gy BID TRT regimen has not been well accepted in clinical practice. The Patterns of Care Study published in 2003 noted that fewer than 10% of patients with LSCLC received this regimen, while more than 80% were treated with QD TRT [6]. In addition, recent phase II cooperative group trials in LSCLC have not routinely employed 45 Gy BID TRT. The reluctance to accept accelerated TRT may be in part due to increased acute toxicity and practical issues involved with treating patients twice each day. However, the results of the study have also been questioned due to the inclusion of 45 Gy QD TRT as the standard treatment. The predicted biologic efficacy of 45 Gy QD TRT is poor, and a high dose QD TRT arm (e.g., ≥ 60 Gy), which is commonly employed in clinical practice, was not included in the INT 0096 study.

Alternative strategies for increasing the efficacy of treatment include administering either high dose QD TRT, or giving concomitant boost therapy, where BID treatment is given only during part of the treatment course. The Cancer and Leukemia Group B (CALGB) has extensively studied high-dose QD TRT. CALGB 8837, a phase I study, was designed to determine the maximum-tolerated dose (MTD) of TRT in both standard QD and accelerated BID schedules

[7, 8]. Chemotherapy consisted of three cycles of cisplatin, cyclophosphamide, and etoposide followed by two cycles of cisplatin plus etoposide. TRT was initiated with the 4th cycle of chemotherapy, although the initial treatment volume (e.g., lung volume receiving 40 Gy) included the extent of disease at diagnosis. The TRT volume was reduced to include only the post-chemotherapy volume following 40 Gy. The MTD of BID RT was determined to be 45 Gy in 30 fractions over 3 weeks, while it was judged to be at least 70 Gy in 35 fractions over 7 weeks for daily RT. The median survival for QD TRT was 29.8 months compared with 24 months for BID TRT, and 36% of patients treated with high dose QD RT were alive 6 years after therapy compared with 20% for BID RT. Although the number of patients was small, a dose response was also suggested for patients receiving high dose QD TRT. A subsequent CALGB phase II study (C 39808) employed 70 Gy thoracic RT concurrent with carboplatin and etoposide following 2 cycles of induction paclitaxel and topotecan chemotherapy [9]. This Group-wide study confirmed the feasibility of delivering 70 Gy TRT. The overall toxic effects of therapy were comparable with other recent trials using more modest total doses of TRT, and the incidence of severe esophagitis appeared reduced compared with reports of accelerated TRT. Although a larger proportion of patients on C 39808 had weight loss > 5% prior to enrolling on study (33% versus 18%), outcomes were comparable to the accelerated TRT arm of INT 0096. Moreover, median survival on C 39808 was 31 months when the population was limited to patients with weight loss < 5% prior to diagnosis. Two additional CALGB phase II trials employing 70 Gy TRT have completed accrual, further confirming the acceptance of this regimen.

The Radiation Therapy Oncology Group (RTOG) has studied a concomitant boost (CB) strategy in LSCLC. Concomitant boost radiotherapy has been shown to improve local tumor control in head and neck squamous cell carcinoma compared with standard radiotherapy [10]. The concomitant boost regimen has been accepted in clinical practice for head and neck cancer, which is demonstrated in part by rapid accrual to recent studies combining concomitant boost radiotherapy with chemotherapy (RTOG 0129 and RTOG 0522). This approach allows acceleration of TRT but only requires hyperfractionated TRT during part of the treatment course. Moreover, BID large field TRT can be avoided. A phase I trial has been completed assessing the MTD for concomitant boost TRT in LSCLC (R 9712). TRT was initiated with the first of 4 cycles of PE chemotherapy. Accelerated TRT, 61.2 Gy in 34 fractions of 1.8 Gy/Fx in 5 weeks, with BID TRT during the final 9 treatment days, was determined to be the maximum tolerated dose (MTD) [11]. A subsequent phase II study, RTOG 0239, employs the 61.2 Gy concomitant boost regimen and recently completed accrual [12].

The efficacy of radiotherapy can be predicted by calculating the biologic effective dose (BED) [13]. The BED reflects the tumor type (doubling time), dose per fraction, nominal total dose and may also take into account the time to complete therapy. In comparison to the accelerated 45 Gy BID regimen studied in INT 0096, both the CALGB TRT regimen of 70 Gy QD and the RTOG concomitant boost approach yield substantially higher BEDs. For example, assuming a potential tumor doubling time of 5 days, the predicted increase in BED would range from 1.3 to 1.6 (e.g., 30% to 60% increase in efficacy) for the CALGB and RTOG experimental regimens compared with 45 Gy BID.

Predicted Biologic Effective Dose (BED) of Thoracic Radiotherapy Regimens

TRT Regimen	Nominal Dose	BED	BED_{-Time}	Relative BED
INT 0096:<br>45 Gy (1.5 Gy BID/3 weeks)	45 Gy	52	43	1.0
CALGB:<br>70 Gy (2.0 QD / 7 weeks)	70 Gy	84	63	1.4 – 1.6
RTOG:<br>61.2 Gy (1.8 Gy CB /5 weeks)	61.2 Gy	72	57	1.3 – 1.4

Given the impact of intensified local therapy seen in INT 0096, evaluation of TRT regimens that have the potential to further enhance local tumor control and survival is warranted. Importantly, the experimental regimens may be better tolerated and more practical to deliver than the 45 Gy BID regimen. A positive outcome, reflecting an enhanced therapeutic ratio, should result in widespread acceptance in clinical practice.

1.2 Timing of Radiotherapy

The optimal timing of TRT relative to chemotherapy remains controversial. CALGB 8083 randomly assigned patients to receive initial RT plus chemotherapy, delayed RT plus chemotherapy, or chemotherapy alone [14]. Chemotherapy consisted of cyclophosphamide, etoposide, and vincristine, with doxorubicin substituting for etoposide during later cycles. RT, 50 Gy in five weeks, was administered with the 1st cycle (early) or 4th cycle (delayed) of chemotherapy. Mature results showed that survival with chemotherapy alone was inferior to both TRT arms, and the difference was statistically significant for delayed TRT (p=.002) and approached significance for early TRT (p=.082). No significant difference was observed between early and delayed RT, although there was a trend favoring delayed TRT (p=0.14). Conversely, a phase III trial from the National Cancer Institute of Canada (NCIC) demonstrated a benefit for initiating RT, 40 Gy in 3 weeks, with the 2nd chemotherapy cycle compared with the 6th cycle of chemotherapy [15]. Five-year survival was 20% in the early TRT cohort compared with 11% in the late TRT arm, and the difference was ascribed to a reduction in brain metastases, as local tumor control did not differ between arms. Additional studies have attempted to address the timing of TRT, including a trial from Japan comparing 45 Gy BID TRT during the first cycle of chemotherapy with the same TRT regimen administered following the completion of chemotherapy [16]. Median survival was 27 months with early (concurrent) TRT and 20 months with late (sequential) TRT, and the difference approached statistical significance. Recent meta-analyses have been published addressing this issue [17, 18]. While definitive conclusions cannot be reached, there is general consensus that the early initiation of TRT (e.g., cycle 1-3) may be beneficial, particularly in the context of intensive TRT.

Given that the available evidence supports administration of thoracic radiotherapy with either the first or second cycle of systemic chemotherapy, the study was amended on September 15, 2009. Investigators now choose when radiotherapy will start at the time of patient registration. This will allow additional time for investigators to perform sophisticated radiotherapy treatment planning in select patients without delaying the administration of chemotherapy. The timing of radiotherapy delivery (e.g. cycle 1 vs cycle 2) will be incorporated as a stratification factor. Additionally, as of February 20, 2013, patients will be allowed to receive one cycle of chemotherapy prior to registering on CALGB 30610. These patients will then receive the radiotherapy during their second cycle of chemotherapy (their first cycle of protocol therapy).

1.3 Chemotherapy

SCLC is exquisitely sensitive to systemic chemotherapy with high overall response rates. The combination of cisplatin and etoposide (PE) became standard front-line therapy in the 1980s given its clinical activity and tolerability in combination with concurrent thoracic irradiation. PE proved to be at least equivalent to previous combinations including cyclophosphamide, doxorubicin, and vincristine [19, 20]. Recent attempts to find more efficacious regimens by either adding or substituting agents have been disappointing. A phase III CALGB trial compared paclitaxel/etoposide/cisplatin (PET) with PE as first-line therapy for extensive SCLC [21]. Median survival was similar in both arms (5.9 versus 6 months), but toxic deaths increased from 2.4% with PE to 6.5% with PET. The RTOG conducted a phase II study of PET chemotherapy in LSCLC. TRT, 45 Gy BID, was given concurrent with the first of 4 cycles of PE. While the regimen was active, the authors of the study concluded that the addition of paclitaxel was unlikely to improve survival in LSCLC [22]. The topoisomerase-I inhibitors, topotecan and irinotecan, are active agents against SCLC. Topotecan has been studied in combination with paclitaxel in both extensive and limited SCLC, but phase II trials did not suggest improved outcomes that would warrant study in a phase III setting [23]. In contrast, irinotecan plus cisplatin (IP) was superior to PE in a phase III study for extensive SCLC conducted by the Japanese Cooperative Oncology Group [24]. Confirmatory trials were subsequently designed in North America comparing PE to IP. The preliminary results of the initial confirmatory study were recently reported and there was no statistically significant difference in median time to progression, median survival or 1-year survival between the EP and IP regimens [25]. A second phase III trial testing IP vs PE, coordinated by the Southwest Oncology Group, is ongoing, although the results may not be available in the near future. At this time, there is not an obvious hypothesis regarding the integration of novel systemic therapy that would merit testing in a phase III setting. Importantly, it is necessary to define an optimal TRT regimen for the design of future studies that may test the merit of novel cytotoxic agents and molecular targeted agents.

1.4 High Dose Thoracic Radiotherapy Concurrent with Cycle 1 Chemotherapy

RTOG 0241/CALGB 30202 is a recent experience administering high dose QD TRT during the initial cycle of chemotherapy in LSCLC [26]. This phase I study evaluated 70 Gy TRT in combination with cisplatin (60 mg/m^2 day 1) and escalating doses of irinotecan. As noted in the table below, 2 dose limiting toxicities have been observed out of 15 patients assigned to receive 70 Gy TRT; one grade 4 diarrhea and one grade 4 esophageal spasm.

Although TRT was not administered until the 4th cycle of chemotherapy in CALGB 8837, the initial 40 Gy was delivered to the pre-chemotherapy thoracic tumor volume as measured on the CT scan obtained at the time of trial entry. Thus, an increased volume of functioning lung was irradiated on this trial compared with an approach of immediate chemoradiotherapy. Despite treating the pre-chemotherapy lung volume to 40 Gy, grade 3+ pulmonary toxicity was not encountered.

Prospective trials of locally advanced non-small cell lung cancer provide substantial additional experience integrating high dose QD TRT with cycle 1 chemotherapy. TRT doses ranging between 61 Gy to 74 Gy given in 6.5 to 7.5 weeks have been administered with various chemotherapy regimens (table). The proposed study will mandate early interim analyses of both experimental treatment arms for assessment of treatment related toxic effects of therapy.

Selected Lung Cancer Trials: High Dose Daily Thoracic RT Concurrent with Cycle 1 Chemotherapy

Study	n	Dose/ # fractions	Chemotherapy	Toxic Effects	
RTOG 0241/ CALGB 30202 (26) (phase I LSCLC)	15	70 Gy/35 fx	Cisplatin + Irinotecan	Gr 5 (any)	0%
				Gr 3/4 esph	14%/7%
				Gr 3/4 dysp	7%/0%
RTOG 0117 (27) (phase I/II NSCLC)	39*	74 Gy/37 fx	Carboplatin + Paclitaxel	Gr 5 (any)	5%
				Gr 3/4 esph	15%/0%
				Gr 3/4 dysp	5%/0%
CALGB 30407 (28) (phase II NSCLC)	26*	70 Gy /35 fx	Pemetrexed + Carboplatin	Gr 5 (any)	4%
				Gr 3/4 esph	15%/0%
				Gr 3/4 dysp	4%/4%
CALGB 39801 (29) (phase III NSCLC)	121	66 Gy/ 33 fx	Carboplatin + Paclitaxel	Gr 5 (any)	1%
				Gr.3/4 esph	29%/1%
				Gr 3/4 dysp	9%/2%
RTOG 9410 (30) (phase III NSCLC)	201	63 Gy/34 fx	Cisplatin + Vinblastine	Gr 5 (any)	2%
				Gr 3/4 esph	25%
				Gr 3/4 dysp	11%
SWOG 9504 (31) (phase II NSCLC)	83	61 Gy/33 fx	Cisplatin + Etoposide	Gr 5 (any)	4%
				Gr 3/4 esph	12%/5%
				Gr 3/4 dysp	5%/0%
SWOG 0023 (32) (phase III NSCLC)	473	61 Gy/ 33 fx	Cisplatin + Etoposide	Gr 5 (any)	3%
				Gr 3/4 esph	14%/1%
				Gr 3/4 dysp	3%/1%

NSCLC = non-small cell lung cancer, Gr = grade, esph = esophagitis, dysp= dyspnea

1.5 Summary

In summary, defining an optimal TRT regimen in LSCLC remains critical and will have a major impact on clinical practice. Intergroup study 0096 clearly established that improving the efficacy of thoracic radiotherapy can significantly impact survival in patients with LSCLC. Given the reluctance for practitioners to adopt 45 GY BID TRT, the validity of this regimen needs to be assessed in the context of TRT regimens that have higher predicted biologic efficacy and may have improved tolerability and acceptance. Superior outcomes on an experimental arm would lead to establishing a change in the standard of care for patients with LSCLC. Conversely, if the best outcomes were observed with accelerated 45 Gy BID TRT, then the results of this study would provide convincing and definitive evidence for practitioners to adopt this regimen.

As of December 15, 2012, this trial was updated to include only 2 arms. The Alliance Data Safety and Monitoring Board (DSMB) reviewed adverse events for CALGB 30610 on June 29, 2012. At that time the trial had reached sufficient accrual to trigger a decision regarding dropping one of the experimental arms. Toxicity in the 2 arms was found to be similar in the review, and the DSMB unanimously recommended that the Respiratory Committee decide which arm should continue in conjunction with CTEP. The Alliance Respiratory Committee subsequently reviewed the toxicity data and it was noted that while overall toxicity was similar, three on-study deaths (4.5%) were observed in arm C compared with no on-study deaths in arm B. Based on this review and the study mandate to eliminate one treatment arm, the Alliance Respiratory

Committee proposed dropping arm C. This decision was supported after review and discussion with CTEP representatives.

As of September 15, 2014, the protocol was amended to allow substitution of carboplatin for cisplatin at the discretion of the treating physician, and the choice of chemotherapy will be used as a stratification factor. It has become clear during the past several years that carboplatin and etoposide chemotherapy is a commonly used regimen for patients with limited small cell lung cancer, and several investigators have requested that a carboplatin based regimen be allowed. Though the choice of chemotherapy has been widely discussed, there is not clear data that supports the use of cisplatin over carboplatin in small cell lung cancer. A study randomizing patients with small cell lung cancer to receive either cisplatin or carboplatin (with etoposide), conducted by the Hellenic Cooperative Oncology Group, did not demonstrate a difference in response rates or survival with either regimen (99). Moreover, phase II CALGB studies employing a carboplatin and etoposide backbone concurrent with high dose (7000 cGy) thoracic radiotherapy have accrued well.

1.6 Inclusion of Women and Minorities

Women and minorities will be eligible for this study without alteration in eligibility criteria. There is currently no evidence to suggest that differences in response to treatment exist between groups on the basis of gender or race. Exploratory analyses will be conducted using Cox's proportional hazards model to determine whether treatment differences in survival are consistent across men and women, and are also consistent among races.

2.0 OBJECTIVES

2.1 Primary Objective

To determine whether administering high dose thoracic radiotherapy, 70 Gy (2 Gy once-daily over 7 weeks) or 61.2 Gy (1.8 Gy once-daily for 16 days followed by 1.8 Gy twice-daily for 9 days), will improve median and 2-year survival compared with 45 Gy (1.5 Gy twice-daily over 3 weeks) in patients with limited stage small cell lung cancer.

2.2 Secondary Objectives

2.2.1 To compare treatment related toxic effects of thoracic radiotherapy regimens in patients with limited stage small cell lung cancer.

2.2.2 To compare response rates, failure-free survival and toxicity of thoracic radiotherapy regimens in patients with limited stage small cell lung cancer.

2.2.3 To compare rates of local relapse, distant metastases and brain metastases with these regimens.

2.2.4 To compare patients' quality of life between these treatment regimens in terms of their physical symptoms, physical functioning and psychological state.

2.2.5 To describe the patterns of use of thoracic intensity modulated radiation therapy (IMRT) in patients with limited stage small cell lung cancer.

2.2.6 To examine blood-based biomarkers of response and resistance to cisplatin (or carboplatin) and etoposide.

2.2.7 To evaluate the correspondence between increases in plasma ProGRP concentrations and disease progression/recurrence.

2.2.8 To evaluate the potential for plasma ProGRP concentrations at baseline, after each cycle of chemotherapy and at first evaluation following completion of chemotherapy to predict PFS and OS.

2.2.9 To evaluate the correspondence between longitudinal decreases in plasma ProGRP concentrations and clinical response.

3.0 ON-STUDY GUIDELINES

This clinical trial can fulfill its objectives only if patients appropriate for this trial are enrolled. All relevant medical and other considerations should be taken into account when deciding whether this protocol is appropriate for a particular patient. Physicians should consider the risks and benefits of any therapy, and therefore only enroll patients for whom this treatment is appropriate. Although they will not be considered formal eligibility (exclusion) criteria, physicians should recognize that the following may seriously increase the risk to the patient entering this protocol:

- Psychiatric illness which would prevent the patient from giving informed consent.
- Medical condition such as uncontrolled infection (including HIV), uncontrolled diabetes mellitus or cardiac disease which, in the opinion of the treating physician, would make this protocol unreasonably hazardous for the patient.
- Patients with a “currently active” second malignancy other than non-melanoma skin cancers and carcinoma in situ of the cervix. Patients are not considered to have a “currently active” second malignancy if they have completed therapy and have no evidence of recurrence for at least 5 years.
- Women and men of reproductive potential should agree to use an appropriate method of birth control throughout their participation in this study due to the teratogenic potential of the therapy utilized in this trial. Appropriate methods of birth control include abstinence, oral contraceptives, implantable hormonal contraceptives (Norplant), or double barrier method (diaphragm plus condom).

4.0 ELIGIBILITY CRITERIA

All questions regarding eligibility criteria should be directed to Dr. Jeffrey Bogart or Dr. Gregory Masters. Please note that the Study Chair cannot grant waivers to eligiblity requirements.

4.1 Documentation of Disease

4.1.1 Histologically or cytologically documented small cell lung cancer

4.1.2 Limited stage disease patients, with disease restricted to one hemithorax with regional lymph node metastases, including ipsilateral hilar, ipsilateral and contralateral mediastinal, and ipsilateral supraclavicular lymph nodes.

- Patients with disease involvement of the contralateral hilar or supraclavicular lymph nodes are **not** eligible.
- Patients with pleural effusions that are visible on plain chest radiographs, whether cytologically positive or not, are **not** eligible unless they have a negative thoracentesis.
- Patients with cytologically positive pleural or pericardial fluid, regardless of the appearance on plain x-ray, are **not** eligible.

4.2 Measurable Disease

Patients must have measurable disease, which includes lesions that can be accurately measured in at least one dimension (longest diameter to be recorded) as ≥ 2 cm with conventional techniques or as ≥ 1 cm with spiral CT scan.

4.3 Prior Treatment

- Patients may have received one and only one cycle of chemotherapy prior to enrolling on CALGB 30610, which must have included carboplatin or cisplatin and etoposide. If a patient has had one cycle of cisplatin (or carboplatin)/etoposide prior to registration, the patient must have had all of the prior to registration tests outlined in Section 7.0 prior to starting their first cycle of chemotherapy. Additionally, these patients also must have met all of the eligibility criteria in Section 4.0 prior to receiving the first cycle of chemotherapy. Failing to do all of the above will make the patient NOT eligible for CALGB 30610.
- No prior radiotherapy or chemotherapy (except for the chemotherapy described in the bullet above) for SCLC
- No prior mediastinal or thoracic radiotherapy
- Patients with complete surgical resection of disease are **not** eligible

4.4 Age Requirement

Age ≥ 18 years of age

4.5 ECOG Performance Status

PS = 0-2

4.6 Non-pregnant and non-nursing

No patients that are known to be pregnant or nursing.

4.7 Required Initial Laboratory Values

Granulocytes	≥ 1,500/µl
Platelet Count	≥ 100,000/µl
Total Bilirubin	≤ 1.5 x ULN
AST (SGOT)	≤ 2.0 x ULN
Serum Creatinine	≤ 1.5 x ULN
OR	
Calculated Creatinine Clearance	≥ 70 ml/min

5.0 PATIENT REGISTRATION

5.1 CTEP Registration Procedures

Food and Drug Administration (FDA) regulations and National Cancer Institute (NCI) policy require all individuals contributing to NCI-sponsored trials to register and to renew their registration annually. To register, all individuals must obtain a Cancer Therapy Evaluation Program (CTEP) Identity and Access Management (IAM) account (https://ctepcore.nci.nih.gov/iam). In addition, persons with a registration type of Investigator (IVR), Non-Physician Investigator (NPIVR), or Associate Plus (AP) (i.e. clinical site staff requiring write access to OPEN, RAVE, or TRIAD or acting as a primary site contact) must complete their annual registration using CTEP's web-based Registration and Credential Repository (RCR) (https://ctepcore.nci.nih.gov/rcr). Documentation requirements per registration type are outlined in the table below.

Documentation Required	IVR	NPIVR	AP	A
FDA Form 1572	✔	✔		
Financial Disclosure Form	✔	✔	✔	
NCI Biosketch (education, training, employment, license, and certification)	✔	✔	✔	
HSP/GCP training	✔	✔	✔	
Agent Shipment Form (if applicable)	✔			
CV (optional)	✔	✔	✔	

An active CTEP-IAM user account and appropriate RCR registration is required to access all CTEP and CTSU (Cancer Trials Support Unit) websites and applications. In addition, IVRs and NPIVRs must list all clinical practice sites and IRBs covering their practice sites on the FDA Form 1572 in RCR to allow the following:

- Added to a site roster
- Assigned the treating, credit, consenting, or drug shipment (IVR only) tasks in OPEN
- Act as the site-protocol PI on the IRB approval

Additional information can be found on the CTEP website at < https://ctep.cancer.gov/investigatorresources/default.htm >. For questions, please contact the RCR Help Desk by email at < RCRHelpDesk@nih.gov >.

5.2 CTSU Site Registration Procedures

This study is supported by the NCI Cancer Trials Support Unit (CTSU).

IRB Approval:

Each investigator or group of investigators at a clinical site must obtain IRB approval for this protocol and submit IRB approval and supporting documentation to the CTSU Regulatory Office before they can be approved to enroll patients. Assignment of site registration status in the CTSU Regulatory Support System (RSS) uses extensive data to make a determination of whether a site has fulfilled all regulatory criteria including but not limited to the following:

- An active Federal Wide Assurance (FWA) number
- An active roster affiliation with the Lead Network or a participating organization
- A valid IRB approval
- Compliance with all protocol specific requirements.

In addition, the site-protocol Principal Investigator (PI) must meet the following criteria:

- Active registration status

- The IRB number of the site IRB of record listed on their Form FDA 1572
- An active status on a participating roster at the registering site.

Sites participating on the NCI CIRB initiative that are approved by the CIRB for this study are not required to submit IRB approval documentation to the CTSU Regulatory Office. For sites using the CIRB, IRB approval information is received from the CIRB and applied to the RSS in an automated process. Signatory Institutions must submit a Study Specific Worksheet for Local Context (SSW) to the CIRB via IRB Manager to indicate their intent to open the study locally. The CIRB's approval of the SSW is then communicated to the CTSU Regulatory Office. In order for the SSW approval to be processed, the Signatory Institution must inform the CTSU which CIRB-approved institutions aligned with the Signatory Institution are participating in the study.

5.2.1 Downloading Site Registration Documents

Site registration forms may be downloaded from the CALGB 30610 protocol page located on the CTSU members' website.

- Go to https://www.ctsu.org and log in to the members' area using your CTEP-IAM username and password
- Click on the Protocols tab in the upper left of your screen
- Either enter the protocol # in the search field at the top of the protocol tree, or
- Click on the By Lead Organization folder to expand
- Click on the Alliance link to expand, then select trial protocol (CALGB 30610)
- Click on LPO Documents, select the Site Registration documents link, and download and complete the forms provided.

5.2.2 Requirements for CALGB 30610 Site Registration:

- CTSU Transmittal Sheet (optional)
- IRB approval (For sites not participating via the NCI CIRB; local IRB documentation, an IRB-signed CTSU IRB Certification Form, Protocol of Human Subjects Assurance Identification/IRB Certification/Declaration of Exemption Form, or combination is accepted)
- CTSU RT Facilities Inventory Form

NOTE: Per NCI policy all institutions that participate on protocols with a radiation therapy component must participate in the Radiological Physics Center (RPC) monitoring program. If this form has been previously submitted to CTSU it does not need to be resubmitted unless updates have occurred at the RT facility.

5.2.3 Checking Your Site's Registration Status

You can verify your site registration status on the members' section of the CTSU website.

- Go to https://www.ctsu.org and log in to the members' area using your CTEP-IAM username and password
- Click on the Regulatory tab at the top of your screen
- Click on the Site Registration tab
- Enter your 5-character CTEP Institution Code and click on Go

Note: The status given only reflects compliance with IRB documentation and institutional compliance with protocol-specific requirements as outlined by the Lead Network. It does

not reflect compliance with protocol requirements for individuals participating on the protocol or the enrolling investigator's status with the NCI or their affiliated networks.

5.2.4 Submitting Regulatory Documents

Submit required forms and documents to the CTSU Regulatory Office via the Regulatory Submission Portal, where they will be entered and tracked in the CTSU RSS.

Regulatory Submission Portal: www.ctsu.org (members' area) → Regulatory Tab →Regulatory Submission

When applicable, original documents should be mailed to:

CTSU Regulatory Office
1818 Market Street, Suite 3000
Philadelphia, PA 19103

Institutions with patients waiting that are unable to use the Portal should alert the CTSU Regulatory Office immediately at 1-866-651-2878 in order to receive further instruction and support.

5.3 Patient Registration Requirements

- **Informed Consent:** The patient must be aware of the neoplastic nature of his/her disease and willingly consent after being informed of the procedure to be followed, the experimental nature of the therapy, alternatives, potential benefits, side-effects, risks, and discomforts. If local guidelines and regulations permit the enrollment of persons not able to fulfill all of these requirements, the registering institution is responsible for complying with all local regulations and requirements for obtaining consent from these persons. Modification of the model consent form would be required for this purpose Human protection committee approval of this protocol and a consent form is required.
- HIPAA requirements
- At registration, the choice of chemotherapy (carboplatin vs. cisplatin) must be entered into the OPEN registration system. Following registration, patients must continue on the chemotherapy chosen at randomization.

For patients registered to this trial after one cycle of non-protocol therapy, registration to CALGB 30610 must take place within 7-21 days after the start of the non-protocol therapy.

5.4 Patient Registration/Randomization Procedures

Patient enrollment will be facilitated using the Oncology Patient Enrollment Network (OPEN). OPEN is a web-based registration system available on a 24/7 basis. To access OPEN, the site user must have an active CTEP-IAM account (check at < https://ctepcore.nci.nih.gov/iam >) and a 'Registrar' role on either the LPO or participating organization roster. Registrars must hold a minimum of an AP registration type.

All site staff will use OPEN to enroll patients to this study. It is integrated with the CTSU Enterprise System for regulatory and roster data and, upon enrollment, initializes the patient in the Rave database. OPEN can be accessed at https://open.ctsu.org or from the OPEN tab on the CTSU members' side of the website at https://www.ctsu.org. To assign an IVR or NPIVR as the treating, crediting, consenting, drug shipment (IVR only), or investigator receiving a transfer in OPEN, the IVR or NPIVR must list on their Form FDA 1572 in RCR the IRB number used on the site's IRB approval.

Prior to accessing OPEN, site staff should verify the following:

- All eligibility criteria have been met within the protocol stated timeframes.
- All patients have signed an appropriate consent form and HIPAA authorization form (if applicable).

Note: The OPEN system will provide the site with a printable confirmation of registration and treatment information. Please print this confirmation for your records.

To receive site reimbursement for specific tests and/or bio-specimen submissions, completion dates must be entered in the OPEN Funding screen post registration. Please refer to the protocol-specific funding page on the CTSU members' website for additional information. Timely entry of completion dates is recommended as this will trigger site reimbursement.

Further instructional information is provided on the OPEN tab of the CTSU members' side of the CTSU website at https://www.ctsu.org or at https://open.ctsu.org. For any additional questions contact the CTSU Help Desk at 1-888-823-5923 or ctsucontact@westat.com.

5.5 Stratification

- Gender: male vs. female
- Weight loss (6 months prior to study entry): ≤5% of body weight vs >5% of body weight
- ECOG Performance Status: 0 vs 1 vs 2
- Planned Radiotherapy Technique: IMRT vs. 3D conformal
- Radiotherapy Start Time (choose 1, 2 or 3):

  For patients receiving 1 cycle of non-protocol therapy prior to registration:

  1) Start radiotherapy at the first cycle of protocol chemotherapy, after one cycle of prior non-protocol chemotherapy

  For patients starting treatment right after registration (no non-protocol therapy):

  2) Start radiotherapy at the first cycle of protocol chemotherapy, without prior non-protocol chemotherapy
  3) Start radiotherapy at the second cycle of protocol chemotherapy, without prior non-protocol chemotherapy
- Chemotherapy backbone: carboplatin vs. cisplatin

5.6 Registration to Companion Studies

There are two substudies within CALGB 30610. The correlative science study must be offered to all patients enrolled on CALGB 30610; the quality of life study must be offered to all English-speaking patients enrolled on CALGB 30610 (although patients may opt to not participate). These substudies do not require separate IRB approval. **Note: The quality of life substudy, CALGB 70702, closed to accrual on August 30, 2017. Therefore, no further patients will be enrolled on the quality of life substudy.**

The substudies included within CALGB 30610 are:

- CALGB 150712: Correlative Science Studies in CALGB 30610 (Section 10.1)

  If a patient answers "yes" to "My specimen(s) may be used for the research described above" question #1 in the model consent, they have consented to participate in the substudy described in Section 10.1. The patient should be registered to CALGB 150712 at the same time they are registered to the treatment trial (30610). Samples should be submitted per Section 6.3.
- CALGB 70702: Quality of Life Studies in CALGB 30610 (Section 10.2)

As of August 30, 2017, accrual to CALGB 70702 has been met. **Therefore, after August 30, 2017, not further patients will be registered to CALGB 70702.**

Only English-speaking patients may participate in the QOL companion. If a patient answers “yes” to “I choose to take part in the Quality of Life study and agree to complete the Quality of Life questionnaires” question #5 in the model consent, they have consented to participate in the substudy described in Section 10.2. The patient should be registered to CALGB 70702 at the same time they are registered to the treatment trial (CALGB 30610).

Note: Patients that are registered on CALGB 30610 after they receive one cycle of chemotherapy are NOT eligible for the companion studies.

6.0 DATA AND SAMPLE SUBMISSION

6.1 Data Submission

As of March 15, 2018, this study uses Medidata Rave® for remote data capture (RDC) of all data collection. If necessary, data originally submitted to the SDC electronically can be amended via the Medidata Rave® system

The Rave system can be accessed through the iMedidata portal at https://login.imedidata.com. For additional information regarding account setup or training, please visit the training section of the Alliance website. Forms should be submitted in compliance with the table below, and a copy of the All Forms Packet can be downloaded from the Alliance and CTSU websites.

Site personnel with Rave roles assigned on the appropriate roster may receive a study invitation e-mail from iMedidata. To accept the invitation, site users must log into the Select Login (https://login.imedidata.com/selectlogin) using their CTEP-IAM user name and password, and click on the “accept” link in the upper right-corner of the iMedidata page. Please note, site users will not be able to access the study in Rave until all required Medidata and study specific trainings are completed. Trainings will be in the form of electronic learnings (eLearnings), and can be accessed by clicking on the link in the upper right pane of the iMedidata screen. Personnel who did not receive an invitation should contact the Alliance Service Center.

Users who have not previously activated their iMedidata/Rave account at the time of an initial site registration approval for a study in RSS will also receive a separate invitation from iMedidata to activate their account. Account activation instructions are located on the CTSU website’s Rave tab under the Rave Resource Materials heading (Medidata Account Activation and Study Invitation Acceptance). Additional information on iMedidata/Rave is available on the CTSU members’ website under the Rave tab at www.ctsu.org/RAVE/ or by contacting the CTSU Help Desk at 1-888-823-5923 or by e-mail at ctsucontact@westat.com.

For the most up-to-date data forms, please visit the Alliance Web site at www.allianceforclinicaltrialsinoncology.org.

.

**Form***		**Submission Schedule**
C-1727	30610 On-Study Form	Within one month of registration
C-660	Solid Tumor Measurement Form	
Report	CT scan report (chest and abdomen)	
Report	Bone or PET scan report	
Report	MRI or CT report (brain)	
	Pathological/cytological documentation of SCLC	
	RT Quality Assurance Documentation	See *Radiation Therapy* section
	QOL Data	For patients consenting to 70702: see *Data Submission for QOL* section
C-1728	30610 Treatment and Response Form	Every 2 cycles during protocol treatment (including the non-protocol cycle of chemo, if applicable).
C-660	Solid Tumor Evaluation Form	
Report	CT scan reports	
C-1729	Adverse Event Form	Every cycle during protocol treatment
C-1742	Confirmation of Lost to Follow-up Form	Follow form directions.
C-1730	Follow-up and Response Form	After the end of protocol treatment submit every 3 months for 2 years, then every 6 months for 3 years, then every year for an additional 5 years until progression. After progression submit only the C-1730 every 6 months to report survival, new malignancy, and non-protocol treatment data. Report new malignancy on the C-1001.
C-660	Solid Tumor Evaluation Form	
Report	CT scan reports	
C-1001	New Malignancy Form	

This study will use NCI Common Terminology Criteria for Adverse Events (CTCAE) version 3.0 for routine toxicity reporting on study forms. However, CTCAE version 5.0 will be used for **serious** AE reporting through CTEP-AERS as of April 1, 2018; see Section 16.0.

* Use CALGB Remarks Addenda (C-260) if additional comments are necessary or additional writing space is needed.

6.2 Data Submission for QOL

All participating institutions must ask patients for their consent to participate in the Quality of Life Studies in CALGB 30610 (CALGB 70702), although patient participation is optional. Patients registered to CALGB 30610 after they receive one cycle of chemotherapy will not participate in the QOL study. Rationale and methods for this study are described in Section 10.2. For patients who consent to participate, assessments will be collected at the following time points:

Form		Baseline	Weekly during RT	Week 3*	Week 5*	Week 7*	Week 12*	Week 26*	Week 52*
(Assessment No.)		(#0)		(#3)	(#5)	(#7)	(#12)	(#26)	(#52)
C-1732	FACT-L	X		X	X	X	X	X	X
C-1734	FACT Eating and Swallowing Subscales	X		X	X	X	X	X	X
C-1733	ECOG Acute Esophagitis Scale	X	X	X	X	X	X	X	X
C-1736	Difficulty Swallowing	X	X	X	X	X	X	X	X
C-458	HADS	X		X	X	X	X	X	X
C-903	EQ-5D	X		X	X	X	X	X	X
C-616	Subjective Significance Questionnaire			X	X	X	X	X	X
C-419	QOL Assessment Form	X		X	X	X	X	X	X
C-1735	Treatment Inconvenience			X	X	X			
C-187	Sociodemographic Characteristics	X							

* The assessment should be done at 3, 5, 7, 12, 26 or 52 weeks after the start of radiation therapy. The assessment number entered on the forms should be the same as the week number of the assessment.

6.3 Specimen Submission for Correlative Studies

All participating institutions must ask patients for their consent to participate in the correlative science studies in CALGB 30610 (CALGB 150712), although patient participation is optional. Patients registered to CALGB 30610 after receiving one cycle of chemotherapy will not participate in the correlative science substudy. Rationale and methods for the scientific components of these studies are described in Section 10.1.

6.3.1 Plasma Collection for both Correlative Science Studies (see Section 10.1)

For patients who consent to participate, 20 ml of whole blood (in two 10 ml purple top tubes) will be collected at the following time points:

- Baseline (before treatment initiation)
- Day 1 of Cycles 2, 3 and 4 (pre-chemotherapy),
- At first follow-up after completion of all protocol treatment,
- At each post-treatment follow-up visit (Follow-up is every 3 months for 2 years, then every 6 months for 3 years, and then yearly. Note: If follow-up is completed more often than required a plasma sample is preferred by is not required.)
- At the time of recurrence.

6.3.2 Plasma Sample Processing

The purple top tubes should be kept on ice for no more than 30 minutes after venipuncture. Spin down the blood at 1500 x g for 10 minutes at room temperature. Collect plasma at the top of the tube with at least 0.5 cm distance from the buffy coat. Put 1.0 ml aliquots of plasma into 2 ml cryovials. Store aliquots at -80˚C until shipment.

The Alliance strongly recommends the usage of 2 ml cryovials for storage of plasma specimens. Acceptable cryovials include:

Company name	Catalog number
Nalgene	03-337-7Y (through Fisher)<br>NNI No.: 5012-0020
Fisher brand	05-669-57 (through Fisher)
Corning	03-374-21 (through Fisher)<br>CLS430659 (through Sigma)<br>Corning: 430488
VWR	16001-102

Specimen registration and tracking

USE OF THE ALLIANCE BIOSPECIMEN MANAGEMENT SYSTEM (BioMS) IS MANDATORY AND ALL SPECIMENS MUST BE LOGGED AND SHIPPED VIA THIS SYSTEM.

BioMS is a web-based system for logging and tracking all biospecimens collected on Alliance trials. Authorized individuals may access BioMS at the following URL: http://bioms.allianceforclinicaltrialsinoncology.org using most standard web browsers (Safari, Firefox, Internet Explorer). For information on using the BioMS system, please refer to the 'Help' links on the BioMS web page to access the on-line user manual, FAQs, and training videos. To report technical problems, such as login issues or application errors, please contact: 1-855-55BIOMS. For assistance in using the application or questions or problems related to specific specimen logging, please contact: 1-855-55BIOMS.

After logging collected specimens in BioMS, the system will create a shipping manifest. This shipping manifest must be printed and placed in the shipment container with the specimens.

Shipment of samples

- All samples should be labeled with study number, patient ID number, patient initials, sample collection date and time, and be accompanied by the completed specimen submission shipping manifest which will be generated by BioMS.

  Note for CRA – The following PHI must be removed or blacked out for all specimens or reports: signature, name, date of birth, other identifying information, except initials and study identification number.

- All samples should be shipped to the Alliance Biorepository at Ohio State.
- Specimens may be sent to the Alliance Biorepository on Monday through Thursday for next day delivery. **The Bank cannot receive specimens on Saturdays, Sundays or holidays. Do not send specimens the day before a holiday.**
- The institution is expected to pay the cost of mailing specimens and will be reimbursed through capitation fees set for each individual study.
- Arrange for express courier pick-up through your usual institutional procedure. Ship specimens to the address below:

Alliance Biorepository at Ohio State
Innovation Centre
2001 Polaris Parkway
Columbus, OH 43240
Tel: 614-293-7073
path.calgb@osumc.edu

On the day that specimens are sent to the specimen bank, please contact the bank by phone or e-mail to notify what is being sent and when the shipment is expected to arrive.

7.0 Required Data

Pre-Study Testing Intervals

To be completed within 16 DAYS before registration:

- All blood work
- History and physical

To be completed within 28 DAYS before registration:

- Any X-ray, scan of any type or ultrasound which is utilized for tumor measurement per protocol.

To be completed within 42 DAYS before registration:

- Any baseline exams used for screening,
- Any X-ray, scan of any type or ultrasound of uninvolved organs which is not utilized for tumor measurement.

Patients registering to CALGB 30610 after 1 cycle of therapy must adhere to the "prior to registration" test requirements at the timepoints listed above, with the requirement that the test be done "before first cycle of chemotherapy" rather than "before registration." If the tests and observations, laboratory studies and staging are not obtained within the timeframes specified, prior to the first cycle of therapy, patients will not be eligible to register to CALGB 30610. For patients receiving 1 cycle of non-protocol therapy prior to registering, that first cycle is considered "Cycle 1" for reporting purposes. Once they register and start the chemoRT that will be considered Cycle 2.

Exception: It is acceptable to obtain the following tests prior to Cycle 2 to satisfy eligibility requirements for patients who have received Cycle 1 prior to registration: pulmonary function, LDH, Bili, LDH, AST, Cr, BUN, Mg, Na, Ca.

	Prior to Registration	Day 1 of Each Cycle*	Time of Restaging (q 2 cycles)	Post Treatment Follow up***
**Tests & Observations**				
Physical Examination	X	X		X
Pulse, Blood Pressure	X	X		X
Height	X			
Weight†**/Body Surface Area	X	X		X
Performance Status	X	X		X
Tumor Measurements	X		X	X
Drug Toxicity Assessment		X		X
**Laboratory Studies**				
CBC, Differential, Platelets	X	B		
Bili, LDH, AST, Cr, BUN, Mg, Na, Ca	X	X		
Pulmonary Function (DLCO, FEV-1, FVC)	X			D
Pregnancy test in women of child-bearing potential	X			
**Staging**				
Spiral CT Scan of Chest[A]	X		X	X
Spiral CT Scan of Abdomen[A]	X			
Bone or PET Scan	X			
MRI (or CT) of Brain	X		C	
**Companion (or sub) Studies**[Ψ]				
QOL (70702)	See Section 6.2 for further information.			
Correlative Studies (150712)	See Section 6.3 for further information.			

* Pre-registration labs may be used for day 1 of cycle 1 tests if obtained within 14 days prior to day 1 of Cycle 1. For subsequent cycles labs may be obtained within 48 hours prior to day of treatment. Patients receiving treatment on a Monday may have labs obtained within 72 hours.

** Weight must be recorded weekly during thoracic radiotherapy

*** At least every 3 months for 2 years, then every 6 months for 3 years, then every year for an additional 5 years until disease progression. After progression, follow every 6 months for survival and new malignancy.

† The dose of chemotherapy need not be changed unless the calculated dose changes by ≥ 10%.

A See Section 13.5.3 for CT instructions. Note that a CT scan made as part of the PET-CT is acceptable if it meets all the criteria in Section 13.5.3. Any CT scan that does not meet these requirements must be repeated.

B Obtain on days 1 and 8 of each cycle.

C Obtain MRI or CT of brain prior to initiating prophylactic cranial irradiation.

D Obtain 6 and 12 months from the start of treatment, then as clinically indicated.

Ψ For those patients who consent to participate in the substudies.

8.0 TREATMENT PLAN

Patients registered to CALGB 30610 prior to the start of cycle 1 protocol treatment will start therapy within 7 days of registration.

Questions regarding treatment should be directed to the Alliance Study Chair.

Protocol therapy will consist of 4 cycles of cisplatin and etoposide or carboplatin and etoposide chemotherapy administered every 21 days. Thoracic radiotherapy will begin either on the first day of the first cycle of chemotherapy OR on the first day of the second cycle of chemotherapy.

Patients may be registered to CALGB 30610 following one cycle of chemotherapy. Patients must receive the second cycle of therapy following registration, on day 22-24, so that the patient adheres to a 3-week treatment cycle. Patients not able to be treated within 3 days of day 22 should not be registered to CALGB 30610. For patients registered to CALGB 30610 after 1 cycle of chemotherapy, the thoracic radiotherapy must begin with the second cycle of chemotherapy (the first cycle of protocol chemotherapy after the patient was registered). Additionally, the cycle of chemotherapy given prior to registration will be considered “cycle 1.” Therefore, patients registered following 1 cycle of chemotherapy will receive three cycles of therapy after registration.

The original design was a randomized phase III trial including two experimental treatment arms (70 Gy once daily radiotherapy and 61.2 Gy concomitant boost radiotherapy) and a standard treatment arm (45 Gy twice daily radiotherapy). An interim analysis, conducted after accrual of a pre-determined number of patients, selected one experimental arm based upon a comparison of treatment related toxicity. The most toxic experimental arm, which was Arm C, was discontinued, and the trial now compares standard therapy to the selected experimental regimen.

8.1 Chemotherapy

Patients will be treated with cisplatin and etoposide or carboplatin and etoposide chemotherapy as described below. Standard anti-emetic therapy including steroids such as dexamethasone and a 5-HT3 antagonist is recommended. The use of aprepitant should be considered.

Cisplatin 80 mg/m^2 by IV on day 1 of each 21-day cycle. Prior to each cisplatin treatment prehydrate with at least 1000 ml normal saline and use diuretics per institutional guidelines **OR Carboplatin** AUC 5 by IV on day 1 of each 21-day cycle

Etoposide 100 mg/m^2 by IV on days 1, 2, and 3 of each 21-day cycle.

8.2 Radiation Therapy

Questions regarding Radiation Therapy should be directed to Dr. Bogart, preferably via e-mail.

8.2.1 Required Benchmarks and Pre-approval of 3D Treatment Plans

- CT-based conformal planning is required on this study. In accordance with current guidelines for use of IMRT in clinical trials (see http://www.QARC.org), IMRT may be used only if the degree of tumor motion is assessed and can be limited to 1.0 cm. If required to achieve this goal, techniques for managing or suppressing tumor motion shall be applied.
- Institutions that have been credentialed for participation in RTOG protocol 0617 using 3D conformal techniques will be considered credentialed for use of 3D conformal techniques in this protocol as well. Likewise those institutions credentialed for use of IMRT in RTOG 0617 will be considered credentialed for use of IMRT in this study. A copy of the approval letter from RTOG should be submitted to QARC. Institutions using 3D conformal techniques and not credentialed for RTOG 0617 must complete the 3D Conformal Benchmark. Those treating with IMRT and not credentialed for RTOG 0617 must complete the IMRT Benchmark and IMRT Questionnaire. The benchmark material is available from the Quality Assurance Review Center (www.QARC.org) and must be submitted before patients on this protocol can be evaluated. The IMRT credentialing requirement may also be satisfied by successful irradiation of the RPC's head and neck IMRT phantom. Contact the RPC for information about their phantoms. If techniques are used to compensate for or limit respiratory motion, the QARC questionnaire on respiratory motion management will also be submitted.
- Treatment plans must be reviewed and approved by QARC during the initial week of treatment.
- Equipment
  - **Modality:** Use external beam radiation.
  - **Geometry:** The distance from the radiation source to the prescription point should not be less than 100 cm.
  - **Energy:** Use radiation of megavoltage quality, i.e., X-ray beams with a nominal energy of 4-18 MV. Co-60 treatment is <u>not allowed</u> for treatment on this protocol.
  - **Calibration:** All radiation units used for protocol therapy must have their calibration verified by the RPC.
  - **Field Shaping -** Field shaping shall be done with blocks, which are at least 5 HVL thick, or with multileaf collimation.

8.2.2 Target Dose

- **Prescription Point:** Dose is to be prescribed to an isodose line that encompasses the PTV and that satisfies the dose uniformity criteria in section 8.2.5. The minimum PTV dose must not fall below 95% of the prescription dose. The MTD will be quoted as the PTV minimum target dose. The maximum and minimum point doses (within the PTV) will be reported.
- **Dose Definition:** Dose is to be specified in centigrays (cGy)-to-water.
- **Tissue Heterogeneity:** All radiation doses will be calculated with inhomogeneity corrections that take into account the density differences within the irradiated volume (i.e., air in the lung and bone).

- Prescription Dose and Fractionation:

  Arm A: 45 Gy in 3 weeks

  Patients on Arm A will receive treatment 5 days per week, in twice daily fractions, 1.5 Gy per fraction. The total dose will be 45 Gy in 30 fractions. There are no field reductions on this arm and a single PTV (PTV-1) will be used throughout the entire treatment. All fields must be treated daily and the entire PTV must be treated daily. The treatment plan will limit direct irradiation of the spinal cord during the afternoon treatment for the final 10 days of therapy. Radiation therapy (RT) commences on either day 1 of the first cycle of chemotherapy or day 1 of the 2nd cycle of chemotherapy. There will be a minimum of 6 hours between the morning and afternoon fractions.

  Arm B: 70 Gy in 7 weeks

  Patients on Arm B will receive treatment 5 days per week, in once daily fractions, 2 Gy per fraction. The total dose will be 70 Gy in 35 fractions. There will be a field reduction following 44 Gy. The initial 44 Gy will be directed towards PTV-1 and the subsequent 26 Gy will be directed towards PTV-2. All fields must be treated daily and the entire PTV must be treated daily. Radiation therapy (RT) commences on either day 1 of the first cycle of chemotherapy or day 1 of the 2nd cycle of chemotherapy. A second simulation is allowed to determine PTV-2.

  Arm C: 61.2 Gy in 5 weeks (DISCONTINUED AS OF 03/10/13)

  Patients on Arm C will receive treatment 5 days per week, in once daily fractions during the initial 16 days of treatment and twice daily fractions during the final 9 days of treatment, 1.8 Gy per fraction. The total dose will be 61.2 Gy in 34 fractions. There will be a field reduction (i.e., PTV-2) for the afternoon treatment when twice-daily treatments begin, and the morning treatment during the final 5 days of therapy will also be directed towards PTV-2. Thus, the initial 20 morning treatments will be directed at the PTV-1 and the remaining 14 fractions (9 afternoon treatments and the final 5 morning treatments) will encompass the PTV-2. The treatment plan will limit direct irradiation of the spinal cord during the afternoon treatment when 2 daily treatments are given. All fields must be treated daily and the entire PTV must be treated daily. Radiation therapy (RT) commences on either day 1 of the first cycle of chemotherapy or day 1 of he 2nd cycle of chemotherapy. A second simulation is allowed to determine PTV-2. There will be a minimum of 6 hours between the morning and afternoon fractions when 2 daily treatments are given.

8.2.3 Treatment Technique

- **Conformal (Three Dimensional) Planning** is required on this protocol. Both planar and non-coplanar field arrangements are acceptable. Fields should be chosen to minimize the dose to the normal tissues while encompassing the target volume. The treatment plan used for each patient will be based on an analysis of the volumetric dose including DVH analyses of the PTV and critical normal structures. Each field is to be treated daily.
- **Intensity Modulated Radiation Therapy (IMRT): IMRT is allowed as long as the participating institution is credentialed** for use of IMRT in RTOG 0617 or has completed the IMRT Benchmark and IMRT Questionnaire. The credentialing requirement may also be satisfied by successful irradiation of the RPC's head and neck IMRT phantom. If IMRT is used, the degree of tumor motion shall be assessed and shall be limited to 1.0 cm. If required to achieve this goal, techniques for managing or suppressing tumor motion shall be applied. Acceptable approaches include abdominal

compression, automatic breath-hold (i.e., Elekta ABC device) or a gating approach (e.g., Varian RPM system) or other technologies. Each institution must document both the method used for limiting motion as well as the efficacy of their approach. The motion management questionnaire shall be submitted.

8.2.4 Localization, Simulation, and Immobilization

- A volumetric treatment planning CT study will be required to define gross tumor volume (GTV), clinical target volume (CTV), and planning target volume (PTV)(see definitions below). Each patient will be positioned in an immobilization device in the treatment position on a flat table. Contiguous CT slices, having 3-5 mm thickness through the regions harboring gross tumor and grossly enlarged lymph nodes and 8-10 mm thickness of the remaining regions are to be obtained starting from the level of the cricoid cartilage and extending inferiorly through the entire liver volume. The GTV, CTV, and PTV and normal organs will be outlined on all appropriate CT slices.
- The initial treatment planning CT may be obtained either prior to the first cycle or second cycle of chemotherapy, depending on when thoracic radiotherapy is initiated. If the initial planning CT is obtained after chemotherapy has been initiated, all sites of disease involvement on the prechemotherapy diagnostic CT and /or PET-CT will be designated as GTV. However, treatment planning will take into account changes in tumor size and volume.
- A second volumetric treatment planning CT study is allowed on Arm B (70 Gy QD) and Arm C (DISCONTINUED AS OF 03/10/13) of the study for defining PTV-2. This study should take place approximately one week prior to the initiation of PTV-2 to allow sufficient time for treatment planning.
- Intravenous (IV) contrast during the planning CT is optional provided a diagnostic chest CT was done with contrast to delineate the major blood vessels. If not, IV contrast should be given during the planning CT. If contrast is used, the densities can be over-ridden or the contrast scan must be registered to a non-contrast scan for planning purposes.
- Optimal immobilization is critical for this protocol. Immobilization to assure reproducibility of the set up is necessary.
- The use of four-dimensional radiation treatment planning is highly encouraged. Acceptable methods of accounting for tumor motion include: design of the PTV to cover the excursion of the lung primary cancer and nodes during breathing such as an ITV approach, a maximum intensity projection (MIP) approach, automatic breath-hold (i.e., Eleckta ABC device) or a gating approach (e.g., Varian RPM system).

8.2.5 Treatment Planning/Target Volumes

The nomenclature and definitions of ICRU Reports 50 and 62 shall be followed in this study.

- **Gross Target Volume (GTV)** is the volume occupied by visible or palpable disease. This generally includes sites seen on CT/MRI scan, FDG-PET imaging or biopsy positive sites.
- **Clinical Target Volume (CTV)** is the GTV plus any sites that warrant irradiation because of potential occult tumor involvement.
- **Planning Target Volume (PTV)** is the CTV plus a margin added in order to compensate for variability in treatment setup, breathing, or motion during treatment. PTV is the volume used for treatment planning.

Definition of the GTV: The primary tumor and clinically positive lymph nodes seen either on the pretreatment CT (> 1 cm short axis diameter) or pretreatment PET scan (SUV > 3) will constitute the GTV. This volume(s) may be disjointed. The ITV includes the envelope that encompasses the tumor motion for a complete respiratory cycle. In Arm B and Arm C (DISCONTINUED AS OF 03/10/13) the volume of the GTV may be revised to determine CTV-2 and PTV-2, based on a second CT simulation performed approximately one week prior to the start of the boost field (see Section 8.2.2).

Definition of the CTV:

CTV-1 will include the GTV plus potential occult disease as defined below.

- Potential occult disease:
  a. Ipsilateral hilum (i.e., level 10 lymph node station)

  Elective treatment of the mediastinum and supraclavicular fossae will not be done.

CTV-2 = GTV for the purposes of this protocol. Revised volumes based on a second simulation may be used to determine CTV-2.

Definition of the PTV:

Free-breathing non-ITV approach (i.e., standard CT simulation without 4DCT or fusion of inhalation and exhalation scans):

There are two components to the PTV expansion. The internal motion (IM margin) which should be at least 1 cm in the inferior-superior direction, and 0.5 cm in the axial plane and an additional set-up margin of 0.5 cm. Thus, the PTV-1 includes the CTV-1 plus a total margin of at least 1.5 cm to the superior-inferior dimensions and at least 1.0 cm in the axial plane and the PTV-2 includes the CTV-2 plus a total margin of at least 1.5 cm to the superior-inferior dimensions and at least 1.0 cm in the axial plane.

Breath-hold or gating non-ITV approach:

For breath-hold or gating approaches, the PTV margin should be at least 1 cm in the inferior-superior direction and 0.5 cm in the axial plane. It is expected that daily imaging will be used for both breath-hold and gating techniques.

ITV approach:

If the ITV approach is used, then the PTV margin should account for setup uncertainties and may be individualized but should not be less than 1.0 cm. If daily imaging is used to align the vertebral bodies, then the margins for setup margins may be reduced to 0.5 cm.

For institutions not using 4DCT, the use of fluoroscopy to determine the margin for motion in the inferior superior direction is encouraged.

For institutions with gating technology, the use of respiratory gating is encouraged.

Normal anatomy to be identified: The normal anatomy to be outlined on each CT image will include the lungs (right and left done separately), heart, skin, esophagus and spinal cord. The heart should be contoured from its base to apex, beginning at the CT slice where the ascending aorta originates. The esophagus should be contoured from the bottom of the cricoid to the gastroesophageal junction. The skin and spinal cord should be contoured on each CT slice.

8.2.6 Critical Structures

Normal tissue constraints shall be prioritized in the following order for treatment planning: 1=spinal cord, 2=lungs, 3=esophagus, 4= heart

- Spinal Cord:

Arm A

The treatment plan will avoid (> 20%) direct irradiation of the spinal cord during the afternoon treatment for the final 10 days of therapy. The spinal cord dose limitation is the highest priority dose constraint and thus must be met irrespective of other constraints. Total “direct” plus “scatter” dose to the spinal cord must not exceed 41 Gy.

Arm B

The spinal cord dose limitation is the highest priority dose constraint and thus must be met irrespective of other constraints. Total “direct” plus “scatter” dose to the spinal cord must not exceed 50.5 Gy.

Arm C (DISCONTINUED EFFECTIVE 03/10/13)

The treatment plan will avoid (> 20%) direct irradiation of the spinal cord during the afternoon treatment for the final 9 days of therapy such that the spinal cord is not directly treated twice daily. The spinal cord dose limitation is the highest priority dose constraint and thus must be met irrespective of other constraints. Total “direct” plus “scatter” dose to the spinal cord must not exceed 50.5 Gy.

- **Lungs:** The dose-volume constraint to the lungs is the second highest priority and must be met, except if it conflicts with the cord dose constraints. The volume of both lungs that receive more than 20 Gy (the V_{20}) should not exceed 40 % of the total. Alternatively, the mean lung dose should optimally be ≤ 20 Gy. (By total lung volume we mean the total lung minus the CTV.)

If either of these constraints is exceeded, several solutions can be entertained.

- First, one might increase the weighting of AP / PA treatments by one and reduce the obliques. This can be done as long as the cord dose (above), which takes precedence, is not exceeded.
- Second, one can reduce the CTV to the minimum range suggested above.
- Third, one can try to reduce the PTV by using respiratory gating techniques.
- If after all attempts to decrease the V_{20} to below 40%, the V_{20} value still exceeds this limit, the patient should still be treated to the dose and fractionation on the arm to which they were randomized.
- **Esophagus:** The mean dose to the esophagus is optimally kept below 34 Gy. This is not an absolute requirement, but is strongly recommended unless other, more critical constraints force the situation.
- **Heart:** The following limits are recommended: 60 Gy to <1/3, 45 Gy to <2/3, and 40 Gy to <100% of the heart.

8.2.7 Definitions of Deviations in Protocol Performance

Prescription Dose

- **No Deviation:** ≥ 99% of the PTV receives ≥ 93% of the prescribed dose, and a contiguous volume of no more than 2cc inside PTV exceeds 20% of the prescribed dose.
- **Minor Deviation:** Deviations of this magnitude are not desirable, but are acceptable. Coverage that is equal to 93% of the prescribed dose and falls between 99% and 95% of the PTV, or a contiguous volume of no more than 2cc inside the PTV exceeds 20-25% of the prescribed dose.
- **Major Deviation:** Doses in this region are not acceptable. More than 1 cm^3 of tissue outside the PTV receives ≥ 120% of the prescribed dose, or 93% of the prescribed dose

falls below 95% of the PTV, or a contiguous volume of no more than 2cc inside the PTV exceeds 25% of the prescribed dose.

Volume

- **Minor Deviation:** Margins less than specified, or field(s) 1-3 cm greater than specified.
- **Major Deviation:** Fields transect tumor or specified target volume(s), or fields are more than 3 cm greater than specified.

Critical Organ

- **Major Deviation:** The maximum dose to the spinal cord exceeds the limits in section 8.2.6.

8.2.8 Quality Assurance Documentation:

Digital Submission:

Submission of treatment plans in digital format (either Dicom RT or RTOG format) is encouraged. Instructions for data submission are on the QARC Web site at www.qarc.org. Any items on the list below that are not part of the digital submission should be submitted in hard copy form.

Note: Black and white copies of color data may be submitted, provided lines are clearly labeled and the copy is legible. Otherwise, only color copies will be accepted.

- Within three days of the start of radiotherapy, the following data shall be submitted for on-treatment review.

  a. Copies of all diagnostic materials (including diagnostic CT) and surgical reports.
  b. Copies of treatment planning CT used in defining the target volumes.
  c. Copies of simulator films and /or digitally reconstructed radiographs (DRRs) for each field. The GTV, CTV, PTV should be drawn on the simulator films.
  d. Copies of verification (portal) films (or hard copy of real time portal images) for each field.
  e. Copies of worksheets and/or printouts used for calculations of monitor units.
  f. Color hard copy isodose distribution for the total composite dose plan in the axial, sagittal, and coronal planes, which includes the isocenter of the planning target volume.
  g. RT-1 or IMRT Dosimetry Summary Form.
  h. One set of orthogonal anterior/posterior and lateral films for isocenter localization for each group of concurrently treated beams. If portals being submitted contain an orthogonal set, this is sufficient.
  i. Beam's Eye Views (BEVs) for all fields and showing the PTV and critical structures. BEV hard copies must be in color to enable reviewers to identify structures.
  j. Dose volume histograms for the total treatment for the target volumes, lungs, heart, and spinal cord. If IMRT is used, a DVH shall also be submitted for a category of tissue called “unspecified tissue,” which is defined as tissue contained within the skin, but which is not otherwise identified by containment within any other structure.
  k. Documentation of an independent check of the calculated dose if IMRT is used.
  l. If IMRT is used for tumors in the thoracic region, a description of the steps taken to suppress/manage motion to achieve appropriate simulation.

- Within one week of the completion of radiotherapy, the following data shall be submitted.
    - Copies of additional simulation films and verification (portal) films for any field modifications made subsequent to the initial reporting of data for on-treatment review.
    - A "RT-1 or IMRT Dosimetry Summary" form if changes have been made subsequent to submission of on-treatment data.
    - The "RT-2 Radiotherapy Total Dose Record" form.
    - A copy of the patient's radiotherapy record including the prescription, and daily and cumulative doses to all required areas and reference points.
    - Copies of calculations performed subsequent to the submission of the on-treatment data.
    - Copies of isodoses.
    - All data should be forwarded to:

      T.J. Fitzgerald, M.D.
      Quality Assurance Review Center
      640 George Washington Hwy, Suite 201
      Lincoln, RI 02865
      Tel: (401) 753-7600
      Fax: (401) 753-7601
    - Questions regarding the completion of RT-1 and RT-2 data forms, dose calculations or documentation should be directed to:

      Alliance Protocol Dosimetrist
      Quality Assurance Review Center
      640 George Washington Hwy, Suite 201
      Lincoln, RI 02865
      Tel: (401) 753-7600
      Fax: (401) 753-7601
    - Questions regarding the radiotherapy section of this protocol, including treatment interruptions, should be directed to:

      Jeffrey A. Bogart, M.D.
      Upstate Medical University Center
      750 E. Adams Street
      Syracuse, NY 13210
      Tel: (315) 464-5276 Fax: (315) 464-5943
      bogartj@upstate.edu

      OR

      Ritsuko Komaki, M.D.
      MD Anderson Cancer Center
      1515 Holcombe Blvd., Unit 97
      Houston, TX 77030
      Tel: (713) 563-2328 Fax: (713) 563-2331
      rkomaki@mdanderson.org

8.2.9 Prophylactic Cranial Irradiation

- Prophylactic cranial irradiation (PCI) should be offered to all patients with a complete tumor response (CR) or near complete response (very good PR) with only residual chest abnormalities of indeterminate nature following completion of combined modality therapy. This will be determined based on the re-staging studies obtained following all four cycles of chemotherapy and thoracic irradiation. PCI should start approximately 3-6 weeks following the final cycle of chemotherapy. No concurrent chemotherapy is given with PCI. For patients with a response to therapy that is less than CR or very good PR, the use of PCI is at the investigator's discretion.
- Treatment should be delivered with megavoltage radiation.
- The dose and fractionation scheme for PCI is 2500 cGy given in 250 cGy fractions. An RT-2 Form must be completed indicating the start and end date of treatment, fraction dose, number of fractions and total dose. This form should be mailed or faxed to QARC at the address in section 8.2.8.
- The treatment field should encompass the entire cranial contents. The base of the field will extend from the supraorbital ridge, the lateral canthus of the orbit, through the tip of the mastoid process, which is 1.5-2 cm below the external auditory meatus, back to the C1-C2 vertebral interspace.

9.0 DOSE MODIFICATIONS AND MANAGEMENT OF TOXICITY

If different percentages of dose reductions for a given drug are required because of two different types of toxicities, the greater percentage dose reduction should be employed.

9.1 Hematologic Toxicity

Granulocyte or Platelet Counts for Day 1: Based on counts within 2 days of the start of each cycle, give the following:

Granulocytes/µl		Platelets/µl	Cisplatin, Carboplatin and Etoposide
≥ 1,500	and	≥ 100,000	100%
< 1,500	and/or	< 100,000	0*

* Hold protocol therapy; repeat counts twice weekly and reinstitute therapy at 100% when granulocytes ≥ 1,500/microliter and platelets ≥ 100,000/microliter. If counts do not reach these levels within 3 weeks of the next scheduled treatment, discontinue all protocol therapy.

Neutropenia or Febrile Neutropenia:

For nadir neutropenia in the absence of fever or with fever that is successfully treated by oral antibiotics, there will be no dose adjustment. Filgrastim, sargramostim, or pegfilgrastim are allowed for patients with neutropenia that delays day 1 chemotherapy by one week or more for cycles 3 and 4 (after the completion of radiation therapy). **WBC growth factors may <u>not</u> be used during radiation therapy.** Filgrastim, sargramostim, or pegfilgrastim may then be given after chemotherapy to prevent future treatment delays in subsequent cycles at the discretion of the treating physician (after the completion of radiation therapy). Any use of colony stimulating factors should be documented on the Remarks Addenda Form (C-260).

For chemotherapy delays of more than 7 days on any subsequent cycle of treatment, both chemotherapy drugs should be dose-reduced by 25% for all subsequent cycles of chemotherapy.

For neutropenic fever (ANC ≤ 500/microliter and temperature ≥ 100.5) requiring intravenous antibiotics, the doses of all chemotherapy drugs should be reduced by 25% for the next cycle and all subsequent cycles.

For grade 4 nadir thrombocytopenia (platelets ≤ 25,000), the dose of all chemotherapy drugs should be reduced by 25% from the previous dose for the next cycle and for all the subsequent cycles of chemotherapy.

9.2 Gastrointestinal Toxicity

Nausea and Vomiting

All patients should receive antiemetics to prevent nausea and vomiting. Specific antiemetic therapy is left to the discretion of the physician treating the patient (steroids and 5-HT3 antagonists should be used). If vomiting is severe, consider hospital admission and/or use of aprepitant if possible. Do not modify dose.

9.3 Hepatic Toxicity

Bilirubin	Etoposide
<1.5 x ULN	100%
1.5-3.0 x ULN	50%
>3.0 x ULN	30%

9.4 Nephrotoxicity

(based on measured or calculated creatinine clearance)

Creatinine Clearance (ml/min)	Cisplatin
≥ 70	100%
70-50	67%
< 50	0*

* If serum creatinine clearance is < 50 ml/min on day 1 of the next cycle, delay the start of that cycle for up to 2 weeks (check creatinine at least weekly). If CrCl decrease persists beyond 2 weeks, omit cisplatin for that cycle. If CrCl does not recover to ≥ 50 ml/min after 3 weeks, remove patient from protocol therapy.

9.5 Hypomagnesemia

Hypomagnesemia is not an indication for stopping therapy. Oral or parenteral magnesium supplementation is indicated for serum magnesium levels ≤ 1.5 mEq/l.

9.6 Neurologic Toxicity

Grade	Cisplatin/Carboplatin
0-1	100%
2	75%*
3	0**

* Patients with grade 2 neurotoxicity should recover to grade 1 or better prior to retreatment with this (75%) dose reduction. If grade 2 neurotoxicity recurs with 75%, drug will be given at 50% upon resolution of neurotoxicity to grade 0-1. If grade 2 neurotoxicity persists for 3 weeks, remove the patient from protocol therapy (see section 14.2).

** Discontinue drug for neurotoxicity ≥ grade 3.

9.7 Ototoxicity

Remove patient from therapy for ≥ grade 3 ototoxicity.

9.8 Allergic Reactions

Discontinue treatment promptly if ≥ grade 3 anaphylaxis develops.

9.9 Grade 3/4 Non-Hematologic Toxicity

If a patient develops grade 3 or 4 non-hematologic toxicity not detailed above (excluding anorexia, fatigue, fever without grade 3/4 neutropenia, and alopecia), hold all therapy. Therapy can be restarted if the toxicity has resolved to ≤ grade 1 by the time of the next treatment. Doses of all chemotherapy should then be reduced by 25%. If therapy is held for more than 3 weeks, remove the patient from protocol therapy.

9.10 Radiotherapy Dose Modifications for In-field Non-Hematologic Toxicities

Radiation treatment will be interrupted for grade 4 in-field toxicity and/or grade 4 neutropenia with fever.

Aggressive supportive care is encouraged throughout the course of radiotherapy. If the patient is near completion of therapy, then every attempt should be made to complete treatment despite acute toxicity. Otherwise, treatment should be restarted when the accompanying toxicity declines to ≤ grade 2.

If treatment is interrupted for more than 3 weeks due to non-hematologic toxicity, remove the patient from protocol treatment.

Use the following treatment modification table for in-field non-hematologic toxicity:

In-field	CTCAE Toxicity Grade	XRT	Cisplatin/Carboplatin	Etoposide
Esophagus/pharynx (on day of treatment)	4	Hold treatment until ≤ grade 2	Hold treatment until ≤ grade 2	Hold treatment until ≤ grade 2
Esophagus/pharynx (on day of chemo)	3	No change	Hold treatment until ≤ grade 2	Hold treatment until ≤ grade 2
Esophagus/pharynx (on day of chemo)	2	No change	No change	No change
Pulmonary	4	Discontinue	Hold treatment until ≤ grade 2	Hold treatment until ≤ grade 2
Pulmonary	3	Hold treatment until ≤ grade 2	Hold treatment until ≤ grade 2	Hold treatment until ≤ grade 2
Skin	4	Hold treatment until ≤ grade 2	Hold treatment until ≤ grade 2	Hold treatment until ≤ grade 2
Skin	3	No change	No change	No change

9.10.1 For grade 4 infield esophagitis, radiotherapy and chemotherapy should be interrupted as detailed in the table above. Re-evaluate patient weekly.

9.10.2 For grade ≥ 3 esophagitis/pharyngitis, dermatitis, or other in-field radiotherapy- related toxicity, on day of chemotherapy administration during any treatment week, omit cisplatin/carboplatin and etoposide until toxicity resolves to grade ≤ 2 as detailed in the table above.

9.10.3 Radiotherapy should be interrupted only for grade 4 in-field toxicity and resumed when that toxicity has decreased to grade ≤ 2 as detailed in the table above. If the patient experiences esophagitis so that IV fluid support is needed, insertion of a feeding tube should be considered.

9.10.4 For grade 3 esophagitis, radiotherapy can be continued with pain management and IV support.

9.11 Dose Modifications for Obese Patients

There is no clearly documented adverse impact of treatment of obese patients when dosing is performed according to actual body weight. Therefore, **all dosing is to be determined solely by (1) the patient's BSA as calculated from actual weight or (2) actual weight without any modification unless explicitly described in the protocol**. This will eliminate the risk of calculation error and the possible introduction of variability in dose administration. **Failure to use actual body weight in the calculation of drug dosages will be considered a major protocol deviation.** Physicians who are uncomfortable with administering chemotherapy dose based on actual body weight should not enroll obese patients on Alliance protocols.

10.0 COMPANION STUDIES

There are two substudies within CALGB 30610. Patients who receive one cycle of chemotherapy prior to registering on CALGB 30610 will NOT be offered participation in either companion study.

The correlative science and the quality of life studies must be offered to all patients enrolled on CALGB 30610 (although patients may opt to not participate). These substudies do not require separate IRB approval. The substudies included within CALGB 30610 are:

- CALGB 150712: Correlative Science Studies in CALGB 30610
- CALGB 70702: Quality of Life Studies in CALGB 30610, **closed to accrual as of 8/30/17**

10.1 Correlative Science Substudies (CALGB 150712)

10.1.1 Exploratory Investigation of Circulating Biomarkers

The goal of the first correlative study is to use the multiplex bead assay to systematically examine potential blood-based biomarkers of anti-tumor response and therapeutic resistance to the two chemoradiotherapy regimens, with a focus on circulating cytokines and angiogenic factors.

This correlative study will be performed at MD Anderson Cancer Center in the laboratory of Dr. John Heymach, M.D., Ph.D.

Rationale: Previous studies have established that both the tumor response to radiotherapy and radiation-induced gastrointestinal toxicity are dependent on the induction of tumor endothelial apoptosis and angiogenesis [27, 28]. Furthermore, radiation induces compensatory pathways, including hypoxia inducible factor-1 (HIF-1), which affect endothelial survival and tumor radiosensitivity through mediators such as VEGF and basic

FGF [29]. Interestingly, the sequencing and frequency of radiotherapy had a significant impact on the activation of these pathways [29]. Assessing the treatment-induced changes in circulating angiogenic factors and cytokine expression, particularly HIF-regulated factors, may therefore provide important information regarding the tumor response to different treatment regimens. In this study we will investigate a biomarker profile consisting of circulating potential blood-based biomarkers of response and resistance, including proangiogenic cytokines (i.e., EGF, bFGF, IL-8) and markers of endothelial damage (soluble E-selectin, soluble VEGFR-2).

Objectives: The exploratory objectives of the correlative study are:

1. To determine whether the baseline biomarker profile, or changes in the profile during treatment, correlate with clinical outcome as judged by objective response rate, progression free survival, and overall survival.
2. To compare the changes in the biomarker profile between the two treatment arms.

Hypotheses

1. Higher-dose, accelerated radiation therapy will cause a larger rise in circulating markers of endothelial damage than hyperfractionated and accelerated radiotherapy (HFXART) due to a greater induction of tumor endothelial apoptosis.
2. Other circulating biomarkers may be useful for helping to identify patients most likely to benefit from therapy, monitor antitumor responses, and understand mechanisms of therapeutic resistance.

Method for Multiplex Bead Assay

Plasma will be used for the assay. We will use the multiplexed bead suspension arrays for 33 C/AFs (Biosource™ Human Cytokine 30-Plex for Luminex™ panel, Biosource, Camarillo, CA; Lincoplex™ Human Cardiovascular Disease I kit, LINCO Research/Millipore, St. Charles, MO). The Luminex Multiplex Cytometric Bead Array (Multiplex) assay is ideal for exploratory investigations using statistical support. The assay has many advantages. It requires as little as 50 µl of plasma, rather than the 100-200 µl that is required for the routine ELISA procedures. In addition, the Multiplex has an advantage over the ELISA in its ability to measure up to 25-33 cytokines in one run using a single sample, thereby providing relative concentrations among 25-33 cytokines and also minimizing the variance of the data.

10.1.2 Biomarkers Specific for Small Cell Lung Cancer

The second correlative study will propose correlative translational studies to investigate known promising circulating markers in the plasma in small cell lung cancer. These studies will utilize the standard ELISA assay, and will be performed at the University of Rochester Cancer Center in the laboratory of Dr. Yuhchyau Chen, M.D., Ph.D.

Rationale: Several serum/plasma markers have been found promising in the prognostic value for small cell lung cancer. These include neuron-specific enolase (NSE), carcinoembryonic antigen (CEA), BB isoenzyme of creatine kinase (CK-BB), angiogenic factors Interleukin 8 (IL-8) and Vascular Endothelial Growth Factor (VEGF). There are also new markers worth investigation, which include the stem cell factor (SCF), granulocyte-macrophage-colony stimulating factor (GM-CSF), and tumor hypoxia marker osteopontin. Background information of these serum/plasma biomarkers is briefly summarized as follows:

<u>Carcinoembryonic antigen (CEA)</u>: A number of reports suggested that CEA elevations are more prevalent in SCLC patients with extensive disease (40%-65%) than limited stage (0-

38%) [30, 31]. Some reports suggested a prognostic value for monitoring response to chemotherapy and predicting disease recurrence [32].

Neuron-specific enolase (NSE): SCLC is a neoplasm with neuroendocrine origin. Serum NSE is thought to be the most sensitive tumor marker of SCLC at the time of diagnosis. While one study reported a correlation of NSE with disease extent and disease outcome [33], many other reports confirmed that NSE was a strong independent prognostic factor for complete response, remission duration and overall survival [34, 35]. In a multivariate analysis, NSE, followed by performance status and serum albumin, was the best independent predictor for survival [36].

BB isoenzyme of creatine kinase (CK-BB): CK-BB is also a neuroendocrine marker. It was found to have the best correlation with regard to predicting response and survival when compared with NSE and CEA in the same cohort of patients [33].

Interleukin-8 (IL-8): Tumor angiogenesis is one of the most important biologic features related to tumor growth and metastasis. IL-8 is a cytokine marker with angiogenic property and has been found in at least one study that the serum level was significantly increased in SCLC patients compared with healthy controls [37].

Vascular Endothelial Growth Factor (VEGF): VEGF is considered the most potent angiogenic factor in promoting growth of tumor vasculature. Tumor expression of VEGF as well as circulating VEGF have been demonstrated to be of prognostic significance, correlating with tumor progression or patient survival [38-40]. One study showed that high pretreatment serum VEGF level was an adverse prognostic factor for survival independent of tumor stage [40], other studies found a correlation between serum VEGF level and stage progression [41].

Stem cell factor (SCF): SCF, the ligand for c-Kit receptor, has been implicated in the regulation of angiogenesis in hematopoietic and nonhematopoietic malignancies. At least 70% of SCLC tumor specimens and cell lines co-express SCF and c-Kit [42]. SCF/c-Kit pathway is functional in an autocrine or paracrine fashion in SCLC [43-46]. One study found that patients with SCLC have significantly increased levels of SCF when compared with the control [47].

Granulocyte-macrophage-colony stimulating factor (GM-CSF): GM-CSF is a known cytokine for the proliferation of hematopoietic cells. At least one study found significantly increased levels of GM-CSF in patients with SCLC when compared with the control [47].

Osteopontin (OPN): Tumor hypoxia has been shown to affect the malignant progression of tumors, as well as being one of the leading causes of cancer resistance to chemotherapy and/or radiotherapy. Studies have indicated that hypoxia increases tumor invasiveness and dissemination in human solid tumors [48, 49]. Hypoxia also regulates VEGF expression by enhancing transcription of the VEGF gene and by stabilizing its mRNA. It is now clear that the transcription factor hypoxia-inducible factor (HIF) 1a is the major regulator of VEGF transcription in response to hypoxia [50, 51]. Molecular laboratory work has suggested that OPN may be linked to tumor hypoxia and plasma OPN level was increased in patients with hypoxic tumors of head and neck, and the levels correlated with clinical outcomes [52].

In addition to analyzing the small cell lung cancer specific biomarkers as described above, we will also include two additional groups of proteins. First, we will assess factors that we have already established to be modulated by radiotherapy or endothelial damage, including VEGF, soluble VEGFR-2, and E-selectin [53-55]. The second set of factors will be proangiogenic cytokines that may contribute to resistance by promoting the proliferation and survival of tumor endothelial cells. First, several selected proangiogenic cytokines

known to play a role in angiogenesis in lung cancer, including VEGF, basic fibroblast growth factor (bFGF), transforming growth factor α (TGF-α), and interleukin-8 (IL-8).

Objectives: The objectives of the correlative studies for small cell specific circulating markers are:

1. To investigate the value of the published markers specific to small cell lung cancer in the large phase III clinical study setting. We will analyze the baseline biomarker profile, changes in the profile during treatment at the time of recurrence to correlate with the response rate, progression free survival, and overall survival of these patients.
2. To compare the differences in the biomarker profile between the two treatment arms.

Hypotheses

1. Circulating biomarkers may have prognostic or predictive value of small cell lung cancer.
2. The differences in circulating biomarkers between the two treatment arms may reveal information correlating with efficacy of treatments.

Method for the ELISA Assay

ELISA is a standard laboratory analytical method for protein quantification in the plasma or serum (R&D Systems, Minneapolis, MN). This assay requires a significant volume of sample (100-200 µl) for each biomarker. The ELISA assay has high sensitivity and high precision, and thus has been the standard assay of cytokine measurement.

10.1.3 ProGRP as a Marker to Monitor the Response to Therapy and for the Prediction of Relapse Following Therapy

The goal of this correlative study is to evaluate the performance of proGRP as a biomarker in SCLC lung cancer. In addition, this study will provide an opportunity to better understand the role proGRP to monitor the response to therapy and the relapse following therapy.

This correlative study will be performed at an Abbott Center of Excellence, which are clinical laboratories chosen for their expertise in assay evaluation and biomarker evaluation. The target Center of Excellence for this study is the clinical laboratory at Johns Hopkins run by Drs. Daniel Chan and Lori Sokoll. This is a CLIA approved laboratory.

Rationale: Gastrin releasing peptide (GRP) is a gut hormone and is widely distributed in the mammalian gastrointestinal tract, nervous system and pulmonary tract. GRP is an autocrine growth factor, and binding of GRP to cell surface receptors leads to altered expression of the c-fos oncogene and cell proliferation.

Tumor tissues from SCLC patients show amplification of GRP mRNA and protein. However, serum GRP was not useful for clinical diagnosis due to its very poor stability in specimens. In 1994, an assay to detect a ProGRP fragment (residues 31-98) in serum was developed because the molecule was more stable than GRP. Approximately two thirds of patients with SCLC have elevated concentrations of ProGRP in their serum. The clinical usefulness of the ProGRP 31-98 measurements has been widely reported for the diagnosis, the prediction of prognosis, and the monitoring of treatment in patients with SCLC (94). Neuron-specific enolase (NSE) has been also used as a tumor marker of SCLC, however compared to NSE, ProGRP has relatively high sensitivity, specificity and early recognition of relapse in patients with SCLC (95). The progression of SCLC in patients under therapy in small cohorts of 44 patients and 66 patients has been reported. The majority of patients under treatment showed decreased levels of ProGRP, particularly those with objective responses. The sensitivity for detection of responses ranged from 70-79.7% and the addition

of NSE was found to improve sensitivity to 85.9%. The trend of ProGRP in patients with progressive disease was generally upward.

There have been many publications over the years showing the clinical utility of proGRP as a marker for SCLC. Some important reviews of the literature (96-98) have demonstrated the proGRP was the best tumor marker in his evaluation for patients with SCLC. In addition, the National Academy of Clinical Biochemists Practice Guidelines and Recommendations for the Use of Tumor Markers in Lung cancer recognize the clinical use of proGRP in the differential diagnois of lung masses and in the monitoring of therapy response and detection of recurrent disease (Stieber et al, manuscript in review, Clin Chem.) From the multiple reports, it is clear that proGRP is a valuable tumor marker for SCLC, but it has not been validated in a prospective study.

Objectives: The objectives of this correlative study are:

Primary:

1) To evaluate the correspondence between increases in plasma ProGRP concentrations and disease progression/recurrence.

Secondary:

1) To evaluate the potential for plasma ProGRP concentrations at baseline, after each cycle of chemotherapy and at first evaluation following completion of chemotherapy to predict PFS and OS.
2) To evaluate the correspondence between longitudinal decreases in plasma ProGRP concentrations and clinical response.

Exploratory:

1) To evaluate novel biomarkers for their ability to improve correspondence and prediction of response to therapy in patients with SCLC. This may involve the use of multiple biomarkers, including ProGRP, in conjunction with the biomarkers specific for small cell lung cancer being studied by Dr. Yuhchyau Chen.

Hypotheses

1) ProGRP will have a relatively high sensitivity, specificity and early recognition of relapse in patients with SCLC. An increase in proGRP level may be detected approximately a month prior to clinical evidence of relapse
2) The majority of patients under treatment will show decreased levels of ProGRP, particularly those with objective responses. NSE and proGRP together may be a combination of biomarkers that most accurately predicts response to therapy.

Methods

Plasma is the sample type that will be used for the assay. The plasma levels of ProGRP will be assayed on the ARCHITECT ProGRP assay. The ARCHITECT ProGRP assay is a automated two-step, sandwich format. Analyte capture is with paramagnetic microparticles coated with two mouse monoclonal anti-ProGRP 31-98 antibodies: 3G2 (amino acids of ProGRP 84-88) and 2B10 (amino acids of 71-75), which can capture the C-terminal side of ProGRP 31-98. Detection of the ProGRP analyte microparticle complex is by an acridinium labeled mouse monoclonal antibody conjugate. The mouse monoclonal antibody of 3D6-2 can capture the N-terminal side of the protein (amino acids of ProGRP 40-60). Exposing the reaction mixture to on-board trigger reagents containing peroxide at alkaline pH causes the release of light that is proportional to the ProGRP level in the specimens, calibrators, or controls. The ARCHITECT ProGRP assay calibrators range from 0 to 5 000 pg/mL (0, 20, 80, 320, 1 250, and 5 000 pg/mL). A 1:10 autodilution is used to extend the measurable

range up to 50 000 pg/mL. ARCHITECT ProGRP control concentrations are 40, 160, and 2 500 pg/mL. To minimize the risk of interference from human-anti-mouse antibodies, heterophilic antibodies, and rheumatoid factor, blocking agents and murine mAbs of different isotypes are used in the assay. Furthermore, the F(ab')2 portion of the antibody with Fc portion eliminated by pepsin digestion is used to produce the conjugate. The assay is fully automated and measurement is completed within 30 minutes with 200 tests / hour throughput.

The ARCHITECT ProGRP assay is available CE Marked for sale in Europe and approved for sale in Japan and has been fully validated. The performance was defined by multisite studies in Japan, Germany and the US. These results are in press (Clinical Chemistry and Laboratory Medicine) and is summarized as follows: The total precision %CV for 9 analyte levels was between 2.9-5.7. The analytical sensitivity of the assay was between 0.20-0.88 pg/mL. The functional sensitivity at 20%CV was between 0.66-1.73 pg/mL. The assay was linear up to 50 000 pg/mL with a 1:10 autodilution protocol. The calibration curve was stable for 30 days. The comparison with Fujirebio microtiter plate EIA ProGRP assay gave a slope of 0.93 and a correlation coefficient (r) of 0.99. No interference was seen with chemotherapeutic agents or common interfering substances (bilirubin, hemoglobin, triglycerides, high protein, etc.).

The goal of this correlative study is to obtain information on the clinical value of ProGRP in the US to support an FDA submission and to provide information on its clinical utility to the medical professionals responsible for treating patients with SCLC.

10.2 Health Related Quality of Life (CALGB 70702)

As early as 1956, the World Health Organization defined quality of life as "a complete physical, mental and social well-being and not merely the absence of disease or infirmity" [56]. Thirty-four years later, the definition had not changed, with the major areas of quality of life consisting of physical/occupational status, psychological state, social interaction and somatic sensation [57]. These areas may be the most likely to be impacted by different cancer treatment regimens, with disease factors and treatment toxicity affecting physical symptoms and functioning, and consequently, psychological state and social functioning.

In this dose-response thoracic radiotherapy trial in limited small cell lung cancer, three different doses are being compared in terms of their response rates, failure-free survival and toxicity: 70 Gy (2 Gy once-daily for 7 weeks), 61.2 Gy (1.8 Gy once-daily for 16 days followed by 1.8 Gy twice daily for 9 days), and 45 Gy (1.5 Gy twice-daily over 3 weeks). In Turrisi's study [5], the major increased toxicity for the hyperfractionated dose of 45 Gy in 3 weeks was a doubling of the severe (grades 3 and 4) acute esophagitis rate. Esophagitis typically limits oral intake, requires a change in diet (soft-liquid), may necessitate frequent IV hydration and may be associated with significant fatigue, decreased activity and functioning (and possibly not be able to work) and pain. The impact of these side effects on patient's psychological state and potentially social interaction for those with esophagitis can be significant. In Mehta et al.'s [58] phase II trial, 30 non-small cell lung cancer patients were administered hyperfractionated accelerated radiotherapy: 54 Gy over 12 consecutive treatment days, with three fractions administered daily with a 6 hour interval between fractions. Of the 28 patients who completed treatment, 22% had grade 3 and 4 esophagitis [58]. All esophagitis resolved after treatment. However, in Auchter et al.'s [59] quality of life study of these patients, there were statistically significant declines in physical and functional well-being at treatment completion, with a return to baseline levels occurring at 4 weeks post-treatment completion. Emotional state improved by the time of treatment completion and at 4 weeks post-treatment completion. In Bonner et al.'s [60] phase III trial involving limited stage small-cell lung cancer, while there were no differences

in overall progression rates or overall survival between the once-daily thoracic radiation (50.4 Gy) and twice-daily (48 Gy twice-daily with a two week break after 24 Gy) dose groups, those who received the twice-daily dose had significantly greater esophagitis (grade 3 or greater) than those receiving the once-daily dose. However, there were no significant differences in quality-adjusted life years between patients receiving once-daily versus twice-daily thoracic radiation [61]. In a study by Bailey et al. [62], non-small cell lung cancer patients were randomized to continuous hyperfractionated accelerated radiotherapy (54 Gy, 1.5 Gy three times daily for 12 days) or conventional radiotherapy (60 Gy: 2 Gy in 30 fractions for 6 weeks). Apart from patients in the hyperfractionated accelerated group reporting transient pain on swallowing and heartburn, there was little difference between the two arms of the trial. To date, there is no prospective, longitudinal data on the impact of hyperfractionated accelerated dose at the level proposed in this study on patients' quality of life.

10.2.1 Objective: To compare patients' quality of life between these regimens in terms of their physical symptoms, physical functioning and psychological state

Hypothesis A: Patients in the hyperfractionated accelerated dose (45 Gy) group will experience worse lung cancer specific physical symptoms and physical functioning, as measured by the FACT TOI (Trial Outcome Index)-Lung Cancer and FACT-Esophageal Cancer Eating and Swallowing Indices, than those in the high dose daily (70 Gy) and concomitant boost radiotherapy (61.2 Gy) groups when all RT has been completed in each arm of the study at 12 weeks after the start of radiation therapy. This hypothesis will be revised to reflect the change in the research design due to the elimination of one of the experimental arms (high dose daily or the concomitant boost radiotherapy arm), due to greater toxicity.

Hypothesis B: Patients in the hyperfractionated accelerated dose (45 Gy) group will experience worse esophagitis (measured by the ECOG Acute Esophagitis Scale and difficulty swallowing item), and as a consequence worse psychological state, (measured by the Hospital Anxiety and Depression Scale), and lower quality-adjusted life years (measured by the EQ-5D) than those in the high dose daily (70 Gy) and concomitant boost radiotherapy (61.2 Gy) groups when all RT has been completed in each arm of the study at 12 weeks after the start of radiation therapy. This hypothesis will be revised to reflect the change in the research design due to the elimination of one of the experimental arms (high dose daily or the concomitant boost radiotherapy arm), due to greater toxicity.

Hypothesis C: Patients in the high dose daily 70 Gy arm of the study will experience greater treatment inconvenience (measured by a single item developed for this study), than those in the other two arms of the study, due to having to come into clinic for 7 weeks, the longest duration of the three arms. If the 70 Gy arm is eliminated due to greater treatment toxicity, than this hypothesis will be revised to reflect that those in the 61.2 Gy arm will experience greater treatment inconvenience than those in the 45 Gy arm, due to having to come into clinic for 5 weeks.

10.2.2 Methods

English-speaking patients who consent will be given a packet of quality of life questionnaires to be completed in the clinic. The CRA will be available to answer any questions that the patients have, and review the questionnaires for completeness when handed in. If the questionnaires are not complete, patients will be asked if they left out answering the question by mistake or because they didn't wish to answer the question. If the former, patients will be asked to answer those questions; if the latter, patients will not be asked anything further. Patients will be assessed at baseline, 3, 5, 7, 12, 26, and 52 weeks

after the start of radiation therapy (see schedule outlined below). By 7 weeks, RT will have been completed for all arms of the study. The 12, 26, and 52 week assessments will allow us to examine the longer term effects of toxicity on patients' quality of life. The CRAs at the institutitions will be called by the centralized CRA at the Psycho-Oncology Research Office at Dana-Farber Cancer Institute at each timepoint to remind them to administer the questionnaires to the patients. The schedule of assessments is outlined below:

a) Baseline

b) 3 weeks after the start of radiation therapy (completion of RT on 45 Gy, 1.5 Gy twice daily over 3 weeks. Mid-RT for 70 Gy once daily over 7 weeks; and 61.2 Gy, 1.8 Gy daily for 16 days, followed by 1.8 Gy twice daily for 9 days); cycle 2 chemotherapy [Cisplatin and Etoposide])

c) 5 weeks after the start of radiation therapy (completion of 61.2 Gy; mid-treatment for 70 Gy); mid-cycle 2 chemotherapy [Cisplatin and Etoposide])

d) 7 weeks after the start of radiation therapy (RT completed for all arms; cycle 3 chemotherapy [Cisplatin and Etoposide])

e) 12 weeks after the start of radiation therapy (5 weeks post-completion of 70 Gy arm; 6.5 weeks post completion of 61.2 Gy arm; 9 weeks post-completion of 45 Gy. Completion of chemotherapy (Cisplatin and Etoposide])

f) 26 weeks after the start of radiation therapy to assess longer-term consequences of patients' radiotherapy treatment on their quality of life

g) 52 weeks after the start of radiation therapy to assess long-term consequences of patients' radiotherapy treatment on patient's quality of life

h) Weekly during RT: ECOG Acute Esophagitis Scale and Difficulty Swallowing Item

The FACT-L, the FACT Eating and Swallowing Subscales, and the HADS are the only patient-reported quality of life measures that are translated into Spanish. Therefore, the quality of life component will be restricted to English-speaking patients only.

10.2.3 Measures

The Quality of Life battery will include measures of lung cancer-related physical symptoms, esophagitis, physical functioning, psychological state, quality-adjusted life years, treatment inconvenience, and sociodemographic characteristics, all of which (with the exception of sociodemographic characteristics) are hypothesized as being sensitive to differences in the treatment arms (see the table in Section 6.2). Acute esophagitis toxicity ratings will be obtained from the oncologist; all other quality of life data will be obtained from patient-completed questionnaires which will take the patient approximately 25 minutes to complete (see the table in Section 6.2 for the frequency of administering the different scales). Several measures that have been included in our study (FACT-Lung Cancer TOI, EQ-5D, difficulty swallowing item, all described below) are also being used in a radiation therapy trial in the RTOG (R0617) of 60 Gy versus 74 Gy in non-small cell lung cancer patients**.** This will create a large database by which to examine the effect of RT on the quality of life of both non-small cell and small cell lung cancer patients. The majority of patients in the study will have prophylactic cranial irradiation (PCI). The effect of PCI on quality of life measures is not clear. In the meta-analysis of PCI for limited stage small cell lung cancer [63] neuropsychological evaluation was performed in two trials [64, 65]. The initial neuropsychological assessment, performed in 350 patients, revealed that many patients had abnormalities prior to PCI. The results of repeated tests during the first years of follow-up revealed that the changes in neuropsychological function and the frequency of abnormalities

on CT scans of the brain did not differ between the treated and untreated patients. Consequently, an in-depth neuropsychological assessment of patients and the potential impact of cognitive deficits on physical functioning and psychological state were not deemed necessary. Moreover, the primary Quality of Life endpoints are determined prior to PCI administration. Whether patients received PCI will be noted in the analyses of secondary endpoints that occur in the follow-up period after completion of all therapy.

- ECOG Acute Esophagitis Toxicity Criteria (C-1733)

The ECOG Acute Esophagitis Criteria consist of 4 items involving patients' assessment of sore throat/dysphagia, analgesics required, nutrition/hydration, and weight loss [58]. The scoring system integrates multiple objective and subjective criteria in the determination of an overall esophagitis grade. Using this point system, an ECOG grade 0 equals 0 points, grade 1 equals 1-3 points, grade 2 equals 4-6 points, grade 3 equals 7-9 points and grade 4, 10-12 points. This will be completed by the oncologist on a weekly basis while the patient is receiving RT in each of the arms, and then at all subsequent assessments.

- Difficulty Swallowing (Form C-1736)

At the time of each assessment, patients will report swallowing difficulties based on their response to the following item: "Have you had any problem with swallowing today?" The responses are made on the following 5-point scale: 1=none; 2=mild soreness; 3=can swallow solids with some difficulty; 4=cannot swallow solids; 5=cannot swallow solids or liquids. Time of assessment is less than a minute.

- FACT-Esophagus Eating and Swallowing Subscales (Form C-1734)

The FACT-Esophagus Subscale consists of 17 items that are specific to patients with esophageal cancer, including eating, appetite, swallowing, pain, talking/communicating, mouth dryness, breathing difficulty coughing and weight loss [66]. All items are rated on a 5-point Likert scale ranging from 0, "not at all," to 4, "very much." For purposes of this study, in which esophagitis is one of the primary symptoms of radiotherapy, the Eating and Swallowing subscales will be used. The Swallowing Index consists of 5 items, and the Eating Subscale consists of 3 items. Internal consistency of both subscales is excellent, with the Eating Index having alpha coefficients of .84-.88, and the Swallowing Index having alpha coefficients of .82-.86. Evidence for validity was found in that for patients in the neoadjuvant chemoradiotherapy group, there was a significant improvement in the Eating and Swallowing Indices from time in treatment to post-treatment completion. It takes approximately 2-3 minutes to complete the scales.

- Functional Assessment of Cancer Therapy Scale: Lung Cancer (FACT-L) (Form C-1732)

The FACT-L [67] consists of the FACT-G [68] and the lung cancer subscale. The FACT-G (version 4.0), developed by Cella and colleagues [68] is an overall cancer-specific quality of life questionnaire. It is a 27-item core quality of life measure grouped into four subscales: physical well being, social/family well-being, emotional well-being, and functional well-being. The Lung Cancer subscale consists of 10 items, involving lung cancer-specific physical symptoms and regret about smoking. All items are rated on a 5-point Likert scale, ranging from 0, "not at all," to 4, "very much." The FACT-G has been tested on 545 patients with mixed cancer diagnoses. The internal consistency of the subscales ranges from moderate to excellent, from .65-.82, with excellent internal consistency of the total score, with an alpha coefficient of .89. Test-retest reliability is excellent within a 7-day period, with correlations ranging from .82-.92. Convergent validity has been demonstrated, with the FACT correlating significantly with other quality of life measures (FLIC, r=.79), and related constructs of psychological distress (e.g., Brief POMS, r=-.68), and the ECOG

performance rating (r=-.52) [68]. The FACT-G has been able to distinguish between patients with metastatic and non-metastatic disease. It has been determined that a 2 point difference on the FACT-G subscales and a 5 point difference on the FACT-G total score was associated with meaningful differences on clinical and subjective indicators [69]. In a review of the literature, Butt et al. [70] reported that the FACT-L scale has been used with more than 5,000 patients and been found to be sensitive to change in performance status over time, treatment response and correlate with dyspnea. The FACT-L is frequently used in clinical trials and studies of cancer patients' quality of life and is available in 20 foreign languages. The FACT-L will take approximately 10 minutes to complete.

The FACT-Lung Cancer (FACT-L) Trial Outcome Index (TOI) consists of the Physical Well-Being, Functional Well-Being and Additional Concerns (lung cancer-specific physical symptoms) subscales, for a total of 24 items [71]. It is a shortened version of the FACT-Lung Cancer Scale, which also includes Emotional and Social Well-Being. All items are rated on a 5-point Likert Scale, ranging from 0, "not at all," to 4, "very much." Evidence for validity of the FACT-L TOI was provided by finding that those with a significantly lower performance status had lower FACT-L TOI scores. Further, those patients with progressive disease had significantly lower TOI scores than those in the complete and partial responders groups. Last, those patients who progressed early had significantly larger negative declines in TOI scores than those who progressed later. Cella et al.'s [71] study has determined that a 5-7 point change in the FACT-Lung Cancer TOI is a clinically meaningful difference.

- Hospital Anxiety and Depression Scale (Form C-458)

The Hospital Anxiety and Depression Scale (HADS) [72] is a 14-item self-administered measure, which has been well tested in cancer populations. It has two 7-item subscales assessing depression and anxiety. The scale is considered particularly appropriate for use with medically ill patients because of the absence of somatic items, which often confound the determination of psychiatric problems in this population. Reported anxiety and depression cutoff scores on the HADS have varied from eight [73] to eleven [74]. The total cutoff score for psychological distress has ranged from 13, reflecting adjustment disorder, to 19, reflecting major depressive disorders [75]. Ibbotson and colleagues [76] found that for patients in active treatment, an overall cutoff score of 15 or greater resulted in 85% sensitivity, 77% specificity and a positive predictive value of 47%. For purposes of this study, 15 or greater on the HADS total score and 11 or greater on the HADS Anxiety and Depression subscales [74] will be used as the cutoff score indicative of a possible psychiatric disorder. The HADS will take approximately 8 minutes to complete.

- Subjective Significance Questionnaire (Form C-616)

In order to better interpret the clinical significance of changes in quality of life scores, Osoba et al. [77] developed the Subjective Significance Questionnaire (SSQ), consisting of 4 items in which patients rated the degree to which they saw an improvement in their physical condition, emotional state, ability to enjoy their social life and overall quality of life since their last quality of life assessment. All items are rated on a 7-point Likert scale, ranging from "very much worse" to "very much better," with each point on the scale anchored. The SSQ will be used to examine their scores in relation to corresponding changes in the FACT-L domains of quality of life. When used in relation to the EORTC QLQ C-30, increasing SSQ ratings were found to correspond with increasing EORTC scores in both breast and lung cancer patients, with small, moderate or large changes in quality of life defined in relation to changes in EORTC scores [78]. This will take 1 minute to complete.

- EQ-5D (Form C-903)

EQ-5D is a measure of health status for use in evaluating health and healthcare. It provides a simple descriptive profile of 5 functional dimensions and generates a single utility value for health status on which best imaginable health is assigned a value of 1 and worst imaginable health a value of 0. Thus, the index can be used to obtain a utility for these dimensions for use in economic analyses. The EQ-5D has been specially designed to complement other quality of life measures such as the SF-36, or cancer-specific measures. Descriptively, the EQ-5D consists of 5 dimensions, mobility, self-care, usual activity, pain/discomfort, and anxiety/depression. Each dimension has 3 levels designated simply as "no problem," "some problem," or "extreme problem," with patients checking the level most descriptive of their current level of function on each dimension. Five dimensions, each with three levels, yield 243 possible distinct health states comprising the classification system. The classification system has been assigned different standardized scores derived through population-based samples of respondents who assign values to subsets of the 243 states using the anchoring labels noted above [79]. A set of valuation weights has thus been derived from a U.S. sample more recently [79, 80]. The Agency for Healthcare Research and Quality has funded a study to develop definitive weights. EQ-5D is designed for self-completion by patients and has been used extensively in mailed surveys. It is cognitively simple, taking no more than a few minutes to complete. Quality-adjusted life years can be calculated from using the EQ-5D in conjunction with a utility scale of health states.

- Treatment Inconvenience (Form C-1735)

The different treatment arms have different dose schedules for different lengths of times, which may impact the satisfaction with treatment due to the inconvenience posed. Therefore, an item will be added as to the inconvenience of going for therapy at the different dose schedules.

- Sociodemographic Characteristics (Form C-187)

Demographic data, including education, marital status, employment and household composition, will be obtained at baseline only using the CALGB Background Information Form [81] (CALGB Form C-187). Age, gender, and ethnicity will be obtained upon patients' registration to the study.

- QOL Assessment Form (Form C-419, revised)

In order to track data collection, and reasons for missing data, a QOL Assessment Summary Form will be completed for each patient by the CRA, for each assessment, coding whether the assessment was conducted and data collected, and if not, the reason why.

11.0 Drug Formulation, Availability, and Preparation

- Qualified personnel who are familiar with procedures that minimize undue exposure to themselves and to the environment should undertake the preparation, handling, and safe disposal of chemotherapeutic agents in a self-contained, protective environment.
- Discard unused portions of injectable chemotherapeutic agents that do not contain a bacteriostatic agent or are prepared with unpreserved diluents (i.e., Sterile Water for Injection USP or 0.9% Sodium Chloride for Injection USP) within eight hours of vial entry to minimize the risk of bacterial contamination.
- The total administered dose of chemotherapy may be rounded up or down within a range of 5% of the actual calculated dose.

11.1 Cisplatin

Please refer to the FDA approved package insert for additional information.

Cisplatin (DDP): Cisplatin (Platinol; cDDP; Platinum, Platinol AQ; cis-DDP; cis-Diamminedichloroplatinum; cis-Platinum II.

Availability

Platinol (Bristol-Myers Oncology Division) is commercially available as an aqueous solution (platinol AQ 1 mg/ml injection in 50 ml and 100 ml vials).

Compatibility

Incompatible with dextrose solutions (or any solution) containing less than 0.2% sodium chloride. Y-site incompatibility: Chlorpromazine, Piperacillin/Tazobactam.

Storage and Stability

Reconstituted vials are stable for 20 hours at room temperature. Vials reconstituted with bacteriostatic solutions are stable for 72 hours. Intact vials of cisplatin for injection and powder for injection should be stored at room temperature and protected from light.

Do not refrigerate.

Aluminum reacts with cisplatin to form black precipitates and loss of potency; do not prepare or administer with aluminum needles or IV sets; stainless steel or plated brass hubs may be used.

Toxicities

Nephrotoxicity (dose related and severe); electrolyte abnormalities (increased excretion of Mg, K, Ca, PO4, Na); hyperuricemia; ototoxicity (30%, particularly high frequency hearing); nausea/vomiting; anaphylaxis/hypersensitivity; cardiotoxicity (rare: bradycardia, CHF); neurotoxicity (peripheral neuropathies, myasthenic-like syndrome); myelosuppression (moderate and reversible; infrequent at low dose); elevations in liver enzymes; optic neuritis; SIADH; seizures; cortical blindness (rare); loss of taste.

11.2 Carboplatin

Availability

Carboplatin is commercially available and supplied as a sterile lyophilized powder available in single-dose vials containing 50 mg, 150 mg, 450 mg, or 600 mg of carboplatin. Each vial contains equal parts by weight of carboplatin and mannitol.

Refer to the package insert for further information.

Preparation

Prepare according to institutional standards.

Storage and Stability

Unopened vials of carboplatin are stable for the life indicated on the package when stored at controlled room temperature and protected from light.

Administration

AUC dosing will be used to determine carboplatin dosing. The protocol permits use of the Cockcroft-Gault formula to estimate creatinine clearance. However, in markedly obese patients, the Cockcroft-Gault formula will tend to overestimate the creatinine clearance. The actual body weight (in kilograms) will be utilized in the Cockcroft-Gault formula. However, if the calculated creatinine clearance exceeds an upper limit for creatinine clearance, as specified below, then this ceiling value for creatinine clearance, rather than the calculated creatinine clearance, will be used in the Calvert formula to calculate the dose of carboplatin.

The maximum allowable creatinine clearance for males and females is 125 ml/min.

Alternatively, at the treating physician's discretion, a measured 24 hour creatinine clearance can be obtained. In this case, the measured creatinine clearance can be used to calculate the carboplatin dose in the Calvert formula.

Toxicities

Myelosuppression, nausea and vomiting, peripheral neuropathy, nephrotoxicity, hepatotoxicity, electrolyte imbalance, hypomagnesemia, hypocalcemia, and allergic reactions.

11.3 Etoposide

Please refer to the FDA approved package insert for additional information.

Availability

Intravenous etoposide is commercially available (VePesid Injection from Bristol-Myers Oncology) in ampules containing 1000 mg/50 ml, 500 mg/25 ml, 150 mg/7.5 ml, and 100 mg/5 ml. It is now available from generic sources and is available in a 20 mg/ml, 1 gm vial.

Preparation

The dose of etoposide should be further diluted with D5W or Normal Saline for Injection to a final concentration of less than 0.4 mg/ml.

Storage and Stability

Unopened vials are stable at room temperature for 24 months. Vials diluted up to a concentration of 0.2 or 0.4 mg/ml are stable for 96 and 24 hours, respectively, at room temperature under normal light.

Administration

Administer the diluted infusion solution at a maximum rate of 500 mg/hr; an administration that is too rapid may be associated with hypotension.

Toxicities

Myelosuppression, anorexia, nausea and vomiting, headaches, alopecia, phlebitis, fever, and peripheral neuropathy may occur. Acute arterial hypotension may result from rapid intravenous infusion. Anaphylaxis, somnolence and fatigue, rash, pigmentation, urticaria and pruritis may also occur.

12.0 ANCILLARY THERAPY

- Patients should receive full supportive care, including transfusions of blood and blood products, erythropoetin, antibiotics, antiemetics, etc., when appropriate. The reason(s) for treatment, dosage, and the dates of treatment should be recorded on CALGB Form C-260 (Remarks Addenda).
- Treatment with hormones or other chemotherapeutic agents may not be administered except for steroids given for adrenal failure; hormones administered for non-disease-related conditions (e.g., insulin for diabetes); and intermittent use of dexamethasone as an antiemetic.

12.1 Alliance Policy Concerning the Use of Growth Factors

The following guidelines are applicable unless otherwise specified in the protocol:

12.1.1 Epoetin (EPO)

The use of EPO is **permitted** at the discretion of the treating physician.

12.1.2 Filgrastim (G-CSF) and sargramostim (GM-CSF)

1. Filgrastim (G-CSF), pegfilgrastim and sargramostim (GM-CSF) treatment is discouraged.
2. Filgrastim/pegfilgrastim and sargramostim may not be used:

   a. to avoid dose reductions, delays or to allow for dose escalations specified in the protocol,

   b. prophylactically because of concern about myelosuppression from prior chemotherapies
3. For the treatment of febrile neutropenia the use of CSFs should not be routinely instituted as an adjunct to appropriate antibiotic therapy. However, the use of CSFs may be indicated in patients who have prognostic factors that are predictive of clinical deterioration such as pneumonia, hypotension, multi-organ dysfunction (sepsis syndrome) or fungal infection, as per the ASCO guidelines. Investigators should therefore use their own discretion in using the CSFs in this setting. The use of CSF (filgrastim/pegfilgrastim or sargramostim) must be documented and reported on CALGB Form C-260 (Remarks Addenda).
4. If filgrastim/pegfilgrastim or sargramostim are used, they must be obtained from commercial sources.

13.0 CRITERIA FOR RESPONSE, PROGRESSION, AND RELAPSE

For the purposes of this study, patients should be reevaluated every 6 weeks. In addition to a baseline scan, confirmatory scans should also be obtained ≥ 4 weeks following initial documentation of objective response.

13.1 Target Lesions

All measurable lesions up to a maximum of 10 lesions representative of all involved organs should be identified as target lesions and will be recorded and measured at baseline. Target lesions should be selected on the basis of their size (lesions with the longest diameter) and their suitability for accurate repetitive measurements (either by imaging techniques or clinically). A sum of the longest diameter (LD) for all target lesions will be calculated and reported as the baseline sum LD. The baseline sum LD will be used as reference to further characterize the objective tumor response of the measurable dimension of the disease.

13.1.1 Complete Response: Disappearance of all target lesions. Where response is the primary endpoint, changes in tumor measurements must be confirmed by repeat studies performed no less than 4 weeks after the criteria for response are first met.

13.1.2 Partial Response (PR): At least a 30% decrease in the sum of the longest diameter (LD) of target lesions taking as reference the baseline sum LD. Where response is the primary endpoint, changes in tumor measurements must be confirmed by repeat studies performed no less than 4 weeks after the criteria for response are first met.

13.1.3 Progression (PD): At least a 20% increase in the sum of the LD of target lesions taking as references the smallest sum LD recorded since the treatment started or the appearance of one or more new lesions.

13.1.4 Stable Disease (SD): Neither sufficient shrinkage to qualify for PR nor sufficient increase to qualify for PD taking as references the smallest sum LD since the treatment started. Patients having a documented response with no reconfirmation of the response will be listed with stable disease.

13.2 Non-target Lesions

All other lesions (or sites of disease) not included in the "target disease" definition should be identified as non-target lesions and should also be recorded at baseline. Measurements are not required and these lesions should be followed as "present" or "absent."

13.2.1 **Complete Response (CR):** Disappearance of all non-target lesions and normalization of tumor marker level. If tumor markers are initially above the upper normal limit, they must normalize for a patient to be considered in complete clinical response.

13.2.2 **Non-complete response (non-CR)/Non-progression (non-PD):** Persistence of one or more non-target lesion and/or maintenance of tumor marker level above the upper limits of normal.

13.2.3 **Progression (PD):** Appearance of one or more new lesions. Unequivocal progression of existing non-target lesions. Although a clear progression of non-target lesions is exceptional, in such circumstances, the opinion of the treating physician should prevail and the progression status should be confirmed later on by the study chair.

13.3 Cytology and Histology

If the measurable disease is restricted to a solitary lesion, its neoplastic nature should be confirmed by cytology/histology.

These techniques can be used to differentiate between PR and CR in rare cases (for example, residual lesions in tumor types such as germ cell tumors, where known residual benign tumors can remain).

The cytological confirmation of the neoplastic origin of any effusion that appears or worsens during treatment when the measurable tumor has met criteria for response or stable disease is mandatory to differentiate between response or stable disease (an effusion may be a side effect of the treatment) and progressive disease.

13.4 Evaluation of Best Overall Response

The best overall response recorded from the start of the treatment until disease progression/recurrence (taking as reference for progressive disease the smallest measurements recorded since the treatment started). In general, the patient's best response assignment will depend on the achievement of both measurement and confirmation criteria (see Section 13.6.1).

Target Lesions	Non-target Lesions	New Lesions	Overall Response
CR	CR	No	CR
CR	Non-CR/Non-PD	No	PR
PR	Non-PD	No	PR
SD	Non-PD	No	SD
PD	Any	Yes or No	PD
Any	PD	Yes or No	PD
Any	Any	Yes	PD

Note:

- Patients with a global deterioration of health status requiring discontinuation of treatment without objective evidence of disease progression at that time should be reported as "symptomatic deterioration" on the Off-treatment Form (C-300) under "other." Every effort should be made to document the objective progression even after discontinuation of treatment.
- Conditions that may define "early progression, early death and inevaluability" are study specific and should be clearly defined in each protocol (depending on treatment duration, treatment periodicity).

For example: Conditions that may define early death include patients that have died without documentation of disease progression and before it was time to conduct the first tumor reassessment. Inevaluable patients have received protocol treatment (regardless of how much was received) and did not have any follow-up assessment completed before initiation of alternative treatment.

- In some circumstances it may be difficult to distinguish residual disease from normal tissue. When the evaluation of complete response depends upon this determination, it is recommended that the residual lesion be investigated (fine needle aspirate/biopsy) before confirming the complete response status.

13.5 Guidelines for Evaluation of Measurable Disease

13.5.1 Clinical Lesions will only be considered measurable when they are superficial (e.g., skin nodules, palpable lymph nodes). For the case of skin lesions, documentation by color photography including a ruler to estimate the size of the lesion is recommended.

13.5.2 Chest X-ray: Lesions on chest X-ray are acceptable as measurable lesions when they are clearly defined and surrounded by aerated lung. However, CT is preferable.

13.5.3 Conventional CT and MRI: Sections of 5 mm thickness (or less) will be obtained using spiral/helical technique. Reconstruction intervals must be equal of less than the section thickness. Anatomic coverage will extend from lung apices through the adrenal glands. IV contrast enhancement is required, unless the patient has significant history of contrast allergy. A CT scan performed at a site other than a study site may be acceptable for study purposes; otherwise, it should be repeated at the study site. In particular, a repeat CT scan should be performed if the investigator at the study site considers the outside CR scan to be of poor quality.

13.5.4 Ultrasound (US) should not be used to measure tumor lesions that are clinically not easily accessible when the primary endpoint of the study is objective response evaluation. It is a possible alternative to clinical measurements of superficial palpable nodes, subcutaneous lesions, and thyroid nodules. US might also be useful to confirm the complete disappearance of superficial lesions usually assessed by clinical examination.

13.5.5 Endoscopy and Laporascopy for objective tumor evaluation has not yet been fully and widely validated. Their uses in this specific context require sophisticated equipment and a high level of expertise that may only be available in some centers. Therefore, the utilization of such techniques for objective tumor response should be restricted to validation purposes in reference centers. However, such techniques can be useful to confirm complete pathological response when biopsies are obtained.

13.5.6 Tumor Markers alone cannot be used to assess response. If markers are initially above the upper normal limit, they must normalize for a patient to be considered in complete clinical response.

13.6 Confirmation Measurement/Duration of Response

13.6.1 Confirmation

To be assigned a status of PR or CR, changes in tumor measurements must be confirmed by repeat studies that should be performed 4 weeks after the criteria for response are first met. In the case of SD, follow-up measurements must have met the SD criteria at least once after study entry at a minimum interval of 6 weeks.

13.6.2 Duration of Overall Response

The duration of overall response is measured from the time measurement criteria are met for CR/PR (whichever is first recorded) until the first date that recurrent or progressive disease is objectively documented (taking as reference for progressive disease the smallest measurements recorded since the treatment started).

The duration of overall complete response is measured from the time measurement criteria are first met for CR until the first date that recurrent disease is objectively documented.

13.6.3 Duration of Stable Disease

Stable disease is measured from the start of the treatment until the criteria for progression are met, taking as reference the smallest measurements recorded since the treatment started.

14.0 REMOVAL OF PATIENTS FROM PROTOCOL THERAPY

14.1 Duration of Treatment

14.1.1 CR, PR, or SD: Continue treatment at the highest tolerable dose for up to 4 cycles until the appearance of disease progression.

14.1.2 Disease Progression: Remove from protocol therapy any patient with rapid disease progression. Document details, including tumor measurements, on data forms.

14.2 Extraordinary Medical Circumstances:

If, at any time the constraints of this protocol are detrimental to the patient's health and/or the patient no longer wishes to continue protocol therapy, protocol therapy shall be discontinued. In this event:

- Notify the Study Chair.

- Document the reason(s) for discontinuation of therapy on CALGB Form C-260.
- Follow the patient for progression, survival, new primaries or secondary malignancy for a total of 10 years following registration.

15.0 STATISTICAL CONSIDERATIONS

15.1 Endpoints

15.1.1 Primary Endpoint

The primary objective of this study is to determine whether administering high dose thoracic radiotherapy, 70 Gy (2 Gy once-daily over 7 weeks) or 61.2 Gy (1.8 Gy once-daily for 16 days followed by 1.8 Gy twice-daily for 9 days), will improve overall survival compared with 45 Gy (1.5 Gy twice-daily over 3 weeks) in patients with limited stage small cell lung cancer. Overall survival time is measured from randomization until death from any cause.

15.1.2 Interim Endpoint

An interim endpoint will be utilized to select between the experimental treatment arms. Treatment related grade 3+ non-hematologic toxicity, grade 4 hematologic toxicity and any grade 5 toxicity will be used in this determination. Discontinuing the experimental arm with higher toxicity will not only protect patients but also substantially reduce the study size. The rationale of eliminating an experimental arm based on acute toxic effect of therapy is well founded given that the acute toxicity of the standard regimen (e.g., 45 Gy BID TRT) appears to have substantially limited its acceptance into clinical practice. Moreover, both experimental arms have greater predicted biologic efficacy compared with standard treatment. The focus will be on grade 3+ treatment related non-hematologic toxicity, grade 4 hematologic toxicity, failure to complete 4 cycles of chemotherapy and any grade 5 toxicity. The following scoring system will be used to define the severity of adverse events. For each patient, only the highest grade of relevant toxicities will be counted and multiple relevant toxicities of the same grade will be counted once.

Any grade 2 or less toxicity or grade 3 hematologic toxicity	0 point
Grade 3 non-hematologic toxicity or grade 4 hematologic toxicity	1 point
Grade 4 non-hematologic toxicity or failure to complete 4 cycles of chemotherapy	2 points
Any grade 5 toxicity	3 points

In the initial stage of the study, eligible patients will be randomized to arms 1, 2 and 3 with probability ratio of 1:1:1. A blocking design with 30/30/30 patients will be used to compare toxicity between the experimental arms. Specifically, after 30 patients have been accrued to each experimental arm, the Alliance Statistics and Data Center will computerize and analyze all treatment related toxicities and report findings to the study team and the DSMB. For the 60 patients treated by experimental regimens, permutation t test will be used to compare toxicity severity scores between experimental arms. If the 2-sided p value of the permutation test is less than 0.05, a decision will be made to drop the experimental arm with higher toxicity; otherwise, the study will continue and adverse events will be analyzed again after 20 additional patients have been treated in each experimental arm. For the 100 patients treated by experimental regimens so far, if statistically significant difference of adverse events is found between the experimental arms, a decision will be made to drop the

experimental arm with higher toxicity; otherwise, the study will continue after 20 additional patients have been treated in each experimental arm. For the first 140 patients treated by experimental regimens, the experimental arm with the highest cumulative numerical toxicity score will be discontinued following review by the study team and the DSMB.

A notice will be broadcast to all Alliance and CTSU institutions for the discontinuation of an experimental arm. In the initial stage of the trial, while the toxicity profiles of treated patients are being evaluated, the study accrual will not be suspended. In the second stage, the study will proceed with the remaining experimental arm and the standard treatment arm with 1:1 allocation.

15.1.3 Secondary Endpoint

Secondary endpoints will include complete and partial response rates, failure-free survival, local tumor progression according to RECIST criteria, and rates of distant metastases and intracranial metastases. Failure free survival is the time from randomization to death of all causes or disease progression, whichever comes first. Second primary tumors will not be viewed as disease progression.

15.2 Stratification Factors

- Gender: male vs. female
- Weight loss (6 months prior to study entry): <= 5 % vs > 5%
- ECOG Performance Status: 0 vs 1 vs 2
- Radiotherapy Technique: IMRT vs. 3D
- Radiotherapy Start Time (choose one):

  -At first cycle of protocol chemotherapy, after one cycle of prior non-protocol chemotherapy
  -At first cycle of protocol chemotherapy, without prior non-protocol chemotherapy
  -At second cycle of protocol chemotherapy, without prior non-protocol chemotherapy
- Chemotherapy Backbone: carboplatin vs. cisplatin

15.3 Sample Size with Power Justification

This randomized phase III study is to determine the efficacy of 70 Day QD RT (arm 2) or 61.2 Gy concurrent boost RT (arm 3) as compared to the standard therapy 45 Gy BID RT (arm 1) in patients with limited small cell lung cancer. The primary endpoint is overall survival, which is measured from the date of randomization to the date of death from any cause.

The implementation of the study will be divided into two stages. In the first stage, eligible patients will be randomized with a 1:1:1 allocation to arm 1, 2 and 3. The toxicity profiles of two experimental arms (arm 2 and arm 3) will be analyzed after 30 patients have been treated to each of the experimental arms. If no statistically significant difference of adverse events is found between experimental arms, additional 20 patients will be treated by each experimental arm. The pattern will be repeated until a significant difference is found or 70 patients have been treated by each experimental arm, whichever comes first. In the second stage, after the decision of dropping the experimental arm with higher toxicity is made, the study will continue with two remaining arms with 1:1 allocation. All randomization will be made using a permuted-block scheme, stratified by gender (female vs. male), ECOG PS (0 vs. 1 vs. 2), weight loss within 6 months before entry (weight loss ≤5% vs. >5%), radiotherapy technique: IMRT vs. 3D, timing of first chemotherapy treatment (prior to randomization vs. post-randomization with the start of radiotherapy on the first cycle vs. post randomization with the start of radiotherapy on the second cycle), and chemotherapy backbone (carboplatin vs. cisplatin) [82]. In the end, the study will

randomize 303 patients to arm 1, 303 to Arm 2 and 30-70 patients to the discontinued experimental arm. As of November 20, 2012, the discontinued experimental arm (Arm 3) randomized 88 patients due to a delayed final toxicity interim decision. With an allowance of 5% of patients canceling or ineligible for randomization, the study will accrue a total of 729 patients over a period of 6 years at about 10 patients per month. All randomized patients will be followed for at least 2.5 years after enrollment

The trial is designed to have adequate power to test the null hypothesis H_0: $\log(\lambda_1 / \lambda_{2/3})=0$ versus the alternative hypothesis Ha: $\log(\lambda_1 / \lambda_{2/3}) \geq 1.3$ for the comparison of overall survival between the remaining experimental arm (arm 2) and the standard arm (arm 1). The power analysis is based on the following assumptions: (1) the 2-year overall survival rate of the remaining experimental arm (arm 2) is 57.3% and that of arm A is 48.5%. Under exponential survival distribution, the median survival for the remaining experimental arm is 29.9 months and for the standard arm is 23 months, or a hazard ratio of 1.3 (λ_1 / λ_2) for the standard arm to the remaining experimental arm; (2) an accrual length of 6 years; (3) 303 eligible patients in the standard arm and 303 eligible patients in the remaining experimental arm; (4) additional follow-up of 2.5 years after the last enrollment; (5) a two-sided significance level of 0.05. Under these assumptions, at the time of final analysis, at least 483 deaths are anticipated under alternative hypotheses for the two remaining comparative arms. Without taking into account the expected minimal loss of power due to interim analyses, the power in detecting the survival improvement for the remaining experimental arm (arm 2) as compared to the standard arm is at least 81.9% using a stratified log-rank test.

15.4 Analysis Plan Including Plans for Formal Interim Analysis

Both short term and long term toxic effects of therapy will be captured and monitored closely. Severe toxic events will be reported and monitored as per NCI guidelines for clinical studies (e.g., CTEP-AERS). These data will be critical in the assessment of each of the treatment regimens. In addition, the Alliance DSMB will perform a formal review of the available data at each of its semiannual meetings. This review will include both acute and late toxicity.

After discontinuing one of the experimental arms with worse toxicity profile, in the second stage of the study, the first formal interim analysis for efficacy will occur once 78 deaths are observed in the remaining study arms. After that, formal interim analyses will be conducted annually, with the final analysis at year 8.5, corresponding to approximately 140, 213, 292, 374, 437 and 483 deaths. The trial is subject to stopping early for either superiority or inferiority of the experimental relative to the standard arm if either arm was markedly superior in terms of overall survival. The main reason is that if both arms were roughly equal in terms of overall survival, the final conclusion might be to recommend the experimental arm for future use because of less toxicity or more convenience. Using S+SeqTrial [83], we will construct two-sided symmetric monitoring boundaries in the spirit of O'Brien and Fleming [84]. To maintain overall 1-sided alpha level of 0.025 for each boundary and be conservative in the initial looks, we will truncate alpha level at 0.0005 for the first look and at 0.0025 for other interim looks [85]. The final analysis at 8.5 years after study activation will conclude superiority (inferiority) of the remaining experimental arm to the standard arm if the p-value of a one-sided log rank test for superiority (inferiority) is less than 0.0220. The following table displays the operating characteristics, including power, average study size, stopping probabilities under true hazard ratios of 0.75, 1.0, 1.3, and 1.5. The exact performance of the study design, especially under different allocation ratios in the first and the second stage of the study, will be investigated using simulation.

Hazard Ratio (λ_1/ $\lambda_{2/3}$)	Expected Number of Deaths	Prob. to Reject H_0: λ_1 / $\lambda_{2/3}$ =1 (Power)	Early Stop Prob. for Superiority	Early Stop Prob. for Inferiority
0.75	354	0.8763	0.0000	0.6348
1.0	479	0.0250	0.0081	0.0081
1.3	377	0.8106	0.5357	0.0000
1.5	245	0.9928	0.9385	0.0000

The Alliance DSMB will review the data available from the trial at each of its semiannual meetings. This will include toxicity, disease recurrence and survival information. The DSMB will determine which of its biannual meetings most appropriately fits the protocol-dictated one-year formal interim analysis. This determination will be based on a recommendation of the study statistician. In determining whether the trial should be continued, the DSMB will consider the results at each interim analysis, as described above. The DSMB will use its discretion in weighing the combined impact of treatment-related morbidity, disease recurrence and overall survival.

Overall survival (OS) is defined as the time between randomization and death from all causes. Overall survival curve will be estimated by the Kaplan-Meier method. The primary analysis is to test the survival benefit of the experimental therapy over the standard therapy using stratified log rank test in the intent-to-treat (ITT) population. Six stratification factors, including Gender, Weight loss, ECOG Performance Status, Radiotherapy Technique, Radiotherapy Start Time, and Chemotherapy Backbone, have been used in randomization. As a total of 144 stratums have been defined by these factors and many of the stratum may end up of small number of patients. For the primary analysis, the stratified log rank test will only stratify on patient subgroups defined by Performance Status (0 vs 1/2) and Chemotherapy Choice (entry prior to the option to use carboplatin, choice of carboplatin among those patients for which this is an option, and choice of cisplatin among those patients for which this is an option). Cox's proportional hazards model will be used to estimate hazard ratio between treatments with and without adjusting for other prognostic factors.

Failure free survival (FFS) is the time from randomization to death of all causes or disease progression, whichever comes first. In this study, second primary tumors will not be viewed as disease progression. FFS will be analyzed in a similar manner to overall survival (OS).

The rates of local relapse, distant metastases and brain metastases with the regimens will be evaluated using logistic regression with treatment and other prognostic factors in the model. .

Contingency tables will be used to summarize the frequency of toxicity by severity, type and treatment. For comparison of the frequency of toxic events, the Cochran-Mantel-Haenszel test will be used to take advantage of the trend effect among toxicity grades.

15.5 Logistics and Accrual

An estimated 174,000 patients will be diagnosed with lung cancer in 2006. Small cell lung cancer comprises approximately 16-20% of all lung cancer, and accounts for 22% of all lung cancer cases accrued to cooperative group clinical trials. According to the National Cancer Data Base, 42% of staged patients had limited stage disease in 2000, and 11,000 to 14,000 new cases of limited stage small cell lung cancer are expected annually. The total accrual goal is 729 patients. To complete accrual in 6 years, the accrual rate will be approximately 10 patients per month (120 patients per year). We anticipate this trial to be opened by all major Alliance and RTOG sites, and several additional adult cooperative groups including SWOG and ECOG have

agreed to participate in this trial. The accrual goal should be achievable given the track record of accrual to limited stage small cell lung cancer trials in these Groups. Given the uncertainty in clinical practice regarding the optimal thoracic radiotherapy regimen in limited stage small cell lung cancer, we expect that the question addressed by this study will lead to a high level of interest and participation. In addition, there are few competing cooperative group, industry, or institutional studies in this patient population.

15.6 Correlative Studies (CALGB 150712)

In the main treatment study, a total of 303 patients will be randomized to the standard control arm (arm A) and the remaining experimental arm (arm B or arm C, depending on the toxicity data from an initial cohort, the more toxic experimental arm will be dropped). These patients will be followed for disease progression and survival for at least 3 years after enrollment. All patients will be invited to join the correlative science substudies. Samples from consenting patients will be collected at baseline (before treatment initiation), day 21 (pre-chemotherapy), at first follow-up after completion of all protocol treatment, and at the time of disease recurrence. The laboratory data, including measures of all biomarkers described in the protocol, will be sent to the Alliance Statistics and Data Center, where the laboratory data will be merged with the clinical data and analyzed by Alliance statisticians.

Power Analysis for the First Correlative Science Substudy (Section 10.1.1):

The primary objectives of this first correlative science substudy are (i) to test whether higher-dose, accelerated radiation therapy (arm B or C) will cause a larger rise in circulating markers of endothelial damage than hyperfractionated and accelerated radiotherapy (arm A), and (ii) to determine whether the baseline biomarker profile, or changes in the profile during treatment, correlate with clinical outcome as judged by objective response rate, progression free survival, and overall survival.

With allowance of 20% patient loss due to consent, ineligibility, and specimens of poor quality, we assume that 264 patients on arm A and 264 patients on arm B/C will provide evaluable circulating biomarkers for final analysis. With regards to the first primary objective, we assume the transform of biomarker count difference between post-treatment initiation and baseline follows approximately a normal distribution so that a 2-sample t test would be appropriate. At a 2-sided significance level of 0.05, the study has 90% power to detect a 0.274 standard deviation of cell count difference between the two treatment arms on log scale. With regards to the second primary objective, for the purpose of power analysis, the correlation of the biomarker (baseline or change scores of post-initiation of treatment against baseline) with response rate, 2-year FFS and 2-year OS will be tested for each arm using logistic regression with a single continuous predictor. At a 2-sided significance level of 0.05, the study has at least 90% power to detect a 6% increase of response rate from 80% at the mean of the biomarker to 86% at one standard deviation above of the mean. Similarly, the study has at least 90% power to detect an 11% increase of 2-year FFS from 40% at the mean to 51% at one standard deviation above of the mean, to detect an 11% increase of 2-year OS from 50% at the mean of a biomarker to 61% at one standard deviation above of the mean.

Power Analysis for the Second Correlative Science Substudy (Section 10.1.2):

The primary objective of the second correlative substudy is to evaluate the correlation of several serum markers at baseline or their changes against baseline with response rate (RR), failure free survival (FFS), and overall survival (OS) in small cell lung cancer. They include neuron-specific enolase (NSE), carcinoembryonic antigen (CEA), BB isoenzyme of creatine kinase (CK-BB), angiogenic factors Interleukin 8 (IL-8) and Vascular Endothelial Growth Factor (VEGF), stem cell factor (SCF), granulocyte-macrophage-colony stimulating factor (GM-CSF), and tumor

hypoxia marker osteopontin. The correlation tests are considered exploratory and the Type I errors will not be adjusted for multiple comparisons.

It is assumed that 230 randomized patients (70%) on each arm will consent and provide valid measures for these biomarkers. These biomarkers are concentration in the blood. The baseline measures and the change at different time points – day 21; at first follow-up after completion of all protocol treatment; and at the time of disease recurrence – against baseline are continuous variables. For the purpose of power analysis, logistic regression with a single continuous predictor will be used to test a measure's correlation with RR, 2-year FFS, and 2-year OS, separated by study arm. At a 2-sided significance level of 0.05, the study has at least 90% power to detect a 6% increase of response rate from 80% at the mean of the biomarker to 86% at one standard deviation above of the mean. Similarly, the study has at least 90% power to detect a 12% increase of 2-year FFS from 40% at the mean to 52% at one standard deviation above of the mean, to detect a 12% increase of 2-year OS from 50% at the mean of a biomarker to 62% at one standard deviation above of the mean.

Power Analysis for the Third Correlative Science Substudy (Section 10.1.3):

Most studies of proGRP have reported that approximately 70-80% of patients have increased levels of proGRP in their serum or plasma. The primary endpoint examines the correlation of increases in the plasma proGRP levels with changes in disease status. This strategy is based on the analyses performed for tumor markers used for monitoring and approved by the FDA, such as CA19-9, CA15-3, CA125 and CEA. Changes above a cutoff are compared for to clinical evaluation of stable disease vs. progression. In the secondary studies, the correlation of decreases in proGRP level will be compared to stable disease vs. regression and/or remission designations. The data is analyzed as concordance of 2 X 2 table of clinical outcome vs. a defined change in cutoff levels. For proGRP, 3 levels will be evaluated initially a) the percent increase that is 2.5 times the assay's observed total percent coefficient of variation (CV), b) a percent change that is 2.5 times the assay and biological variation, and c) a change of >25% based on changes used with other cancer biomarkers. The change in disease state is evaluated per sequential pair and per patient. Secondary analyses can determine whether a different cutoff functions better than these 3 cutoffs. Individual patients will be evaluated by following their sequential proGRP values and their clinical status via tables and graphs.

Based on the numbers of patients and samples sufficient for these evaluations for other monitoring biomarkers as submitted to and approved by the FDA, it is recommended that between 150 and 200 patients be enrolled in this study. A sample size of approximately 150 and 200 patients will provide a sufficient number of evaluable patients with 95% confidence and 80% to 90% statistical power for evaluating the association of the change in ProGRP assay values and change in disease status.

For the primary study and the secondary studies of the relationship of proGRP levels at baseline and at each round of chemotherapy and at the end of therapy will be analyzed as defined above.

Analytic Methods

All data from eligible patients with evaluable measures of the biomarker of interest will be included in final analysis. A 2-sided significance level of 0.05 will be used for all tests.

Descriptive statistics will used to characterize biomarker count or distribution over time and changes against baseline. The change of biomarker count or mean between treatment arms will be tested using a 2-sample t test on a transformed scale such as the use of t test is appropriate. If the normal assumption is violated, Wilcoxon rank sum test, a nonparametric test, will be used. As supplementary analysis, the difference of biomarker count and mean change will be modeled using generalized linear model using appropriate link function.

The correlation of the baseline measure or the change of the biomarker measure relative to baseline with response rate (RR) will be evaluated using logistic regression [86] with and without adjusting for significant confounding variables at baseline. The correlation of the biomarker with failure free survival (FFS) and overall survival (OS) will be evaluated using Cox's proportional hazard model [87] for time to event data without and with adjusting for confounding variables. These analyses will reveal whether the baseline biomarker or its change against baseline during treatment is predictive to RR, FFS, or OS, or whether the correlation is dependent on which treatment is given. In the pooled analysis of all patients on the treatment arms, the interaction between treatment and baseline or change score of the biomarker will be tested to evaluate the predictive value of the biomarker to different treatments.

If a continuous biomarker is significantly correlated with FFS or OS, an optimal threshold for classifying patients into subgroups will be identified by searching the dichotomized level of the continuous predictor at which yields the maximal log rank statistic. Both the raw p-value from the log rank test as well as the adjusted p-value from the maximal log rank statistic [88], which takes the randomness of the maximally selected cutoff point into account, will be provided. Kaplan-Meier curves [89] will be used to describe survival difference for different subgroups of patients classified by predictive biomarker at its optimal cutoff point.

To assess the correspondence between increases in plasma ProGRP and disease progression or recurrence, patients will be considered evaluable if they have at least five blood draws, one at baseline and the remaining draws at 3, 6, 9 and 12 months, respectively with the corresponding scans at those time points. Patients with CR, PR, and SD will be grouped together as non-progressive disease for the statistical analysis. Since each patient contributes a unique set of visit pairs to the data, this must be controlled by using techniques developed for generalized linear models. GENMOD procedure in SAS will be used to obtain the estimates of the concordances. The relationship between the concordance estimate C and the parameter estimate b is given by $C = (1 + e^{-b})^{-1}$. 95% confidence limits will be determined for all concordance proportion.

To evaluate the ability of ProGRP at baseline, after each cycle of chemotherapy and at first evaluation post-treatment, patients will be grouped into two different groups based on their ProGRP concentrations. At each of those intervals, they will be grouped as negative if (ProGRP $\leq$ 50 pg/mL) or as positive if (ProGRP > 50 pg/mL). In addition, changes in ProGRP concentrations starting at baseline and after the first or second dose of chemotherapy will also be explored. The log-rank test will be used to determine if the PFS for patients with positive values (or changes) are different from the negatives.

15.7 Health Related Quality of Life (CALGB 70702)

Hypothesis A: Patients in the hyperfractionated accelerated dose (45 Gy) group will experience worse lung cancer specific physical symptoms and physical functioning, as measured by the FACT TOI (Trial Outcome Index) -Lung Cancer and FACT-Esophageal Cancer Eating and Swallowing Indices, than those in the high dose daily (70 Gy) and concomitant boost radiotherapy (61.2 Gy) groups when all RT has been completed in each arm of the study at 12 weeks after the start of radiation therapy. This hypothesis will be revised to reflect the change in the research design due to the elimination of one of the experimental arms (high dose daily or the concomitant boost radiotherapy arm), due to greater toxicity.

Hypothesis B: Patients in the hyperfractionated accelerated dose (45 Gy) group will experience worse esophagitis (measured by the ECOG Acute Esophagitis Scale and difficulty swallowing item), and as a consequence worse psychological state, (measured by the Hospital Anxiety and Depression Scale), and lower quality-adjusted life years (measured by the EQ-5D) than those in the high dose daily (70 Gy) and concomitant boost radiotherapy (61.2 Gy) groups when all RT

has been completed in each arm of the study. This hypothesis will be revised to reflect the change in the research design due to the elimination of one of the experimental arms (high dose daily or the concomitant boost radiotherapy arm), due to greater toxicity.

<u>Hypothesis C</u>: Patients in the high dose daily 70 Gy arm of the study will experience greater treatment inconvenience (measured by a single item developed for this study), than those in the other two arms of the study, due to having to come into clinic for 7 weeks, the longest duration of the three arms. If the 70 Gy arm is eliminated due to greater treatment toxicity, then this hypothesis will be revised to reflect that those in the 61.2 Gy arm will experience greater treatment inconvenience than those in the 45 Gy arm, due to having to come into clinic for 5 weeks.

<u>Overview</u>:

In the quality of life companion, patients will be assessed by multiple HRQOL (Health-related quality of life) scales at baseline, 3, 5, 7, 12, 26 and 52 weeks (see <u>Table 1</u> for details). Investigators are interested in evaluating the change of quality of life assessments from baseline to multiple time points during and after treatment and comparing the differences of such changes between treatment regimens. It is expected that patients in arm 1 (45 Gy) experience worse physical and functional well being, worse esophagitis than those in the remaining study arm (arm 2 or 3), and that patients in arm 2 (70 Gy) experience worse treatment inconvenience than those in arm 1 (45 Gy) or arm 3 (61.2 Gy). The HRQOL scores changed from baseline to 12 week are of the primary scientific interest. The size of the HRQOL companion is chosen such that one has adequate power to test three sets of scientific hypotheses as shown in Table 2). The companion will register 415 patients (57%) from the 729 patients consenting to the main treatment study. Of the 415 patients, 394 patients are expected to meet the eligibility criteria. Of the 394 patients on the companion study, 188 patients are expected in each of the two remaining study arms and 18 patients are in the discontinued study arm.

Table 2
Quality of Life Assessment

QoL Endpoint Variable	Hypothesis	Measure	Statistical Analysis
**Primary Endpoints**<br>Physical Well-being, Functional Well-Being, Lung Cancer physical symptoms<br><br>FACT Esophageal Eating and Swallowing Subscales	Hypothesis A: At 12 weeks after the start of XRT, patients in the 45Gy arm will report worse physical symptoms and functioning than those in the high dose daily (70Gy) and concomitant boost RT (61.2 Gy) groups.	FACT-L (TOI)<br><br>FACT Esophageal Eating and Swallowing Subscales	Continuous scores/outcomes<br><br>T test;<br>Analysis of covariance;<br>Linear mixed effect model or general linear model with generalized estimating equations
**Secondary Endpoints**<br><br>Esophagitis	Hypothesis B: At 12 weeks after the start of XRT, patients in the 45Gy arm will experience worse esophagitis than those in the high dose daily (70Gy) and concomitant boost RT (61.2 Gy) groups.	ECOG Acute Esophagitis Scale<br><br>Difficulty swallowing	Dichotomized or ordinal scores/outcomes<br><br>Chi-square test;<br>Logistic regression;<br>Nonlinear mixed effect or generalized linear model with generalized estimating equations using log or multi-log link.
Psychological state	Hypothesis B: At 12 weeks after the start of XRT, patients in the 45Gy arm will report a worse psychological state than those in the high dose daily (70Gy) and concomitant boost RT (61.2 Gy) groups.	Hospital Anxiety and Depression Scale (HADS)	Continuous scores/outcomes<br><br>T test;<br>Analysis of covariance;<br>Line mixed effect model or general linear model with generalized estimating equations
Quality-adjusted Life Years (QALY)	Hypothesis B: At 12 weeks after the start of XRT, the QALY of those in the 45 Gy will be worse than those in the high dose daily (70Gy) and concomitant	EQ-5D	Quality adjusted time to event variable/outcome<br><br>Generalized log rank test for QALY data

	boost RT (61.2 Gy) groups.		
Treatment Inconvenience	Hypothesis C: At 7 weeks after the start of XRT, treatment inconvenience will be greatest for those receiving 70Gy QD in 7 weeks.	Single item developed for the study	Dichotomized or ordinal scores/outcomes<br><br>Chi-square test; Logistic regression; Nonlinear mixed effect or generalized linear model with generalized estimating equations using log or multi-log link.

Refusal and attrition rates have been found to vary by disease site, stage, type of treatment, method of data collection, and length of follow-up. Osoba and Zee [78] conducted a review of completion rates in health–related quality of life assessments of the National Cancer Institute of Canada Clinical Trials Group, in which data were collected by self-administered questionnaires. In seven completed trials, quality of life completion rates were high with more than 93% of patients completing questionnaires at baseline and while on treatment. The rate of completion off treatment was 85%. In Ganz et al.'s [90] study of the quality of life of early stage Hodgkin's disease patients, in which data was collected by mailed questionnaires, the completion rate by the second year was 70%. In a Gynecologic Oncology Group study of the quality of life of endometrial cancer patients randomized to either laparoscopy vs. laparotomy, in which data were collected by self-administered questionnaires, the completion rate by 6 months was 85% [91]. In a Cancer and Leukemia Group B study of patients with myelodysplastic syndrome treated by 5-Azacytidine (CALGB 9221) in which patients were interviewed by telephone about their quality of life, only 5% refused to continue the quality of life assessments over a 6 month study period [92]. In the Gynecologic Oncology Group trial of IP versus IV chemotherapy in ovarian cancer patients, where patients completed questionnaires in the clinic, 82% of IV and 79% of IP patients completed self-administered questionnaires at 12 months after treatment. Therefore, given these findings, it is reasonable to assume a completion rate of quality of life data of at least 80% at both baseline and 12 weeks after the start of radiation therapy, which ensures adequate statistical power in conducting the planned comparisons at the designated levels of effect size and Type I errors.

Power Analysis:

The following assumptions are made in the power calculations: (1) Based on the literature and previous CALGB studies, we expect that at least 80% of eligible patients who register to the companion will complete the HRQOL assessments at both baseline and 12 weeks after the start of radiation therapy. That is, of the 394 eligible patients on the companion study, 150 patients in the two remaining study arms and 15 in the discontinued study arm will have complete HRQOL assessments at both baseline and 12 weeks after the start of radiation therapy; (2) Within each set of hypothesis (A, B, C), Type I error will be controlled at the level of 0.05. The Bonferroni method is used to address the issue of multiple comparisons for simplicity and reasonable performance with a small number of comparisons; and (3) Two-sided tests will be used.

Hypothesis A

Three planned comparisons will be made to address Hypothesis A. The total TOI score from FACT TOI, the eating score and the swallowing score will be calculated from the FACT-Esophagus Subscale. The change scores between baseline and 12 weeks after the start of radiation therapy are of interest. A size of 150 patients with complete assessments at baseline and 12 weeks after the start of radiation therapy in each of the two remaining arms will have 85% power to detect an effect size of 0.397 s.d. unit on the scale of change score using a two group t-test at a significance level of 0.0167 (0.05/3). The effect size of 0.397 s.d. is usually associated with a moderate effect in quality of life literature.

Hypothesis B

Five comparisons will be made to address this hypothesis - ECOG esophagitis score (severe/≥7 vs moderate/<7), difficulty swallowing (severe/≥4 vs. moderate/<4), HADS depression score, HADS anxiety score, and quality-adjusted life year (QALY).

The ECOG esophagitis score ranges from 0 to 12 points. We will define patients with scores of ≥7 as “Severe” and score <7 as “Moderate.” With 150 patients in each remaining arm with complete assessments at baseline and 12 weeks after the start of radiation therapy, a two group continuity corrected Chi-square test at a 2-sided significance level of 0.01 (0.05/5) will have 80% power to detect the 18% difference of the proportions of patients with severe symptoms between the two remaining study arms, assuming 35% of patients in arm 1 report severe symptoms and 17% in the other remaining arm (odds ratio of 2.625).

We define swallowing difficulty score as 4 or 5 as “Severe” and scores 1, 2, or 3 as “Moderate.” Similar to the ECOG esophagitis score, with 150 patients in each study arm, we have 80% power to detect a 18% difference of severe swallowing difficulty between the two study arms, assuming 35% swallowing difficulty for arm 1 and 17% in the other arm.

The depression and anxiety scores of HADS are of primary interest. Consider the change score between baseline and 12 weeks after the start of radiation therapy. With 150 in each of the two remaining arms, one has 85% power to detect an effect size of 0.419 s.d. unit on the scale of change score using a two group t-test at a significance level of 0.01.

Quality-adjusted life years (QALY) is calculated as EQ-5D utility score weighted survival time. For the purpose of power calculation, consider a patient with a QALY greater than 12 weeks as “success” and less than 12 weeks as “failure.” With a size of 150 patients having EQ-5D score at baseline and 12 weeks, we have 90% power to detect a 20% difference of 12-week success rate, assuming the 12-week success rate is 65% for arm 1 (45 Gy) and 85% for the remaining experimental arm.

Hypothesis C

The different treatment arms have different dose schedules for different lengths of times, which may impact the satisfaction with treatment due to the inconvenience posed. Patients with an inconvenience rating of 1 or 2 will be defined as “Inconvenient” and those with a rating of 3 or 4 as “Convenient.” If the remaining experimental arm is arm 2 (70 Gy), with 150 patients having inconvenience measure on each arm at baseline and 12 weeks after the start of radiation therapy, a two group continuity corrected Chi-square test at a significance level of 0.05 will have 81% power to detect the 16% difference of the proportion of patients reporting treatment inconvenience between the two remaining study arms, assuming 60% of patients in arm 1 report treatment inconvenience and 76% of patients in arm 2 report inconvenience. If arm 2 is discontinued after 30 patients have been accrued to the main study, with about 150 patients in arm 1 and 15 patients in arm 2 having complete assessment at baseline and 12 weeks, we will have 74% power to detect the 30% difference of the proportion of patients reporting treatment inconvenience between the two remaining study arms, assuming 60% of patients in arm 1 report treatment inconvenience and 90% of patients in arm 2 report inconvenience.

Analytic Methods:

For continuous HRQOL (Health-Related Quality of Life) scores, including Physical Well-Being, Functional Well-Being, Lung Cancer Physical Symptoms subscale scores from the FACT-TOI, Eating and Swallowing subscale scores from the FACT-Esophagus Subscale, and depression and anxiety subscale scores from HADS, two-group t test and analysis of covariance will be the primary method used to analyze and test the planned comparisons for the change scores from baseline to 12 weeks after the start of radiation therapy with and without adjusting for other prognostic factors. In analysis of covariance, in addition to study arm, significant baseline prognostic factors will be included in the model to adjust for confounding effects. These planned tests results will be reported at the significance level designated in the section of power analysis. Similar exploratory univariate analyses will be conducted for the change score from baseline to other assessment points at 3, 5, 7, 26, and 52 weeks after the start of radiation therapy. Multivariate analyses for evaluating the trend of HRQOL scores varying over time will be conducted using a linear mixed effect model and linear model with generalized estimating equation (GEE) with all valid measures from multiple time points included in the analysis.

For binary dichotomized endpoints, such as ECOG esophagitis severity (severe/≥7 vs moderate/<7), difficulty swallowing (severe/≥4 vs. moderate/<4) and ordinal endpoints, such as treatment inconvenience (inconvenient/≤2 yes vs. convenient/≥3), Chi-square test and generalized linear model with a logit or multi-logit link will be used to test the planned comparisons. In regression analysis, baseline prognostic factors besides study arm will be included. Again, these planned tests will be reported at the significance level as designated in the section of power analysis. Similar multivariate analyses for evaluating the trend of HRQOL scores varying over time will be conducted using a nonlinear mixed effect model and generalized linear model with generalized estimating equation (GEE) with all valid measures from multiple time points included, in which logit link for dichotomized scores and multi-logit link for ordinal scores will be specified. In the analysis of ordinal scores, proportional odds assumptions will be tested.

It is expected that a proportion of quality of life data collected at follow-up assessments will be missing due to various reasons, including failing health of patients. However, the percentage is expected to be less than 10% above and beyond that missing due to death. If the dominant missing mechanism is missing completely at random (MCAR) above and beyond the effect of study condition assignment, then the results based on complete data analysis will be unbiased. However, sensitivity analyses using different statistical methods and assuming different missing mechanisms (MCAR, MAR and NMAR) will be used to evaluate the robustness of main study findings from multivariate data analyses.

The quality-adjusted overall survival will be calculated for each study arm based on the utility score from EQ-5D and the subgroup difference will be tested using the method developed by Zhao and Tsiatis [93].

15.8 CDUS Reporting

The Alliance Statistics and Data Center will submit quarterly reports to CTEP by electronic means using the Clinical Data Update System (CDUS).

16.0 ADVERSE EVENT REPORTING (AER)

Investigators are required by Federal Regulations to report serious adverse events as defined below. Investigators are required to notify the Alliance Central Office, the Study Chair, and their Institutional Review Board (IRB) if a patient has a reportable serious adverse event. The descriptions and grading scales found in the revised NCI Common Terminology Criteria for Adverse Events (CTCAE) version 5.0 will be utilized for serious AE reporting beginning April 1, 2018. This study will utilize version 4.0 for routine toxicity reporting. All appropriate treatment areas should have access to a copy of the CTCAE version 5.0. A copy of the CTCAE version 5.0 can be downloaded from the CTEP web site (http://ctep.cancer.gov). All reactions determined to be "reportable" in an expedited manner must be reported using the NCI Adverse Event Expedited Reporting System (CTEP-AERS).

16.1 CALGB 30610 Adverse Event Reporting Requirements

Phase 2 and 3 Trials: CTEP-AERS Expedited Reporting Requirements for Adverse Events That Occur Within 30 Days[1] of the Last Dose of Treatment

	Grade 1	Grade 2	Grade 2	Grade 3		Grade 3		Grades 4 & 5[2]	Grades 4 & 5[2]
	Unexpected and Expected	Unexpected	Expected	Unexpected		Expected		Unexpected	Expected
				with Hospitalization	without Hospitalization	with Hospitalization	without Hospitalization		
**Unrelated Unlikely**	Not Required	Not Required	Not Required	Not Required	Not Required	Not Required	Not Required	10 Calendar Days	10 Calendar Days
**Possible Probable Definite**	Not Required	Not Required	Not Required	Not Required	Not Required	Not Required	Not Required	10 Calendar Days	10 Calendar Days

[1] **Adverse events with attribution of possible, probable, or definite that occur greater than 30 days after the last dose of treatment require reporting as follows:**

CTEP-AERS 10 calendar day report:

- Grade 4 unexpected events
- Grade 5 expected or unexpected events

March 2005

Note: All deaths on study require both routine and expedited reporting regardless of causality. Attribution to treatment or other cause should be provided.

- Expedited AE reporting timelines defined:
  - "10 calendar days" - A complete CTEP-AERS report on the AE must be submitted within 10 calendar days of the investigator learning of the event.
- Any event that results in persistent or significant disabilities/incapacities, congenital anomalies, or birth defects must be reported via CTEP-AERS.

- Use the NCI protocol number and the protocol-specific patient ID provided during trial registration on all reports.

16.2 Additional Instructions or Exclusions from CTEP-AERS Expedited Reporting Requirements:

- Death due to progressive disease should be reported as Grade 5 "Disease progression" in the system organ class (SOC) "General disorders and administration site conditions." Evidence that the death was a manifestation of underlying disease (*e.g.*, radiological changes suggesting tumor growth or progression: clinical deterioration associated with a disease process) should be submitted.

  Any death occurring within 30 days of the last dose, regardless of attribution to the investigational agent/intervention requires expedited reporting within 24 hours.

  Any death occurring greater than 30 days after the last dose of the investigational agent/intervention requires expedited reporting within 24 hours only if it is possibly, probably, or definitely related to the investigational agent/intervention.
- All grade 4 events that are unexpected and that are at least possibly related to treatment must be reported via CTEP-AERS within 10 calendar days.
- Grade 4 events that are expected do not require CTEP-AERS expedited reporting, even if they result in hospitalization.
- Adverse events include those listed in Section 11.0 and in the package inserts.
- All adverse events reported via CTEP-AERS (i.e., serious adverse events) should also be forwarded to your local IRB.
- The reporting of adverse events described in the table above is in addition to and does not supplant the reporting of adverse events as part of the report of the results of the clinical trial, e.g., study summary forms or cooperative group data reporting forms (see Section 6.1 for required CALGB forms).
- Pregnancy loss is defined in CTCAE as "Death in utero." Any Pregnancy loss should be reported expeditiously, as Grade 4 "Pregnancy loss" under the Pregnancy, puerperium and perinatal conditions SOC. A Pregnancy loss should NOT be reported as a Grade 5 event under the Pregnancy, puerperium and perinatal conditions SOC, as currently CTEP-AERS recognizes this event as a patient death.

  A neonatal death should be reported expeditiously as Grade 4, "Death neonatal" under the General disorders and administration SOC.
- All new malignancies must be reported via CTEP-AERS whether or not they are thought to be related to either previous or current treatment. All new malignancies should be reported, i.e. solid tumors (including non-melanoma skin malignancies), hematologic malignancies, myelodysplastic syndrome/acute myelogenous leukemia, and in situ tumors. In CTCAE version 5.0, the new malignancies (both second and secondary) may be reported as one of the following: (1) Leukemia secondary to oncology chemotherapy, (2) Myelodysplastic syndrome, (3) Treatment-related secondary malignancy, or (4) Neoplasms benign, malignant and unspecified-other. Whenever possible, the CTEP-AERS reports for new malignancies should include tumor pathology, history or prior tumors, prior treatment/current treatment including duration, any associated risk factors or evidence regarding how long the new malignancy may have been present, when and how the new malignancy was detected, molecular characterization or cytogenetics of the original tumor (if available) and of any new tumor, and new malignancy treatment and outcome, if available.

17.0 References

1. Pignon, J.P., et al., A meta-analysis of thoracic radiotherapy for small-cell lung cancer. N Engl J Med, 1992. 327(23): p. 1618-24.

2. Warde, P. and D. Payne, Does thoracic irradiation improve survival and local control in limited-stage small-cell carcinoma of the lung? A meta-analysis. J Clin Oncol, 1992. 10(6): p. 890-5.

3. Janne, P.A., Freidlin, B., Saxman, S., et al., The survival of patients treated for limited stage small cell lung cancer in North America has increased during the past 20 years. Proc ASCO, 2001. 20: p. 317a.

4. Carney, D.N., J.B. Mitchell, and T.J. Kinsella, In vitro radiation and chemotherapy sensitivity of established cell lines of human small cell lung cancer and its large cell morphological variants. Cancer Res, 1983. 43(6): p. 2806-11.

5. Turrisi, A.T., 3rd, et al., Twice-daily compared with once-daily thoracic radiotherapy in limited small-cell lung cancer treated concurrently with cisplatin and etoposide. N Engl J Med, 1999. 340(4): p. 265-71.

6. Movsas, B., et al., Radiotherapy patterns of care study in lung carcinoma. J Clin Oncol, 2003. 21(24): p. 4553-9.

7. Choi, N.C., Herndon, J., Rosenman, J., Carey, R., Chung, C.T., Bogart, J., Seagren, S., Green, M., Long term survival data from CALGB 8837: radiation dose escalation and concurrent chemotherapy (CT) in limited stage small cell lung cancer (LD-SCLC). Possible radiation dose-survival relationship. Proc ASCO, 2002. 21: p. 1190.

8. Choi, N.C., et al., Phase I study to determine the maximum-tolerated dose of radiation in standard daily and hyperfractionated-accelerated twice-daily radiation schedules with concurrent chemotherapy for limited-stage small-cell lung cancer. J Clin Oncol, 1998. 16(11): p. 3528-36.

9. Bogart, J.A., et al., 70 Gy thoracic radiotherapy is feasible concurrent with chemotherapy for limited-stage small-cell lung cancer: analysis of Cancer and Leukemia Group B study 39808. Int J Radiat Oncol Biol Phys, 2004. 59(2): p. 460-8.

10. Fu, K.K., et al., A Radiation Therapy Oncology Group (RTOG) phase III randomized study to compare hyperfractionation and two variants of accelerated fractionation to standard fractionation radiotherapy for head and neck squamous cell carcinomas: first report of RTOG 9003. Int J Radiat Oncol Biol Phys, 2000. 48(1): p. 7-16.

11. Komaki, R., et al., Phase I study of thoracic radiation dose escalation with concurrent chemotherapy for patients with limited small-cell lung cancer: Report of Radiation Therapy Oncology Group (RTOG) protocol 97-12. Int J Radiat Oncol Biol Phys, 2005. 62(2): p. 342-50.

12. Phase II study of cisplatin and etoposide combined with accelerated high-dose thoracic radiotherapy in patients with limited stage small cell lung cancer. Protocol IDs: RTOG 02239 [cited June 23, 2006]; Available from: http://www.nci.nih.gov/clinicaltrials.

13. Fowler, J.F., The linear-quadratic formula and progress in fractionated radiotherapy. Br J Radiol, 1989. 62(740): p. 679-94.

14. Perry, M.C., et al., Thoracic radiation therapy added to chemotherapy for small-cell lung cancer: an update of Cancer and Leukemia Group B Study 8083. J Clin Oncol, 1998. 16(7): p. 2466-7.

15. Murray, N., et al., Importance of timing for thoracic irradiation in the combined modality treatment of limited-stage small-cell lung cancer. The National Cancer Institute of Canada Clinical Trials Group. J Clin Oncol, 1993. 11(2): p. 336-44.

16. Takada, M., et al., Phase III study of concurrent versus sequential thoracic radiotherapy in combination with cisplatin and etoposide for limited-stage small-cell lung cancer: results of the Japan Clinical Oncology Group Study 9104. J Clin Oncol, 2002. 20(14): p. 3054-60.

17. De Ruysscher, D., et al., Systematic review and meta-analysis of randomised, controlled trials of the timing of chest radiotherapy in patients with limited-stage, small-cell lung cancer. Ann Oncol, 2006. 17(4): p. 543-52.

18. Fried, D.B., et al., Systematic review evaluating the timing of thoracic radiation therapy in combined modality therapy for limited-stage small-cell lung cancer. J Clin Oncol, 2004. 22(23): p. 4837-45.

19. Einhorn, L.H., Initial therapy with cisplatin plus VP-16 in small-cell lung cancer. Semin Oncol, 1986. 13(3 Suppl 3): p. 5-9.

20. Roth, B.J., et al., Randomized study of cyclophosphamide, doxorubicin, and vincristine versus etoposide and cisplatin versus alternation of these two regimens in extensive small-cell lung cancer: a phase III trial of the Southeastern Cancer Study Group. J Clin Oncol, 1992. 10(2): p. 282-91.

21. Niell, H.B., et al., Randomized phase III intergroup trial of etoposide and cisplatin with or without paclitaxel and granulocyte colony-stimulating factor in patients with extensive-stage small-cell lung cancer: Cancer and Leukemia Group B Trial 9732. J Clin Oncol, 2005. 23(16): p. 3752-9.

22. Ettinger, D.S., et al., Study of paclitaxel, etoposide, and cisplatin chemotherapy combined with twice-daily thoracic radiotherapy for patients with limited-stage small-cell lung cancer: a Radiation Therapy Oncology Group 9609 phase II study. J Clin Oncol, 2005. 23(22): p. 4991-8.

23. Lyss, A.P., Herndon, J., Lynch, T., et al., Novel doublets in extensive stage small cell lung cancer: a randomized phase II study of topotecan plus cisplatin or paclitaxel (CALGB 9430). Clin Lung Cancer, 2001. 3: p. 205-210.

24. Noda, K., et al., Irinotecan plus cisplatin compared with etoposide plus cisplatin for extensive small-cell lung cancer. N Engl J Med, 2002. 346(2): p. 85-91.

25. Hanna, N., et al., Randomized phase III trial comparing irinotecan/cisplatin with etoposide/cisplatin in patients with previously untreated extensive-stage disease small-cell lung cancer. J Clin Oncol, 2006. 24(13): p. 2038-43.

26. Langer, C.J., Swamm, S., Werner-Wasik, R., et al., Phase I study of irinotecan (lr) and cisplatin (DDP) in combination with thoracic radiotherapy (RT), either twice daily (45 Gy) or once daily (70 Gy), in patients with limited (Ltd) small cell lung carcinoma (SCLC): early analysis of RTOG 0241. Proc ASCO, 2006. 24: p. 7058.

27. Paris, F., et al., Endothelial apoptosis as the primary lesion initiating intestinal radiation damage in mice. Science, 2001. 293(5528): p. 293-7.

28. Garcia-Barros, M., et al., Tumor response to radiotherapy regulated by endothelial cell apoptosis. Science, 2003. 300(5622): p. 1155-9.

29. Moeller, B.J., et al., Pleiotropic effects of HIF-1 blockade on tumor radiosensitivity. Cancer Cell, 2005. 8(2): p. 99-110.

30. Laberge, F., et al., Use of carcinoembryonic antigen in small cell lung cancer. Prognostic value and relation to the clinical course 1. Cancer, 1987. 59(12): p. 2047-52.

31. Slodkowska, J., et al., Expression of CEA and trophoblastic cell markers by lung carcinoma in association with histological characteristics and serum marker levels. Eur J Cancer Prev, 1998. 7(1): p. 51-60.

32. Sculier, J.P., et al., Carcinoembryonic antigen: a useful prognostic marker in small-cell lung cancer. J Clin Oncol, 1985. 3(10): p. 1349-54.

33. Jaques, G., et al., Prognostic value of pretreatment carcinoembryonic antigen, neuron-specific enolase, and creatine kinase-BB levels in sera of patients with small cell lung cancer. Cancer, 1988. 62(1): p. 125-34.

34. Fizazi, K., et al., Normal serum neuron specific enolase (NSE) value after the first cycle of chemotherapy: an early predictor of complete response and survival in patients with small cell lung carcinoma. Cancer, 1998. 82(6): p. 1049-55.

35. Jorgensen, L.G., et al., Serum neuron-specific enolase (S-NSE) and the prognosis in small-cell lung cancer (SCLC): a combined multivariable analysis on data from nine centres. Br J Cancer, 1996. 74(3): p. 463-7.

36. Johnson, P.W., et al., Tumour markers for prediction of survival and monitoring of remission in small cell lung cancer. Br J Cancer, 1993. 67(4): p. 760-6.

37. Tas, F., et al., Serum vascular endothelial growth factor (VEGF) and interleukin-8 (IL-8) levels in small cell lung cancer. Cancer Invest, 2006. 24(5): p. 492-6.

38. Fontanini, G., et al., Vascular endothelial growth factor is associated with neovascularization and influences progression of non-small cell lung carcinoma. Clin Cancer Res, 1997. 3(6): p. 861-5.

39. Imoto, H., et al., Vascular endothelial growth factor expression in non-small-cell lung cancer: prognostic significance in squamous cell carcinoma. J Thorac Cardiovasc Surg, 1998. 115(5): p. 1007-14.

40. Salven, P., et al., High pre-treatment serum level of vascular endothelial growth factor (VEGF) is associated with poor outcome in small-cell lung cancer. Int J Cancer, 1998. 79(2): p. 144-6.

41. Matsuyama, W., et al., Serum levels of vascular endothelial growth factor dependent on the stage progression of lung cancer. Chest, 2000. 118(4): p. 948-51.

42. Lloyd, K.P. and G.W. Krystal, Role of small-molecule kit receptor tyrosine kinase inhibitors in the treatment of small-cell lung cancer. Clin Lung Cancer, 2002. 3(3): p. 213-8.

43. Hibi, K., et al., Coexpression of the stem cell factor and the c-kit genes in small-cell lung cancer. Oncogene, 1991. 6(12): p. 2291-6.

44. Krystal, G.W., S.J. Hines, and C.P. Organ, Autocrine growth of small cell lung cancer mediated by coexpression of c-kit and stem cell factor. Cancer Res, 1996. 56(2): p. 370-6.

45. Rygaard, K., T. Nakamura, and M. Spang-Thomsen, Expression of the proto-oncogenes c-met and c-kit and their ligands, hepatocyte growth factor/scatter factor and stem cell factor, in SCLC cell lines and xenografts. Br J Cancer, 1993. 67(1): p. 37-46.

46. Sekido, Y., et al., Recombinant human stem cell factor mediates chemotaxis of small-cell lung cancer cell lines aberrantly expressing the c-kit protooncogene. Cancer Res, 1993. 53(7): p. 1709-14.

47. Mroczko, B., M. Szmitkowski, and J. Niklinski, Stem cell factor and granulocyte-macrophage-colony stimulating factor as candidates for tumour markers for non-small-cell lung cancer. Clin Chem Lab Med, 1999. 37(10): p. 959-62.

48. Fyles, A.W., et al., Oxygenation predicts radiation response and survival in patients with cervix cancer. Radiother Oncol, 1998. 48(2): p. 149-56.

49. Hockel, M., et al., Association between tumor hypoxia and malignant progression in advanced cancer of the uterine cervix. Cancer Res, 1996. 56(19): p. 4509-15.

50. Pugh, C.W. and P.J. Ratcliffe, Regulation of angiogenesis by hypoxia: role of the HIF system. Nat Med, 2003. 9(6): p. 677-84.

51. Semenza, G.L., Targeting HIF-1 for cancer therapy. Nat Rev Cancer, 2003. 3(10): p. 721-32.

52. Le, Q.T., et al., Identification of osteopontin as a prognostic plasma marker for head and neck squamous cell carcinomas. Clin Cancer Res, 2003. 9(1): p. 59-67.

53. Kuenen, B.C., et al., Potential role of platelets in endothelial damage observed during treatment with cisplatin, gemcitabine, and the angiogenesis inhibitor SU5416. J Clin Oncol, 2003. 21(11): p. 2192-8.

54. Kuenen, B.C., et al., Dose-finding and pharmacokinetic study of cisplatin, gemcitabine, and SU5416 in patients with solid tumors. J Clin Oncol, 2002. 20(6): p. 1657-67.

55. Norden-Zfoni, A., Manola, J., Desai, J., Morgan, J., Bello, C., Deprimo, S.E., Shalinsky, D.R., Baum, C., Demetri, G.D., Heymach, J.V., Levels of circulating endothelial cells (CECs) and monocytes as pharmacodynamic markers for SU11248 activity in patients (pts) with metastatic imatinib-resistant GIST. Proc ASCO, 2005. 24: p. 9036.

56. The first ten years of the World Health Organization, ed. W.H. Organization. Vol. p. 459. 1958, Geneva, Switzerland.

57. Schipper, H., Guidelines and caveats for quality of life measurement in clinical practice and research. Oncology (Williston Park), 1990. 4(5): p. 51-7; discussion 70.

58. Mehta, M.P., et al., Phase II trial of hyperfractionated accelerated radiation therapy for nonresectable non-small-cell lung cancer: results of Eastern Cooperative Oncology Group 4593. J Clin Oncol, 1998. 16(11): p. 3518-23.

59. Auchter, R.M., et al., Quality of life assessment in advanced non-small-cell lung cancer patients undergoing an accelerated radiotherapy regimen: report of ECOG study 4593. Eastern Cooperative Oncology Group. Int J Radiat Oncol Biol Phys, 2001. 50(5): p. 1199-206.

60. Bonner, J.A., et al., Phase III comparison of twice-daily split-course irradiation versus once-daily irradiation for patients with limited stage small-cell lung carcinoma. J Clin Oncol, 1999. 17(9): p. 2681-91.

61. Sloan, J.A., et al., A quality-adjusted reanalysis of a Phase III trial comparing once-daily thoracic radiation vs. twice-daily thoracic radiation in patients with limited-stage small-cell lung cancer(1). Int J Radiat Oncol Biol Phys, 2002. 52(2): p. 371-81.

62. Bailey, A.J., M.K. Parmar, and R.J. Stephens, Patient-reported short-term and long-term physical and psychologic symptoms: results of the continuous hyperfractionated accelerated [correction of acclerated] radiotherapy (CHART) randomized trial in non-small-cell lung cancer. CHART Steering Committee. J Clin Oncol, 1998. 16(9): p. 3082-93.

63. Auperin, A., et al., Prophylactic cranial irradiation for patients with small-cell lung cancer in complete remission. Prophylactic Cranial Irradiation Overview Collaborative Group. N Engl J Med, 1999. 341(7): p. 476-84.

64. Arriagada, R., et al., Prophylactic cranial irradiation for patients with small-cell lung cancer in complete remission. J Natl Cancer Inst, 1995. 87(3): p. 183-90.

65. Gregor, A., et al., Prophylactic cranial irradiation is indicated following complete response to induction therapy in small cell lung cancer: results of a multicentre randomised trial. United Kingdom Coordinating Committee for Cancer Research (UKCCCR) and the European Organization for Research and Treatment of Cancer (EORTC). Eur J Cancer, 1997. 33(11): p. 1752-8.

66. Darling, G., et al., Validation of the functional assessment of cancer therapy esophageal cancer subscale. Cancer, 2006. 107(4): p. 854-63.

67. Cella, D.F., et al., Reliability and validity of the Functional Assessment of Cancer Therapy-Lung (FACT-L) quality of life instrument. Lung Cancer, 1995. 12(3): p. 199-220.

68. Cella, D.F., et al., The Functional Assessment of Cancer Therapy scale: development and validation of the general measure. J Clin Oncol, 1993. 11(3): p. 570-9.

69. Brucker, P.S., et al., General population and cancer patient norms for the Functional Assessment of Cancer Therapy-General (FACT-G). Eval Health Prof, 2005. 28(2): p. 192-211.

70. Butt, Z., et al., Quality of life in lung cancer: the validity and cross-cultural applicability of the Functional Assessment Of Cancer Therapy-Lung scale. Hematol Oncol Clin North Am, 2005. 19(2): p. 389-420, viii.

71. Cella, D., et al., What is a clinically meaningful change on the Functional Assessment of Cancer Therapy-Lung (FACT-L) Questionnaire? Results from Eastern Cooperative Oncology Group (ECOG) Study 5592. J Clin Epidemiol, 2002. 55(3): p. 285-95.

72. Zigmond, A.S. and R.P. Snaith, The hospital anxiety and depression scale. Acta Psychiatr Scand, 1983. 67(6): p. 361-70.

73. Carroll, B.T., et al., Screening for depression and anxiety in cancer patients using the Hospital Anxiety and Depression Scale. Gen Hosp Psychiatry, 1993. 15(2): p. 69-74.

74. Hopwood, P., A. Howell, and P. Maguire, Screening for psychiatric morbidity in patients with advanced breast cancer: validation of two self-report questionnaires. Br J Cancer, 1991. 64(2): p. 353-6.

75. Razavi, D., et al., Screening for adjustment disorders and major depressive disorders in cancer in-patients. Br J Psychiatry, 1990. 156: p. 79-83.

76. Ibbotson, T., et al., Screening for anxiety and depression in cancer patients: the effects of disease and treatment. Eur J Cancer, 1994. 30A(1): p. 37-40.

77. Osoba, D., et al., Interpreting the significance of changes in health-related quality-of-life scores. J Clin Oncol, 1998. 16(1): p. 139-44.

78. Osoba, D. and B. Zee, Completion rates in health-related quality-of-life assessment: approach of the National Cancer Institute of Canada Clinical Trials Group. Stat Med, 1998. 17(5-7): p. 603-12.

79. Shaw, J.W., J.A. Johnson, and S.J. Coons, US valuation of the EQ-5D health states: development and testing of the D1 valuation model. Med Care, 2005. 43(3): p. 203-20.

80. Johnson, J.A., et al., Valuation of EuroQOL (EQ-5D) health states in an adult US sample. Pharmacoeconomics, 1998. 13(4): p. 421-33.

81. Holland, J.C., Herndon, J., Kornblith, A.B., et al., A sociodemographic data collection model for cooperative clinical trials. Proc ASCO, 1992. 11(Abstract No. 445): p. 157.

82. Zelen, M., The randomization and stratification of patients to clinical trials. J Chronic Dis, 1974. 27(7-8): p. 365-75.

83. Emerson, S., S+SEQTRIAL: technical overview. Research Report No. 98, Data Analysis Products Division, MathSoft, Inc. 2000.

84. O'Brien, P.C. and T.R. Fleming, A multiple testing procedure for clinical trials. Biometrics, 1979. 35(3): p. 549-56.

85. Freidlin, B., E.L. Korn, and S.L. George, Data monitoring committees and interim monitoring guidelines. Control Clin Trials, 1999. 20(5): p. 395-407.

86. Cox, D.R., Regression models and life-tables. J R Strat Soc [B], 1972. 34: p. 187-220.

87. Cox, D.R., Snell, E.J., Analysis of binary data. 2nd ed. 1989, London: Chapman & Hall.

88. Lausen, B., Schumacher, M., Maximally selected rank statistics. Biometrics, 1992. 48: p. 73-85.

89. Kaplan, E.L., Meier, P., Nonparametric estimation from incomplete observations. J Am Stat Assoc, 1958. 53: p. 457-481.

90. Ganz, P.A., et al., Health status and quality of life in patients with early-stage Hodgkin's disease treated on Southwest Oncology Group Study 9133. J Clin Oncol, 2003. 21(18): p. 3512-9.

91. Kornblith, A.B., Walker, J., Huang, H., Cella D., Quality of life of patients in a randomized clinical trial of laparoscopy versus open laparotomy for the surgical resection and staging of uterine cancer: a Gynecology Oncology Group study. Gynecologic Oncology, 2006. 101(Suppl (1)): p. S22-S23.

92. Kornblith, A.B., et al., Impact of azacytidine on the quality of life of patients with myelodysplastic syndrome treated in a randomized phase III trial: a Cancer and Leukemia Group B study. J Clin Oncol, 2002. 20(10): p. 2441-52.

93. Zhao, H. and A.A. Tsiatis, Testing equality of survival functions of quality-adjusted lifetime. Biometrics, 2001. 57(3): p. 861-7.

94. Miyake Y, Kodama T, and Yamguchi K. Pro-gastrin-releasing peptide (31-98) is a specific tumor marker in patients with small cell lung carcinoma. Ca Res 34:2136-2140, 1994.

95. Okusaka T et al. Serum level o pro-gastrin-releasing peptide for follow-up of patients with small cell lung cancer. Clin Ca Res 3:123-127, 1997.

96. Molina R et al. Pro-gastrin-releasing peptide (ProGRP) in patients with benign and malignant disease: Comparison with CEA, SCC, CYFRA 21.1 and NSE in patients with lung cancer. Anticancer Res. 25:1773-1778, 2005.

97. Molina R et al. Usefulness of serum tumor markers, including progastrin-releasing peptide, in patients with lung cancer: correlation with histology. Tumor Biol 30:121-129, 2009.

98. Schneider J. Tumor markers in lung cancer. Advances in Clin Chem 42:1-41, 2006.

99. Skarlos DV, Samantas E, et al. Randomized comparison of etoposide-carboplatin and irradiation in small-cell lung cancer Ann Oncol. 1994 Sep;5(7):601-7.

Statistical Plan for Secondary Analysis

Statistical Analysis

Associations between patient, disease-related, treatment, social factors and PFS and OS were examined. Initial analyses employed univariate Cox proportional hazards (PH) models to assess the relationship between individual prognostic factors and survival. Factors displaying significant association ($p < 0.05$) in univariate analysis were incorporated into a multivariate Cox PH model, allowing for adjustment of confounding variables and inclusion of factors independently associated with survival outcomes. P-values from Wald tests for univariate models and likelihood ratio tests for multivariate models were reported. Hazard ratios and the corresponding confidence intervals were estimated from the multivariable Cox proportional hazards (PH) model. Kaplan-Meier survival analysis was used to generate survival curves and to estimate median survival times. Subgroup analyses were conducted to explore heterogeneity in treatment effects across patient subpopulations defined by patient, disease, treatment, and social factors. Difference in survival was performed using log-rank tests and hazard ratios and their confidence intervals were estimated from univariate Cox PH models. Patients with missing values in the baseline risk factors were excluded. Descriptive statistics were reported when patient demographic data was summarized by arm. Associations of continuous variables with treatment arm were assessed via Wilcoxon rank-sum tests and associations of categorical variables via chi-square tests. Subset analyses did not require all variables as in the multivariable assessment; thus patient numbers differ from those of Figure 1.

Statistical significance was defined as a two-sided p-value < 0.05. All statistical analyses were performed by the Alliance Statistics and Data Management Center utilizing SAS 9.4 (Cary, NC). Data were locked May-2020.
