# Supplementary material for: Prognostic Factors in Limited-Stage Small Cell Lung Cancer: A Secondary Analysis of CALGB 30610–RTOG 0538
Source: JAMA Netw Open. Author manuscript; Available in PMC 2025 Oct 1. (PMC11581554; doi:10.1001/jamanetworkopen.2024.40673)
Supplement: Supplement 2 [file NIHMS2034183-supplement-Supplement_2.pdf]

Data Sharing Statement

Farris. Prognostic Factors in Limited-Stage Small-Cell Lung Cancer. *JAMA Netw Open*. Published October 24, 2024. doi:10.1001/jamanetworkopen.2024.40673

Data
Additional Information: Trial Registration: ClinicalTrials.gov identifier: NCT00632853 https://www.clinicaltrials.gov/study/NCT00632853
Data available: Yes
Data types: Other (please specify)
Additional Information: Data Sharing Statement: De-identified participant data and the associated data dictionary for CALGB 30610 are available at https://nctn-data-archive.nci.nih.gov/. De-identified patient data not publicly available may be requested from Alliance for Clinical Trials in Oncology via Datasharing@alliancenctn.org. A formal review process includes verifying the availability of data, reviewing any existing agreements that may have implications for the project, and ensuring that any transfer is in compliance with the IRB. The investigator must sign a data release form prior to transfer.
How to access data: Data Sharing Statement: De-identified participant data and the associated data dictionary for CALGB 30610 are available at https://nctn-data-archive.nci.nih.gov/. De-identified patient data not publicly available may be requested from Alliance for Clinical Trials in Oncology via Datasharing@alliancenctn.org. A formal review process includes verifying the availability of data, reviewing any existing agreements that may have implications for the project, and ensuring that any transfer is in compliance with the IRB. The investigator must sign a data release form prior to transfer.
When available: With publication

Supporting Documents
Document types: None

Additional Information
Who can access the data: Anyone, but must ask the Alliance Foundation
Types of analyses: Must request and specify purpose through Alliance
Mechanisms of data availability: after approval of proposal to Alliance
